# Evaluation of Methods to Assess *in vivo* Activity of Engineered Genome-Editing Nucleases in Protoplasts

**DOI:** 10.3389/fpls.2019.00110

**Published:** 2019-02-08

**Authors:** Satya Swathi Nadakuduti, Colby G. Starker, Dae Kwan Ko, Thilani B. Jayakody, C. Robin Buell, Daniel F. Voytas, David S. Douches

**Affiliations:** ^1^Department of Plant, Soil and Microbial Sciences, Michigan State University, East Lansing, MI, United States; ^2^Department of Genetics, Cell Biology and Development and Center for Precision Plant Genomics, University of Minnesota, Saint Paul, MN, United States; ^3^Department of Plant Biology, Michigan State University, East Lansing, MI, United States; ^4^Plant Resilience Institute, Michigan State University, East Lansing, MI, United States; ^5^Michigan State University AgBioResearch, Michigan State University, East Lansing, MI, United States

**Keywords:** genome-editing, CRISPR/Cas9, TALENs, protoplasts, double-stranded oligodeoxynucleotides, NHEJ

## Abstract

Genome-editing is being implemented in increasing number of plant species using engineered sequence specific nucleases (SSNs) such as Clustered Regularly Interspaced Short Palindromic Repeats/CRISPR-associated systems (CRISPR/Cas9), Transcription activator like effector nucleases (TALENs), and more recently CRISPR/Cas12a. As the tissue culture and regeneration procedures to generate gene-edited events are time consuming, large-scale screening methodologies that rapidly facilitate validation of genome-editing reagents are critical. Plant protoplast cells provide a rapid platform to validate genome-editing reagents. Protoplast transfection with plasmids expressing genome-editing reagents represents an efficient and cost-effective method to screen for *in vivo* activity of genome-editing constructs and resulting targeted mutagenesis. In this study, we compared three existing methods for detection of editing activity, the T7 endonuclease I assay (T7EI), PCR/restriction enzyme (PCR/RE) digestion, and amplicon-sequencing, with an alternative method which involves tagging a double-stranded oligodeoxynucleotide (dsODN) into the SSN-induced double stranded break and detection of on-target activity of gene-editing reagents by PCR and agarose gel electrophoresis. To validate these methods, multiple reagents including TALENs, CRISPR/Cas9 and Cas9 variants, eCas9(1.1) (enhanced specificity) and Cas9-HF1 (high-fidelity1) were engineered for targeted mutagenesis of *Acetolactate synthase1* (*ALS1*), *5-Enolpyruvylshikimate- 3-phosphate synthase1* (*EPSPS1*) and their paralogs in potato. While all methods detected editing activity, the PCR detection of dsODN integration provided the most straightforward and easiest method to assess on-target activity of the SSN as well as a method for initial qualitative evaluation of the functionality of genome-editing constructs. Quantitative data on mutagenesis frequencies obtained by amplicon-sequencing of *ALS1* revealed that the mutagenesis frequency of CRISPR/Cas9 reagents is better than TALENs. Context-based choice of method for evaluation of gene-editing reagents in protoplast systems, along with advantages and limitations associated with each method, are discussed.

## Introduction

Genome-editing by engineered sequence specific nucleases (SSNs) is a technological breakthrough that enables precise alterations to DNA, representing a new frontier in genetics. SSNs such as Clustered Regularly Interspaced Short Palindromic Repeats/CRISPR-associated systems (CRISPR/Cas9), Transcription activator like effector nucleases (TALENs), and more recently CRISPR/Cas12a (Cpf1, CRISPR from *Prevotella* and *Francisella* 1) generate double stranded breaks (DSBs) at pre-defined genomic loci. Furthermore, variants of SpCas9, SpCas9-HF1 (high-fidelity1) (Kleinstiver et al., 2015), eSpCas9 1.1 (enhanced specificity) (Hsu et al., 2013), HypaCas9 (hyper-accurate Cas9) (Chen J.S. et al., 2017) and evoCas9 (evolved Cas9) (Casini et al., 2018) have been designed based on structure-guided protein engineering to reduce non-specific DNA interactions thereby minimizing genome-wide off-targets. The DSBs are repaired either by non-homologous end-joining (NHEJ), at times creating insertion/deletions that may knock out gene function, or by homology directed repair (HDR) using a repair donor template resulting in gene editing. Recently, genome-editing has been expanded to a number of plant species including model and crop species, and in some cases genome-editing has created agronomically valuable traits (Shukla et al., 2009; Li et al., 2012, 2013; Haun et al., 2014; Shan et al., 2014; Wang et al., 2014; Woo et al., 2015; Clasen et al., 2016; Malnoy et al., 2016; Waltz, 2016; Braatz et al., 2017; Cermak et al., 2017; Chen X. et al., 2017; Liang et al., 2017; Soyk et al., 2017; Zong et al., 2017).

Genome-editing reagents are delivered into plant cells via *Agrobacterium*-mediated transformation, protoplast transfection, or particle bombardment and typically, selection is employed to regenerate plants with integrated constructs, which intend to induce desired mutations (Yin et al., 2017). Plant transformation and regeneration processes from engineered cells and tissues typically require long and tedious tissue culture procedures. Thus, having access to a rapid method to test reagent activity prior to regeneration would be beneficial. Protoplasts isolated from plant tissue by enzymatic digestion of cell walls provide a platform to validate genome-editing reagents rapidly and each viable protoplast cell is totipotent, potentially capable of regenerating into a whole plant. Protoplasts have been used as a versatile tool for conducting cell based assays, analyzing diverse signaling pathways, studying functions of cellular machineries, and functional genomics screening (Sheen, 2001; Yoo et al., 2007; Ondřej et al., 2009; Xiao et al., 2012; Xing and Wang, 2015). There are several advantages to a protoplast system for screening genome-editing reagents. First, isolation and transformation of protoplasts can be performed in less than a week. Second, protoplast transformation is a direct means to deliver genome-editing reagents and does not require a biological vector. Third, protoplasts can be used to generate thousands of independent events and aid in regenerating plants without incorporation of any foreign DNA (Haun et al., 2014; Clasen et al., 2016). Fourth, protoplasts can be used to detect reporter genes by microscopy and are amenable to cell sorting (Xing and Wang, 2015).

Genome-editing using protoplast transformation and regeneration of whole plants with targeted modifications has been reported in various plant species (Li et al., 2013; Shan et al., 2014; Wang et al., 2014; Sun et al., 2015; Woo et al., 2015; Clasen et al., 2016; Malnoy et al., 2016; Kim et al., 2017; Liang et al., 2017). Recently, CRISPR/Cas12a and base editing systems along with DNA-free CRISPR delivery methods such as pre-assembled ribonucleoproteins (RNPs) have also been implemented in protoplasts to achieve targeted mutagenesis and whole plant regeneration (Woo et al., 2015; Kim et al., 2017; Zong et al., 2017; Andersson et al., 2018).

Various approaches that aid in validation of genome-editing reagent constructs and detection of targeted mutagenesis in protoplasts prior to proceeding with time-consuming tissue culture transformation and regeneration procedures are available including the PCR/restriction enzyme digestion assay (PCR/RE) assay, the T7 endonuclease I (T7E1) assay and deep sequencing of targeted amplicons (Shan et al., 2014; Kim et al., 2017). For the PCR/RE assay, target amplicons are digested with restriction enzyme that can recognize wild type but not mutagenized sequence since the site is disrupted by SSNs. For the T7EI assay, the amplicons are denatured and re-annealed to form heteroduplexes that can be cleaved by T7EI. Deep sequencing of the target amplicons efficiently identifies targeted mutagenesis with high sensitivity. Besides these approaches, an alternative strategy for evaluation of *in vivo* activity of engineered genome-editing nucleases in protoplasts is described here. In this method that is adapted from the Genome-wide Unbiased Identification of Double stranded breaks Enabled by sequencing (GUIDE-seq) approach (Tsai et al., 2015), on-target activity of reagents can be evaluated by integrating blunt double-stranded oligodeoxynucleotides (dsODNs) into the DSBs induced at target regions by a simple PCR reaction. CRISPR/Cas9, variants of Cas9, eCas9(1.1), Cas9-HF1 and TALEN reagents were engineered to target two loci, *Acetolactate synthase1* (*ALS1*) and *5-Enolpyruvylshikimate- 3-phosphate synthase* (*EPSPS1*) and their paralogs in potato. In summary, this method of dsODN integration and PCR detection can be used as a first evaluation step to rapidly screen multiple engineered SSNs in order to discard the non-functional reagents. All the tested methods are performed on common targets to further validate, compare, and quantify the targeted mutagenesis caused by SSNs and are widely applicable to any plant species in which protoplast isolation and transformation procedures are established.

## Materials and Methods

### Vector Construction and Mutagenesis Using CRISPR/Cas9 and TALENs

*Acetolactate synthase1* and *EPSPS* genes were cloned and sequenced from the potato line DMRH-S5 28-5 (Peterson et al., 2016). Single-guide RNA spacers targeting *ALS* (Butler et al., 2015) and *EPSPS* were designed in the coding sequence of target genes using CRISPR RGEN tools^1^. Equimolar amounts of sgRNA oligonucleotides are phosphorylated using polynucleotide kinase and T4 DNA ligase buffer and annealed together by boiling the reaction in a water bath for 3 min and letting it gradually cool down to room temperature. Double-strand sgRNAs were cloned into sgRNA expression vectors using modular assembly with the Golden Gate cloning system as described previously (Cermak et al., 2017). Module A vector, pMOD_A0101 (Addgene #90998) was used for AtCas9 expression cassette. eCas9(1.1) and Cas9-HF1 (pMOD_A6101 and pMOD_A6201, respectively) were made by amplification of the vector sequence from pMOD_A0101 for the backbone and most part of AtCas9 and sequence specific for Cas9 variants, was synthesized and fragments joined via Gibson assembly (Gibson et al., 2009). Module B vector, pMOD_B2515 (Addgene #91072) was used to clone sgRNAs downstream of AtU6 promoter. Module C vector, pMOD_C3006 (Addgene #91094) was used for green fluorescent protein (GFP) expression that was driven by the FMV34S promoter. These A, B, and C modules were assembled into a transformation backbone vector pTRANS_100 (Addgene #91198) for protoplast transformation. Target TALEN binding sites were designed using TAL Effector Nucleotide Targeter 2.0 and constructed according to Cermak et al. (2011, 2017) using Golden Gate cloning with NΔ152/C63 N- and C-terminal truncations, respectively, and P2A translational skipping sequence. Module A vector, pMOD_A1001 (Addgene #90998) and Module B vector, pMOD_B2000 (Addgene #91059) were used for TALEN constructs. All vector construction procedures were according to Cermak et al. (2017).

### Plant Material and Growth Conditions

All the experiments in this study were conducted using a diploid self-compatible potato line DMRH S5 28-5 developed by crossing *S. tuberosum* L. Group Phureja DM 1-3 516 R44 [DM] and *S. tuberosum* L. Group Tuberosum RH89-039-16 [RH] (Peterson et al., 2016) and by selfing the fertile hybrid progeny for five generations. *In vitro* propagation and plant growth conditions are according to Nadakuduti et al. (2019). Briefly, plants are propagated using nodal cuttings on MS prop media (MS basal salts plus vitamins, 3% sucrose, 0.7% agar, pH 5.8) in tissue culture and grown in Magenta boxes in growth chambers with 16-h-light/8-h-night photoperiod at 22°C and average light intensity of 300 μmoles m^-2^ s^-1^.

### Protoplast Isolation, Viability Evaluation, and Density Estimation

Protoplasts were isolated from immature leaves of 4-week-old *in vitro* propagated DMRH S5 28-5 leaves as described previously (Cheng and Veilleux, 1991) with some modifications. Briefly, strips of leaves were excised directly into pre-plasmolysis medium (Supplementary Table S1) in the dark at room temperature for at least 4 h. Then, the leaf strips were digested in 1.25% (w/v) Cellulase R-10 ‘Onozuka,’ 0.05% (w/v) Macerozyme R-10, half-strength MS salts and vitamins, 0.4 M mannitol and 0.1 M glucose and incubated at 28°C at 60 rpm in dark for 16–22 h. The digested product was filtered through a sterile 40 μm cell strainer, diluted with autoclaved rinse medium (0.3 M KCl + 5 mM CaCl_2_) and centrifuged at room temperature for 5 min at 50 ×*g*. The protoplast pellet was re-suspended in autoclaved flotation medium (0.5 M sucrose + 5 mM CaCl_2_) and layered on top of the protoplast suspension with 3 mL of rinse medium. After centrifugation at 50 ×*g* for 10 min, intact protoplasts were collected at the interface of these two solutions. Protoplasts are rinsed and re-suspended in culture medium (Supplementary Table S1) depending upon the size of the pellet. The density of living protoplasts was determined using a hemacytometer as described previously (Yoo et al., 2007) and adjusted to the desired density (10^6^mL^-1^) with culture medium.

The viability of the protoplasts was evaluated by staining with 1% (w/v) Evans blue (Gaff and Okong’o-ogola, 1971). The percentage of viable protoplasts was determined by dividing the number of unstained cells by the total cell count. Bright field images of the Evans blue stained protoplasts were acquired using Nikon C2 microscope with Nikon DS-U3 color camera having 20x UPlanSApo objective (NA 0.75). Nikon NIS Elements software version 5.00.00 was used to acquire and analyze images.

### PEG-Mediated Transformation of Protoplasts

Protoplast transformation was performed based on the protocol described previously (Yoo et al., 2007) with some modifications. The CRISPR/Cas9 and TALENs constructs were transformed into 100 μL of protoplast solution (containing 10^5^ protoplasts). To account for the differences in size of the plasmid constructs, varied amounts of each plasmid was used to ensure 2.57 pmol concentration in the transformations. 100 pM of annealed oligonucleotides (Tsai et al., 2015) (2 μL of the prepared 50 pmol/μL) were mixed in with 2.57 pmol plasmid DNA per transformation reaction. Transformations with and without dsODNs in the reaction were carried out.

Protoplasts were gently mixed with 40 μL of plasmid DNA in the solution and 240 μL of filter sterilized 40% (w/v) polyethylene glycol transformation buffer (40% w/v PEG4000, 200 mM Mannitol, 100 mM CaCl_2_, pH 5.8) in round bottom 2 mL tubes. After 10 min incubation at room temperature, transformation was terminated by adding 900 μL of culture medium. Protoplasts were then collected by centrifuging for 5 min at 250 ×*g* at room temperature and washed one more time with 800 μL of culture medium by centrifuging for another 5 min at 250 ×*g*. Protoplasts were re-suspended in 200 μL of culture medium and placed in the dark at room temperature for 48 h before the genomic DNA isolation.

### GFP Detection Using Confocal Laser Scanning Microscope

Green fluorescent protein signaling Images were taken 24 h after transformation. Olympus FluoView 1000 Confocal Laser Scanning Microscope (Olympus America, Inc., Center Valley, PA, United States) configured on a fully automated Olympus IX81 inverted microscope with a 10x UPlanSApo objective (NA 0.40) was used to collect the GFP fluorescence (green) from a single confocal plane as well as the corresponding transmitted laser light brightfield image (gray). GFP fluorescence was excited using the 488 nm Argon gas laser line and detected using a 505–525 nm band pass emission filter.

### Protoplast Genomic DNA Isolation

After 48 h post-transformation, genomic DNA was isolated from three biological replicates, each replication being a pool of six transformation reactions using the hexadecyltrimethylammonium bromide (CTAB)-based method (Porebski et al., 1997). The genomic DNA was used as a template for dsODN insertion assay, T7EI, PCR/RE assays and amplicon sequencing library preparation.

### T7EI and PCR/RE Assays

The target regions of *ALS1* and *EPSPS1* were amplified using 10 ng of protoplast genomic DNA by Phusion High-Fidelity DNA Polymerase (Thermo Scientific). The PCR products were purified (Promega Wizard SV gel and PCR clean-up system) and quantified with using NanoDrop^TM^ 2000c spectrophotometer (Thermo Fisher, Wilmington, DE, United States). 1 μg of the purified PCR product was subjected to T7EI assay and PCR/RE assay.

The T7EI assay was performed as per the manufacturer’s instructions (New England Biolabs). Briefly, the PCR products were denatured at 95°C, re-annealing was carried out by ramp PCR from 95 to 85°C at -2°C/s and 85 to 25°C at -0.1°C/s. These annealed PCR products were incubated with T7 endonuclease I (NEB) at 37°C for 1 h and analyzed via 2% (w/v) agarose gel electrophoresis. PCR bands were quantified using ImageJ software^2^ and the mutagenesis frequencies for SSNs were estimated using percent gene modification = 100 x (1 - (1–fraction cleaved)^1/2^) (Guschin et al., 2010). The percentage of indels was calculated based on the relative intensity of the DNA bands using ImageJ software. The normalized indel percentage was calculated by dividing the indel percentage of the mutagenized samples with the WT.

For PCR/RE assay, PCR product of *ALS1* and *EPSPS1* are digested with *Bs*lI and *Xcm*I restriction enzymes respectively. Products were analyzed by agarose gel electrophoresis. The resistant band in both cases were cloned using Zero Blunt TOPO PCR cloning kit and Sanger sequenced to determine the mutant alleles present.

### Double-Stranded Oligodeoxynucleotide (dsODN) Preparation

Two modified oligonucleotides with 5^′^ phosphorylation (P)and 5^′^ and 3^′^ phosphorothioate linkages (^∗^) were synthesized byIDT^3^ (5^′^- P-G^∗^T^∗^TTAATTGAGTTGTCATATGTTAATAACGGT^∗^A^∗^T -3^′^ and 5^′^- P-A^∗^T^∗^ACCGTTATTAACATATGACAACTCAATTAA^∗^A^∗^C -3^′^). The blunt-ended dsODNs used in the study are prepared by annealing 100 μL of each oligonucleotide (100 μM) as described in vector construction to yield 50 pmol/μL dsODNs.

### On-Target Detection of dsODN by PCR

To detect the insertion of dsODNs at the DSB in the target regions, four different PCR reactions were performed with a combination of one dsODN specific primer and one gene specific primer along with a control reaction using gene-specific primers. The primers used for all the assays and to detect dsODNs at the target site are listed in Supplementary Table S2.

### AmpSeq: Illumina Library Preparation and Sequencing Analysis

Genomic DNA extracted from transfected protoplasts for each of biological triplicates, and triplicated Indexed Illumina DNA sequencing libraries were constructed using a two-step PCR method. Primary PCR amplicons spanning the *ALS1* cleavage site, of which, the expected size was 365-bp without an indel, were amplified using the gene specific primers (Supplementary Table S2) with the following conditions: 98°C for 30 s; 14 cycles (98°C 10 s, 60°C 30 s, 72°C 30 s); 72°C for 5 min; hold at 12°C. The gene specific primers contained 5^′^ tails that were used to add the Illumina adaptor sequences onto the primary amplicons. Secondary PCR amplicons, of which, the expected size was 426-bp without an indel, were then amplified using primers that added 6-nt indexing barcodes and Illumina adaptor sequence to the secondary PCR amplicons (Conditions: 98°C for 30 s; 14 cycles (98°C 10 s, 50°C 30 s, 72°C 30 s); 72°C for 5 min; hold at 12°C). The secondary PCR amplicon samples were individually purified using AMPure XP beads according to manufacturer’s instruction (Beckman Coulter, Brea, CA, United States); all purified samples were pooled with an equal molar ratio and run on a 1% agarose gel. The expected size bands were extracted and purified using The QIAquick Gel Extraction Kit (Qiagen, Valencia, CA, United States) according to the manufacturer’s instruction. The libraries were then purified using AMPure XP beads according to manufacturer’s instruction (Beckman Coulter, Brea, CA, United States) and the size distribution of amplicons was determined with the Agilent Bioanalyzer (Agilent Technologies, Palo Alto, CA, United States). The complete libraries were sequenced in 250 nt paired-end mode on the Illumina MiSeq using a MiSeq Reagent Nano kit ver2 (Illumina, San Diego, CA, United States) at the Research Technology Support Facility Genomics Core at Michigan State University. During the library construction, all PCR reactions were performed with Phusion High-Fidelity DNA Polymerase (New England BioLabs, Beverly, MA, United States). The list of primer sequences used in the library construction is provided in Supplementary Table S2.

For each library, read quality was accessed using the FastQC software (version 0.11.5)^4^. Reads were cleaned for quality and adapters were removed with Cutadapt software (version 1.8.1) (Martin, 2011) using minimum base quality of 20 retaining reads with a minimum length of 200 nucleotides after trimming. Cleaned reads were used for downstream analysis using CRISPResso (Pinello et al., 2016). For CRISPResso analysis, the sgRNA sequence (5^′^-CTACCTATGATTCCCAG-3^′^) was provided and the window size of 15 bp around the cleavage site was used to quantify NHEJ events. To identify reads containing dsODN, the paired-end reads were merged using FLASH software (version 1.2.11) (Magoc and Salzberg, 2011), and then converted to FASTA format using a custom script (5^′^-GTTTAATTGAGTTGTCATATGTTAATAACGGTAT-3^′^), the full sequence of dsODN, was searched against the merged reads using NCBI BLAST (version 2.6.0) (Altschul et al., 1990). Any reads that contained the full or partial dsODN sequence were counted.

### Statistical Analyses

The statistical analyses for transformation efficiencies, NHEJ efficiencies, mutations types and mutagenesis were performed by Student’s *t-*tests (two-tailed) or Duncan’s Multiple Range tests using SAS (SAS Institute ^5^).

## Results

### CRISPR/Cas9 and TALENs for Targeted Mutagenesis of *ALS* and *EPSPS* in Potato

**FIGURE 1:**
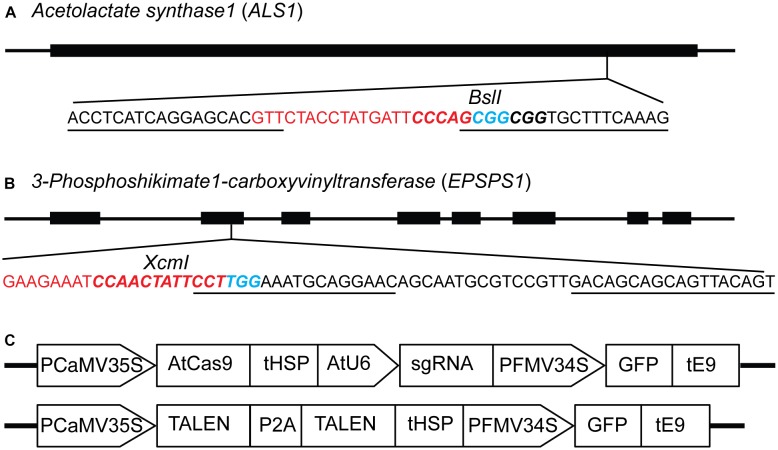
Constructs for targeted mutagenesis of *ALS1* and *EPSPS1* loci in potato protoplasts using CRISPR/Cas9 and TALEN reagents. **(A)** Map of potato target gene *Acetolactate synthase1* (*ALS1*) and **(B)** *3-Phosphoshikimate 1-carboxyvinyltransferase* (*EPSPS1*) used in the study. The sequence of the target site is shown with sgRNA spacer in red, PAM in blue, TALEN binding sites are underlined, and restriction enzyme sites are in bold italicized. **(C)** Structure of the constructs used for expressing sgRNAs and TALENs co-expressing green fluorescent protein (GFP) for both target genes. PCaMV35S, cauliflower mosaic virus 35S promoter, AtCas9, Arabidopsis codon optimized Cas9 nuclease; tHSP, Arabidopsis heat shock protein 18.2 terminator; AtU6, Arabidopsis U6 promoter; sgRNA, single guide RNA*;* PFMV34S, figwort mosaic virus 34S promoter; GFP, green fluorescent protein; tE9, pea ribulose bisphosphate carboxylase small subunit terminator; P2A, ribosomal skipping sequence.

The *ALS* and *EPSPS* genes are primary targets for different classes of herbicides, and specific mutations are known to confer resistance to the imidazolinone group of herbicides and glyphosate, respectively (Sathasivan et al., 1991; Baerson, 2002; Powles and Preston, 2006). Single guide RNAs (sgRNAs) and TALENs were designed for targeted mutagenesis of *ALS1* (Butler et al., 2015), *EPSPS1* (Figure 1A,B) and their paralogs, *ALS2* and *EPSPS2* respectively, in potato (Supplementary Table S3). The expression cassettes of SSNs used for protoplast transient transformations co-expressing the GFP are shown in Figure 1C. Two variants of AtCas9, Cas9-HF1 and eCas9 1.1, originally designed to reduce non-specific DNA interactions were also evaluated for mutagenesis of the same target genes. Details of all 16 plasmid constructs used in the study, target genes, and sequences are listed in Supplementary Table S3.

### Co-delivery of GFP With SSN Expression Cassettes to Compare Protoplast Transformation Efficiency Between CRISPR/Cas9 and TALENs Constructs

**FIGURE 2:**
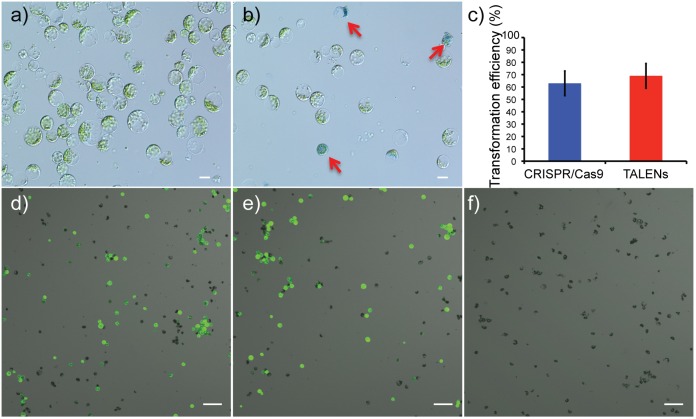
Determination of protoplast viability and transformation efficiency of genome-editing nucleases targeting *ALS1*. **(a)** Protoplasts isolated from *in vitro* grown potato leaves. **(b)** Protoplasts stained with Evans blue to test viability. Arrows indicate defective protoplast cells into which the dye permeated. Scale bar = 20 μ. **(c)** Transformation efficiencies are compared between the CRISPR/Cas9 and TALEN plasmid constructs targeting *ALS1*. Each bar represents the % mean value of three independent transformations, each with five technical replicates ± Standard deviation, Student’s *t*-tests (*P* ≤ 0.05). **(d–f)** Confocal laser scanning microscope images showing, merged images of GFP fluorescence (green) and bright field (gray). CRISPR/Cas9 targeting *ALS1* **(d)**, TALENs targeting *ALS1* **(e)**, and no plasmid control **(f)** are shown. Scale bar = 100 μ.

Viability of protoplasts isolated from leaves of *in vitro* propagated potato was determined using Evans blue staining, a non-permeating dye that is excluded by the cell plasma membrane, indicating cell viability. In contrast, a non-viable or damaged cell stains blue (Figure 2a,b). On average, protoplast viabilities of 90 ± 4% (*n* = 12) were achieved (Supplementary Figures S1A–F). For detection of successful delivery and expression of the genome-editing reagents, GFP was co-delivered on the same plasmid as the Cas9 and TALEN expression cassettes (Figure 1C). Using GFP fluorescence from the Cas9 and TALENs constructs targeting *ALS1*, protoplast transformation efficiency was optimized for cell density and time of exposure to polyethylene glycol (PEG). The transformation efficiency was highest at 100,000 protoplasts cell density per reaction and with a PEG exposure time of 5–10 min but was significantly reduced with PEG exposure times of 15–20 min (*P* ≤ 0.05) for Cas9 construct (Supplementary Figures S1G,H). However, for TALEN construct, efficiency remained constant at these conditions. Protoplast transformation efficiencies of up to 60-65% were achieved using Cas9 and TALEN-based constructs (Figure 2c–f).

### PCR/RE Assay Detects Targeted Mutagenesis of *ALS1* and *EPSPS1* by Disrupting the Restriction Site

**FIGURE 3:**
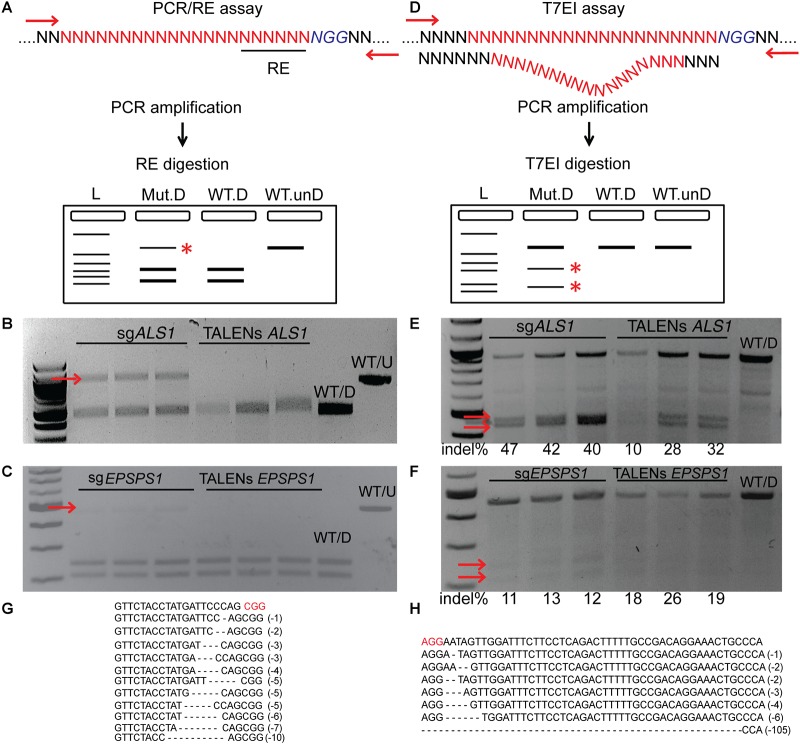
Targeted mutagenesis of *ALS1* and *EPSPS1* detected by PCR/RE and T7EI assays. Three replications of CRISPR/Cas9 and TALENs are used in the assays. **(A)** Schematic of PCR/restriction enzyme digestion assay (PCR/RE) and resulting gel images of **(B)** *ALS1* and **(C)** *EPSPS1* in which the amplicons were digested with *BslI* and *XcmI*, respectively. Mutant bands resistant to digestion in **(B,C)** are indicated by red arrow and were cloned for Sanger sequencing. **(D)** Schematic of T7 Endonuclease I assay (T7EI) and resulting gel images of **(E)** *ALS1* and **(F)** *EPSPS1*. Arrows indicate expected cleavage products from targeted mutagenesis and indel percentage for each sample is indicated below. Sanger sequences of the targeted mutagenesis site of **(G)** *ALS1* and **(H)** *EPSPS1*. ^∗^ Denote the bands present in mutagenized samples only. Protospacer adjacent motif (PAM) is indicated in red. 100 bp NEB ladder; WT/D, wild type digested; WT/U, wild type un-digested.

PCR/RE assays were performed for *ALS1* and *EPSPS1* (Figure 3A). Digestion of *ALS1* and *EPSPS1* amplicons from CRISPR/Cas9 constructs resulted in resistant bands indicating targeted mutagenesis. However, no digestion-resistant bands were obtained for TALENs as the TALEN cleavage sites did not disrupt the RE sites (Figure 3B,C).

### T7EI Assays Detect Targeted Mutagenesis of *ALS1* and *EPSPS1* by Cleavage of Heteroduplexes

T7EI mismatch assays (Figure 3D) for *ALS1* and *EPSPS1* from both Cas9 and TALENs constructs resulted in expected cleavage products indicative of induced insertion or deletion mutations (indels) at the target sites with mutagenesis percentages ranging from 40 to 47% for Cas9 and 10–32% for TALENs targeting *ALS1*, respectively (Figure 3E). For the target gene *EPSPS1*, the bands corresponding to digestion-resistant amplicons (Figure 3C) as well as T7EI assay (Figure 3F) were less intense compared to *ALS1* indicating lower mutagenesis rates ranging from 11 to 26%.

Mutations were confirmed by cloning and sequencing the digestion-resistant bands (Figure 3B,C), revealing deletions ranging from 1–10 bp in *ALS1* and 1–105 bp in *EPSPS1*, 5^′^ of the PAM sequence (Figure 3G,H).

### Quantification of Targeted Mutagenesis at *ALS1* Locus by Amplicon-Sequencing

**FIGURE 4:**
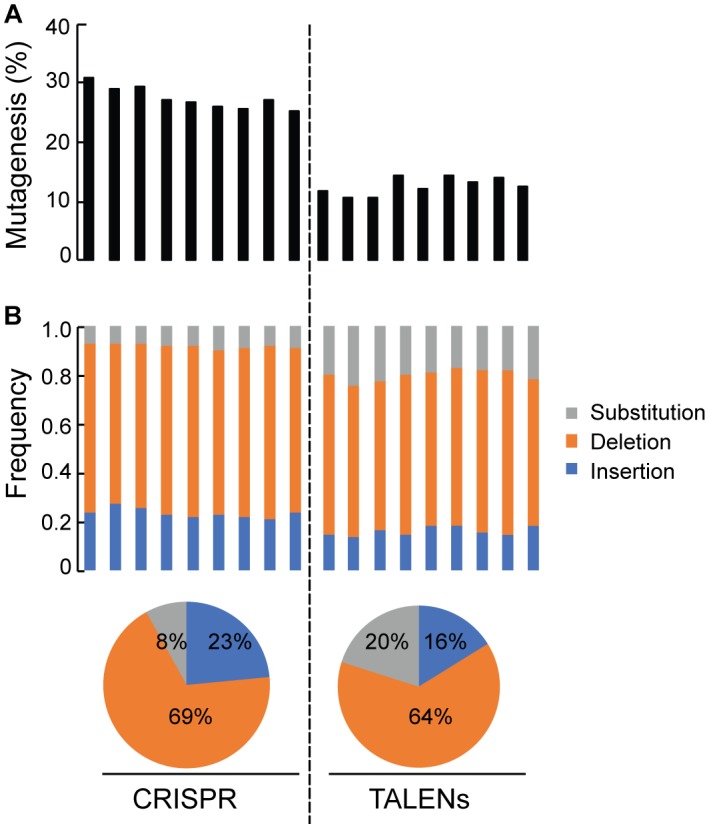
Quantification of targeted mutagenesis at *ALS1* locus by amplicon sequencing. **(A)** Percentage of mutagenized reads at *ALS1* locus are shown using CRISPR/Cas9 and TALENs. The samples represent three biological replicates and three technical replicates of the PCR for each reagent, Student’s *t-*test (*P* ≤ 0.005). **(B)** Frequency of the types of mutations in each sample are shown. Pie charts below represents the total average for each kind of mutation.

Deep sequencing of *ALS1* amplicons generated from CRISPR/Cas9 and TALENs reagents facilitated direct comparison of the efficiency of both reagents for the selected target and quantitatively determined the percentage of targeted mutagenesis along with types of mutations generated (Figure 4). The overall frequency of targeted mutagenesis for *ALS1* was significantly higher with CRISPR/Cas9 (27.4%) compared to the TALENs (12.6%) (*P* ≤ 0.005) (Figure 4A). Nucleotide deletions were the predominant type of mutations using both reagents with the frequencies of deletions and insertions using CRISPR/Cas9 (69, 23%) significantly higher than observed with TALENs (64, 16%), respectively (Figure 4B and Supplementary Figure S2). The frequency of substitutions was significantly lower using CRISPR/Cas9 (8%) than with TALENs (20%) (*P* ≤ 0.005) (Figure 4B and Supplementary Figure S2).

### Detecting CRISPR/Cas9 and TALEN Mediated On-Target Cleavage by Integration of a Blunt-Ended dsODN at the DSB Site in Protoplasts

**FIGURE 5:**
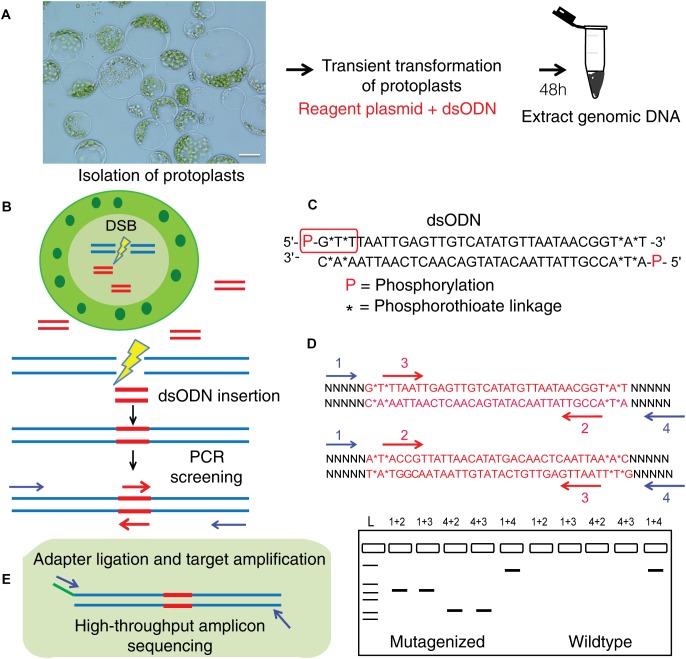
Overview of dsODN integration method for evaluating targeted mutagenesis caused by SSNs in protoplasts. **(A)** Transient transformation of protoplasts with SSN plasmid constructs plus dsODNs (scale bar = 20 μ) and genomic DNA isolation. **(B)** Workflow for detecting SSN mediated on-target cleavage by insertion of a blunt ended dsODN at the double stranded break (DSB) site in protoplast cells and screening for qualitative assessment of functionality. **(C)** The dsODN with 5^′^ phosphorylation and end protection by phosphorothioate linkages at both 5^′^ and 3^′^ ends of both strands shown in red rectangle (Tsai et al., 2015). **(D)** Schematic of dsODN insertion (red) in two possible directions at the target site, primers used for PCR screening along with a resulting gel image are shown. dsODN specific primers (2,3 in red are complementary to each other) and target gene specific primers (1,4 in blue) are used in combinations shown to consider directionality of dsODN integration. The expected results from the PCR reactions are presented. 1+4 reaction is a positive control. **(E)** Schematic of amplicon sequencing of dsODN inserted target sites. dsODN, double stranded oligodeoxynucleotide; SSN, sequence specific nuclease.

**FIGURE 6:**
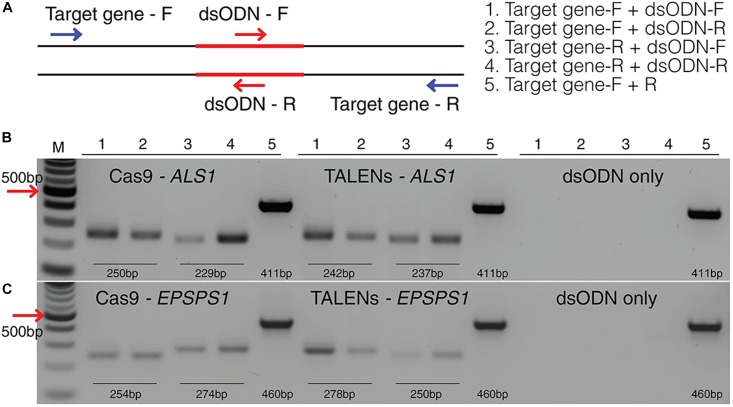
Detection of dsODNs integration in *ALS1* and *EPSPS1* by PCR. **(A)** dsODN insertion is shown at the DSB induced by SSN in target genes and arrows represent the primers used to detect the on-target activity of the dsODN (Supplementary Table S2). The order of PCR reactions for each SSN is shown **(B)** Gel image showing dsODN integration at *ALS1* and **(C)** *EPSPS1*. Target genes have been amplified using dsODN specific primer and gene specific primer. dsODN only is wild type/negative control without nuclease but with dsODNs to account for background DSBs. PCR amplicon sizes assuming one dsODN integration are given. DSB, double stranded break; M, NEB 100 bp ladder.

**FIGURE 7:**
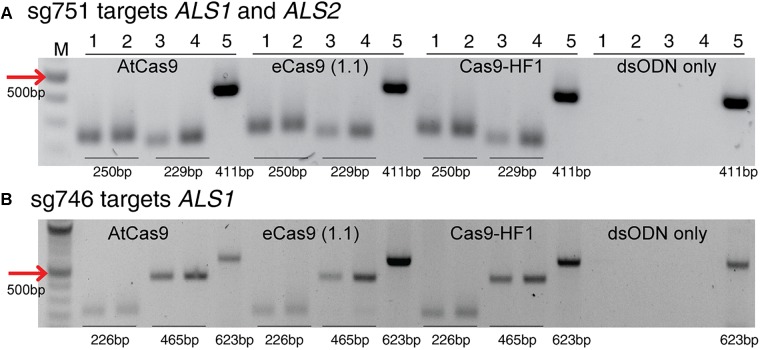
Detection of dsODNs integration by PCR using AtCas9, eCas9 (1.1) and Cas9-HF1 targeting *ALS1* and *ALS2*. Gel images showing dsODN integration at *ALS* locus at the DSB induced by variants of Cas9 including AtCas9, eCas9(1.1) and Cas9-HF1. **(A)** sg751 targets both *ALS1 and ALS2* and **(B)** sg746 is specific for *ALS1* (Supplementary Table S3). The order of PCR reactions for each SSN is according to Figure 6A. dsODN only is wild type/negative control without nuclease but with dsODNs in the protoplast transformation reactions to account for background DSB. PCR amplicon sizes assuming one dsODN integration are given which are same for all Cas9 variants. eCas9(1.1), enhanced specificity Cas9; Cas9-HF1, high fidelity Cas9. M, 100 bp NEB ladder.

This method to evaluate on-target activity of SSNs (Figure 5) relies on integration of blunt dsODNs into the DSBs and subsequent PCR amplification of dsODN-containing target sites. Protoplasts are transformed with plasmid constructs expressing engineered SSNs and dsODNs and genomic DNA is isolated from protoplasts 48 h after transformation (Figure 5A). As SSNs generate DSBs at on- and off-target sites, integration of dsODNs creates a tag of known sequence at the target cleavage site. PCR using one dsODN-specific primer and one target gene specific primer results in detection of on-target cleavage activity of the SSN reagent at the target sites (Figure 5B). dsODNs are modified oligonucleotides which are 5^′^ phosphorylated and end-protected with phosphorothioate linkages that protect them from rapid degradation (Tsai et al., 2015) (Figure 5C). These dsODNs are integrated into genomic DNA at the SSN induced DSBs in either forward 5^′^ or reverse 3^′^ orientation by means of NHEJ. Therefore, the directionality of integration is considered for PCR screening corresponding to four possibilities of on-target dsODN detection (Figure 5D). Adapters are ligated and target is amplified for high throughput sequencing to detect mutagenesis quantitatively (Figure 5E). On-target activity was detected with both CRISPR/Cas9 and TALEN reagents targeting *ALS1* and *EPSPS1* using dsODN integration method by PCR (Figure 6A). The integration of the dsODN occurred using both reagents which generate variable DSBs in both 5^′^ and 3^′^ directions with no orientation bias (Figure 6B,C). As expected, a control with only dsODNs without an SSN did not show any amplification with dsODN specific primers (Figure 6B,C). The dsODN integration method was successfully validated in all 16 SSN constructs with AtCas9, Cas9-HF1, eCas91.1 and TALENs targeting regions of *ALS1*, *ALS2* (Figure 7A,B), *EPSPS1* (Supplementary Figures S3A,B), and *EPSPS2* (Supplementary Figures S4A,B).

### dsODN Insertions at the *ALS1* Locus Suppressed Mutagenesis Frequencies

While PCR amplification of dsODN-target sites provided a qualitative assessment of on-target SSN activity (Figure 6, 7 and Supplementary Figures S3, S4), deep sequencing of *ALS1* amplicons from protoplast transformations with CRISPR/Cas9 and TALENs reagents in the presence of dsODNs not only quantitatively determined the percentage targeted mutagenesis but also dsODN integration frequencies using both reagents targeting *ALS1* (Supplementary Table S4). When dsODNs were included in the protoplast transfection reactions, integration of dsODNs was achieved at the target cleavage site approximately 3 bp upstream of the PAM sequence in case of CRISPR/Cas9 and in the middle of spacer sequence for TALEN reagents. dsODN molecules were integrated into the cleavage site without any directional bias with both reagents. Multiple sequence alignments of mutagenized sequences also show evidence of occasional tandem integrations of up to three dsODNs as well as partial integrations of dsODN at the target cleavage site (Supplementary Figure S5).

Surprisingly, the mutagenesis frequency was substantially suppressed to the extent of no SSN control when dsODNs were added in addition to the CRISPR/Cas9 or TALEN plasmid compared to reactions in which dsODNs were not included (Supplementary Table S4). Moreover, only 0.16 and 0.27% reads, respectively, contained dsODNs insertions in the cleavage site reflective of a small number of gene editing events (Supplementary Table S4). To further investigate if the presence of dsODNs suppressed protoplast transformation efficiency itself, protoplast transfections using CRISPR/Cas9 and TALENs targeting *ALS1* were carried out in the presence and absence of dsODNs and the efficiency of transformation was quantitatively determined by GFP signal (*n* = 3, 5 fields/rep) (Supplementary Figures S6A–O). No significant differences were observed in the transformation efficiencies (*P* ≤ 0.05) (Supplementary Figures S6P,Q) suggesting that dsODNs did not interfere in transformation but instead, inhibited mutagenesis.

## Discussion

Genome-editing represents a new frontier in crop improvement and is a rapidly evolving field with numerous advances increasing both accuracy and precision. While CRISPR/Cas9, TALENs, and other reagents have opened up greater possibilities of genetic manipulation and generation of genetic variability, the most commonly used methods for plant transformation, *Agrobacterium*-mediated transformation or biolistic delivery of transgenes, involve time-consuming tissue culture regeneration procedures. Thus, assessment of genome-editing activity can take several months depending on the species. Therefore, having a versatile cell-based evaluation system that enables large-scale screening of novel technical breakthroughs accelerates the process. Plant protoplasts, very similar to cell cultures in animal systems, serve as an indispensable tool for effective validation and rapid screening of *in vivo* activity of genome-editing SSNs (Lowder et al., 2015; Cermak et al., 2017). Protoplasts isolated from plant tissues retain their cell identity and differentiated state and are amenable to DNA transformation. Protoplast transformations can facilitate direct delivery of DNA to the cell and have short experimental duration with greater transformation efficiency compared to other methods (Jiang et al., 2013; Dlugosz et al., 2016; Baltes et al., 2017). Multi-purpose toolkits developed for plant genome-editing have been validated in protoplasts owing to less time required and the possibility to examine millions of cells at a time. Robotic platforms recently developed for protoplast isolation and transformation further open the possibility of automated high throughput screening of SSNs (Xing et al., 2014; Lowder et al., 2015; Quétier, 2016; Cermak et al., 2017). Co-expressing GFP along with SSN reagents in protoplasts not only ensured detection of the delivery and expression of genome-editing reagents in the cell but also facilitated direct comparison of the transformation efficiencies of CRISPR/Cas9 and TALEN reagents (Figure 2). Protoplasts with GFP signal may also be enriched by fluorescence activated cell-sorting method (FACS) for downstream analyses such as sequencing.

T7EI and PCR/RE assays have been used for validation of genome-editing reagents and detection of mutagenesis (Figure 3A,B). However, these methods have certain drawbacks for mutagenesis screening. For example, PCR/RE assay requires the presence of a restriction site in the target locus that will be disrupted by engineered SSNs. In addition to the absolute requirement of a PAM site in CRISPR systems, a restriction site adds yet another limitation to detect gene editing events and many times, it is difficult to find a suitable restriction site located at the cleavage site for the chosen target. Every target sequence requires a unique restriction enzyme thereby limiting the throughput of this assay. In addition, the restriction site position for the PCR/RE assay also depends upon the reagents used. For example, Cas9 generates a DSB approximately three bases upstream of the PAM sequence whereas TALENs cleave near the center of the spacer sequence. In this study, the PCR/RE assays resulted in yielding a positive signal from CRISPR constructs but not in the case of TALENs targeting *ALS1* and *EPSPS1* (Figure 3C,D). The T7EI assay is another method to detect targeted mutagenesis independent of RE site. However, T7EI suffers from poor sensitivity, background signal and is not cost-effective for large scale screening and most often does not yield conclusive results when protoplast transformation efficiencies are poor (Figure 3G,H). T7EI only cleaves heteroduplex DNA, formed as a result of annealing mutagenized DNA to wild type DNA without mutations. Thus, mutant homoduplexes along with wild type alleles remain intact. In addition, the structure of heteroduplex formed is affected by the target sequence and number of nucleotides that mismatch between the two strands of DNA, ultimately affecting the cleavage efficiency by T7EI (Mashal et al., 1995). T7EI assay is also sensitive to DNA/enzyme ratios and incubation time and is particularly susceptible to false-positive signal from amplification of non-identical paralogs. Deep sequencing of the amplicons represents a highly sensitive method for determining targeted mutagenesis frequencies, however requires high throughput sequencing, additional bio-informatic skills to analyze the data and is not cost effective for using it as an initial screening strategy. Furthermore, large insertions or deletions spanning primer binding sites cannot be detected by this method.

Evaluation of on-target activity of SSNs based on the capture of dsODNs into nuclease-induced DSBs in living cells is an alternative method. Unlike the other existing methods, there is no pre-requisite condition required for this method and it is independent of the type of DSB or context of the cleavage site and works robustly with broad range of reagents (with a blunt/staggered cleavage) using a simple PCR reaction. The workflow of the procedure (Figure 5) is adapted from GUIDE-seq approach, developed in human cells to identify genome-wide off-targets caused by CRISPR/Cas nucleases without having to sequence the whole genome (Tsai et al., 2015). This procedure enables enrichment of those sites with dsODN insertion and sequencing only the adjacent genomic regions, thereby facilitating identification of genome-wide cleavage sites. Here, the GUIDE-seq method has been adapted also for TALENs in addition to CRISPR/Cas9 for the first time in plant protoplasts as a screening methodology to evaluate success of the designed construct for targeted mutagenesis providing a method to identify reagents that lack gene editing activity. By including end-protected 34 nt dsODNs along with the plasmid SSN expression constructs in the protoplast transformation reaction, we are able to evaluate the on-target activity of the SSN. The end modifications on dsODNs protect them from nuclease degradation in the cell, allowing for integration into the genomic DNA at the cleavage site (Tsai et al., 2015). Integration of dsODN occurred in both Cas9-mediated blunt ended DSBs as well as TALEN-mediated staggered DSBs (Figure 6, 7 and Supplementary Figures S3, S4). Similarly, integration of dsODN in Cas12a (Cpf1)-induced staggered cuts has been previously reported in human cells (Kim et al., 2016) thereby making this method applicable to current genome-editing platforms being used. While detection of dsODN integration at a target site by PCR without any high-throughput sequencing provides a rapid qualitative assessment of reagents, it is not a quantitative measure of gene editing and is unable to distinguish mono-allelic from bi-allelic events. The rates of integration of dsODNs were reported to be only two- to threefold lower than the frequencies of indels in human cell gene editing events (Tsai et al., 2015). Yet in plant protoplasts cells, dsODN integration rates were much lower suggesting that the signal we detect for on-target activity evaluation using this method is much lower than the actual mutagenesis itself.

Amplicon sequencing of protoplast DNA treated with dsODNs and nucleases further revealed that mutagenesis rates at the genomic target were drastically reduced (Supplementary Table S4). We hypothesized that the high concentration of dsODN molecules cause cellular toxicity resulting in cell death, thereby excluding dsODN-containing cells within the population from mutagenesis. However, GFP-based quantification of transformation efficiency, an indirect assessment of cell viability, with or without dsODN treatment showed no significant differences in fluorescence detection after 24 h, suggesting that the presence of dsODNs is not cytotoxic (Supplementary Figure S6). An alternative explanation for this reduced mutagenesis frequency is that the presence of a large number of small DNA molecules (dsODNs) interferes with the ability of both Cas9 and TALENs to locate and/or cleave the genomic target, resulting in fewer DSBs. Alternatively, the presence of a large number of small DNA molecules saturates the native exonucleases in the treated cells, resulting in less exonuclease activity on the genomic target. Without exonucleases acting on the genomic DSB, the NHEJ/HR repair machinery repairs the DSB without the formation of indels. We would suggest that the latter explanation is more likely based on the fact that large amounts of DNA amplicons does not affect Cas9 activity (Zeng et al., 2018). Additionally, it has been shown that over-expression of the 3^′^ exonuclease TREX2 increases mutation frequency (Cermak et al., 2017), suggesting that decreased exonuclease activity (due to native exonucleases attempting to degrade the dsODNs) would result in fewer mutations at genomic DSBs.

For PCR detection, relatively few events are required to amplify the ODN integration at the target site and we were able to detect the signal on a gel every time we have used this method. We routinely use the dsODN method in our lab to evaluate the functionality of gene-editing constructs prepared within a 1-week time period by readily detecting on-target activity of reagents (Figure 6, 7 and Supplementary Figures S3, S4), well before proceeding with time-consuming *Agrobacterium*-mediated stable transformation. Annealed dsODNs and primers specific to dsODNs are common to any screen, thereby the only variable being gene specific primers for testing multiple targets. This method, as demonstrated by GUIDE-seq in human cells, may not be optimal for detecting genome-wide off-targets in plant cells without further optimization due to low integration frequencies of dsODNs and suppression of mutagenesis (Supplementary Table S4). However, this method can be used to detect dsODN integration in the *in silico* predicted off-targets. Potential parameters for optimization in protoplasts include, titrating down the dsODN concentrations to empirically find the right amounts of plasmid and dsODNs for each transformation reaction or use dsODNs with decreased phosphorothioate linkage protection, for e.g., only on 3^′^ end of the molecule. As the GUIDE-seq method does not involve whole genome sequencing, when optimized for dsODN integration, it can be broadly used to survey various genome-editing reagents for genome-wide off-targets in various crop species. GUIDE-seq could also identify genomic breakpoint ‘hotspots’ independent of SSNs, thereby has the potential to study meiotic recombination hotspots and chromosomal translocations resulting from joining of on- and off-targets can also be detected using this method (Tsai et al., 2015).

This study compares the existing methods for assessment of *in vivo* activity of engineered genome editing nucleases and also introduces a new strategy adapted from human cells, adding yet another tool to screen for targeted mutagenesis in plant protoplast cells. By high-throughput sequencing, the mutagenesis frequencies as well as types of mutations caused by both CRISPR/Cas9 and TALENs reagents targeting the same gene of interest are compared. Furthermore, our results based on assessment of various screening methodologies indicate that the choice of method is context dependent and initial screening can be achieved in a cost-effective manner in protoplast system. The pros and cons for each strategy are discussed providing guidelines for selection of method for evaluation of targeted mutagenesis.

## Accession Numbers

The amplicon-sequencing data have been deposited in the National Center for Biotechnology Information under BioProject ID (PRJNA491731).

## Author Contributions

SN, DD, CB, CS, and DV conceived the project. SN and CS made the constructs. SN designed and performed all the protoplast experiments, analyses. DK conducted the amplicon sequencing and analyses. TJ performed GFP data analyses and cloning. SN, CS, DK, CB, DV, and DD wrote the manuscript.

## Conflict of Interest Statement

The authors declare that the research was conducted in the absence of any commercial or financial relationships that could be construed as a potential conflict of interest. The reviewer JM-S and handling Editor declared their shared affiliation.

## Footnotes

1 http://www.rgenome.net/

2 https://imagej.nih.gov/ij/

3 https://www.idt.com/

4 http://www.bioinformatics.bbsrc.ac.uk/projects/fastqc

5 www.sas.com

## References

[B1] Altschul S. F. Gish W. Miller W. Myers E. W. Lipman D. J. (1990). Basic local alignment search tool. *J. Mol. Biol.* 215 403–410. 10.1006/jmbi.1990.9999 2231712

[B2] Andersson M. Turesson H. Olsson N. Fält A. S. Olsson P. Gonzalez M. N. (2018). Genome editing in potato via CRISPR-Cas9 ribonucleoprotein delivery. *Physiol. Plant.* 164 378–384. 10.1111/ppl.12731 29572864

[B3] Baerson S. R. (2002). Glyphosate-resistant goosegrass. Identification of a mutation in the target enzyme 5-Enolpyruvylshikimate-3-phosphate synthase. *Plant Physiol.* 129 1265–1275. 10.1104/pp.001560 12114580 PMC166520

[B4] Baltes N. J. Gil-Humanes J. Voytas D. F. (2017). “Genome engineering and agriculture: opportunities and challenges,” in *Gene Editing in Plants Progress in Molecular Biology and Translational Science*, eds Weeks D. P. Yang B. (Cambridge, MA: Academic Press), 1–26. 10.1016/bs.pmbts.2017.03.011 PMC8409219 28712492

[B5] Braatz J. Harloff H.-J. Mascher M. Stein N. Himmelbach A. Jung C. (2017). CRISPR-Cas9 targeted mutagenesis leads to simultaneous modification of different homoeologous gene copies in polyploid oilseed rape (*Brassica napus* L.). *Plant Physiol.* 174 935–942. 10.1104/pp.17.00426 28584067 PMC5462057

[B6] Butler N. M. Atkins P. A. Voytas D. F. Douches D. S. (2015). Generation and inheritance of targeted mutations in potato (*Solanum tuberosum* L.) using the CRISPR/Cas system. *PLoS One* 10:e0144591. 10.1371/journal.pone.0144591 26657719 PMC4684367

[B7] Casini A. Olivieri M. Petris G. Montagna C. Reginato G. Maule G. (2018). A highly specific SpCas9 variant is identified by in vivo screening in yeast. *Nat. Biotechnol.* 36 265–271. 10.1038/nbt.4066 29431739 PMC6066108

[B8] Cermak T. Curtin S. J. Gil-Humanes J. Čegan R. Kono T. J. Y. Konečná E. (2017). A multi-purpose toolkit to enable advanced genome engineering in plants. *Plant Cell* 29 1196–1217. 10.1105/tpc.16.00922 28522548 PMC5502448

[B9] Cermak T. Doyle E. L. Christian M. Wang L. Zhang Y. Schmidt C. (2011). Efficient design and assembly of custom TALEN and other TAL effector-based constructs for DNA targeting. *Nucleic Acids Res.* 39 7879. 10.1093/nar/gkr218 21493687 PMC3130291

[B10] Chen J. S. Dagdas Y. S. Kleinstiver B. P. Welch M. M. Sousa A. A. Harrington L. B. (2017). Enhanced proofreading governs CRISPR-Cas9 targeting accuracy. *Nature* 550 407–410. 10.1038/nature24268 28931002 PMC5918688

[B11] Chen X. Lu X. Shu N. Wang S. Wang J. Wang D. (2017). Targeted mutagenesis in cotton (*Gossypium hirsutum* L.) using the CRISPR/Cas9 system. *Sci. Rep.* 7:44304. 10.1038/srep44304 28287154 PMC5347080

[B12] Cheng J. Veilleux R. E. (1991). Genetic analysis of protoplast culturability in *Solanum phureja*. *Plant Sci.* 75 257–265. 10.1016/0168-9452(91)90241-Y

[B13] Clasen B. M. Stoddard T. J. Luo S. Demorest Z. L. Li J. Cedrone F. (2016). Improving cold storage and processing traits in potato through targeted gene knockout. *Plant Biotechnol. J.* 14 169–176. 10.1111/pbi.12370 25846201 PMC11389148

[B14] Dlugosz E. M. Lenaghan S. C. Stewart C. N. (2016). A robotic platform for high-throughput protoplast isolation and transformation. *J. Vis. Exp.* 115:e54300. 10.3791/54300 27768035 PMC5092064

[B15] Gaff D. F. Okong’o-ogola O. (1971). The use of non-permeating pigments for testing the survival of cells. *J. Exp. Bot.* 22 756–758. 10.1093/jxb/22.3.756

[B16] Gibson D. G. Young L. Chuang R. Y. Venter J. C. Hutchison C. A. Smith H. O. (2009). Enzymatic assembly of DNA molecules up to several hundred kilobases. *Nat. Methods* 6 343–345. 10.1038/nmeth.1318 19363495

[B17] Guschin D. Y. Waite A. J. Katibah G. E. Miller J. C. Holmes M. C. Rebar E. J. (2010). A rapid and general assay for monitoring endogenous gene modification. *Methods Mol. Biol.* 649 247–256. 10.1007/978-1-60761-753-2_15 20680839

[B18] Haun W. Coffman A. Clasen B. M. Demorest Z. L. Lowy A. Ray E. (2014). Improved soybean oil quality by targeted mutagenesis of the fatty acid desaturase 2 gene family. *Plant Biotechnol. J.* 12 934–940. 10.1111/pbi.12201 24851712

[B19] Hsu P. D. Scott D. A. Weinstein J. A. Ran F. A. Konermann S. Agarwala V. (2013). DNA targeting specificity of RNA-guided Cas9 nucleases. *Nat. Biotechnol.* 31 827–832. 10.1038/nbt.2647 23873081 PMC3969858

[B20] Jiang F. Zhu J. Liu H. L. (2013). Protoplasts: a useful research system for plant cell biology, especially dedifferentiation. *Protoplasma* 250 1231–1238. 10.1007/s00709-013-0513-z 23719716

[B21] Kim D. Kim J. Hur J. Been K. W. Yoon S. H. Kim J. S. (2016). Genome-wide target specificities of Cpf1 nucleases in human cells. *Nat. Biotechnol.* 34 1–7. 27272384 10.1038/nbt.3609

[B22] Kim H. Kim S. T. Ryu J. Kang B. C. Kim J. S. Kim S. G. (2017). CRISPR/Cpf1-mediated DNA-free plant genome editing. *Nat. Commun.* 8:14406. 10.1038/ncomms14406 28205546 PMC5316869

[B23] Kleinstiver B. P. Prew M. S. Tsai S. Q. Topkar V. V. Nguyen N. T. Zheng Z. (2015). Engineered CRISPR-Cas9 nucleases with altered PAM specificities. *Nature* 523 481–485. 10.1038/nature14592 26098369 PMC4540238

[B24] Li J. F. Norville J. E. Aach J. McCormack M. Zhang D. Bush J. (2013). Multiplex and homologous recombination-mediated genome editing in Arabidopsis and *Nicotiana benthamiana* using guide RNA and Cas9. *Nat. Biotechnol.* 31 688–691. 10.1038/nbt.2654 23929339 PMC4078740

[B25] Li T. Liu B. Spalding M. H. Weeks D. P. Yang B. (2012). High-efficiency TALEN-based gene editing produces disease-resistant rice. *Nat. Biotechnol.* 30 390–392. 10.1038/nbt.2199 22565958

[B26] Liang Z. Chen K. Li T. Zhang Y. Wang Y. Zhao Q. (2017). Efficient DNA-free genome editing of bread wheat using CRISPR/Cas9 ribonucleoprotein complexes. *Nat. Commun.* 8 1–5. 10.1038/ncomms14261 28098143 PMC5253684

[B27] Lowder L. G. Zhang D. Baltes N. J. Paul J. W. Tang X. Zheng X. (2015). A CRISPR/Cas9 toolbox for multiplexed plant genome editing and transcriptional regulation. *Plant Physiol.* 169 971–985. 10.1104/pp.15.00636 26297141 PMC4587453

[B28] Magoc T. Salzberg S. L. (2011). FLASH: fast length adjustment of short reads to improve genome assemblies. *Bioinformatics* 27 2957–2963. 10.1093/bioinformatics/btr507 21903629 PMC3198573

[B29] Malnoy M. Viola R. Jung M.-H. Koo O.-J. Kim S. Kim J.-S. (2016). DNA-free genetically edited grapevine and apple protoplast using CRISPR/Cas9 ribonucleoproteins. *Front. Plant Sci.* 7:1904. 10.3389/fpls.2016.01904 28066464 PMC5170842

[B30] Martin M. (2011). Cutadapt removes adapter sequences from high-throughput sequencing reads. *EMBnet.J.* 17 10–12. 10.14806/ej.17.1.200

[B31] Mashal R. D. Koontz J. Sklar J. (1995). Detection of mutations by cleavage of DNA heteroduplexes with bacteriophage resolvases. *Nat. Genet.* 9 177–183. 10.1038/ng0295-177 7719346

[B32] Nadakuduti S. S. Starker C. G. Voytas D. F. Buell C. R. Douches D. S. (2019). “Genome editing in potato with CRISPR/Cas9,” in *Plant Genome Editing with CRISPR Systems: Methods and Protocols, Methods in Molecular Biology* Vol. 1917 (Basingstoke: Springer Nature), 183–201. 10.1007/978-1-4939-8991-1_14 30610637

[B33] Ondřej V. Kitner M. Doležalová I. Nádvorník P. Navrátilová B. Lebeda A. (2009). Chromatin structural rearrangement during dedifferentiation of protoplasts of *Cucumis sativus* L. *Mol. Cells* 27 443–447. 10.1007/s10059-009-0057-4 19390825

[B34] Peterson B. A. Holt S. H. Laimbeer F. P. E. Doulis A. G. Coombs J. Douches D. S. (2016). Self-fertility in a cultivated diploid potato population examined with the infinium 8303 potato single-nucleotide polymorphism array. *Plant Genome* 9 1–13. 10.3835/plantgenome2016.01.0003 27902797

[B35] Pinello L. Canver M. C. Hoban M. D. Orkin S. H. Kohn D. B. Bauer D. E. (2016). Analyzing CRISPR genome-editing experiments with CRISPResso. *Nat. Biotechnol.* 34 695–697. 10.1038/nbt.3583 27404874 PMC5242601

[B36] Porebski S. Bailey L. G. Baum B. R. (1997). Modification of a CTAB DNA extraction protocol for plants containing high polysaccharide and polyphenol components. *Plant Mol. Biol. Report.* 15 8–15. 10.1007/BF02772108

[B37] Powles S. B. Preston C. (2006). Evolved glyphosate resistance in plants: biochemical and genetic basis of resistance. *Weed Technol.* 20 282–289. 10.1614/WT-04-142R.1

[B38] Quétier F. (2016). The CRISPR-Cas9 technology: closer to the ultimate toolkit for targeted genome editing. *Plant Sci.* 242 65–76. 10.1016/j.plantsci.2015.09.003 26566825

[B39] Sathasivan K. Haughn G. W. Murai N. (1991). Molecular basis of imidazolinone herbicide resistance in *Arabidopsis thaliana* var columbia. *Plant Physiol.* 97 1044–1050. 10.1104/pp.97.3.1044 16668488 PMC1081121

[B40] Shan Q. Wang Y. Li J. Gao C. (2014). Genome editing in rice and wheat using the CRISPR/Cas system. *Nat. Protoc.* 9 2395–2410. 10.1038/nprot.2014.157 25232936

[B41] Sheen J. (2001). Signal transduction in maize and arabidopsis mesophyll protoplasts. *Plant Physiol.* 127 1466–1475. 10.1104/pp.010820 11743090 PMC1540179

[B42] Shukla V. K. Doyon Y. Miller J. C. Dekelver R. C. Moehle E. A. Worden S. E. (2009). Precise genome modification in the crop species *Zea mays* using zinc-finger nucleases. *Nature* 459 437–441. 10.1038/nature07992 19404259

[B43] Soyk S. Müller N. A. Park S. J. Schmalenbach I. Jiang K. Hayama R. (2017). Variation in the flowering gene self pruning 5G promotes day-neutrality and early yield in tomato. *Nat. Genet.* 49 162–168. 10.1038/ng.3733 27918538

[B44] Sun X. Hu Z. Chen R. Jiang Q. Song G. Zhang H. (2015). Targeted mutagenesis in soybean using the CRISPR-Cas9 system. *Sci. Rep.* 5:10342. 10.1038/srep10342 26022141 PMC4448504

[B45] Tsai S. Q. Zheng Z. Nguyen N. T. Liebers M. Topkar V. V. Thapar V. (2015). GUIDE-seq enables genome-wide profiling of off-target cleavage by CRISPR-Cas nucleases. *Nat. Biotechnol.* 33 187–198. 10.1038/nbt.3117 25513782 PMC4320685

[B46] Waltz E. (2016). Gene-edited CRISPR mushroom escapes US regulation. *Nature* 532:293. 10.1038/nature.2016.19754 27111611

[B47] Wang Y. Cheng X. Shan Q. Zhang Y. Liu J. Gao C. (2014). Simultaneous editing of three homoeoalleles in hexaploid bread wheat confers heritable resistance to powdery mildew. *Nat. Biotechnol.* 32 947–951. 10.1038/nbt.2969 25038773

[B48] Woo J. W. Kim J. Kwon S. I. Corvalán C. Cho S. W. Kim H. (2015). DNA-free genome editing in plants with preassembled CRISPR-Cas9 ribonucleoproteins. *Nat. Biotechnol.* 33 1162–1164. 10.1038/nbt.3389 26479191

[B49] Xiao L. Zhang L. Yang G. Zhu H. He Y. (2012). Transcriptome of protoplasts reprogrammed into stem cells in *Physcomitrella patens*. *PLoS One* 7:e35961. 10.1371/journal.pone.0035961 22545152 PMC3335808

[B50] Xing H. L. Dong L. Wang Z. P. Zhang H. Y. Han C. Y. Liu B. (2014). A CRISPR/Cas9 toolkit for multiplex genome editing in plants. *BMC Plant Biol.* 14:327. 10.1186/s12870-014-0327-y 25432517 PMC4262988

[B51] Xing T. Wang X. (2015). Protoplasts in plant signaling analysis: moving forward in the omics era. *Botany* 93 1–8. 10.1139/cjb-2014-0219

[B52] Yin K. Gao C. Qiu J.-L. (2017). Progress and prospects in plant genome editing. *Nat. Plants* 3:17107. 10.1038/nplants.2017.107 28758991

[B53] Yoo S. D. Cho Y. H. Sheen J. (2007). Arabidopsis mesophyll protoplasts: a versatile cell system for transient gene expression analysis. *Nat. Protoc.* 2 1565–1572. 10.1038/nprot.2007.199 17585298

[B54] Zeng Y. Cui Y. Zhang Y. Zhang Y. Liang M. Chen H. (2018). The initiation, propagation and dynamics of CRISPR-SpyCas9 R-loop complex. *Nucleic Acids Res.* 46 350–361. 10.1093/nar/gkx1117 29145633 PMC5758904

[B55] Zong Y. Wang Y. Li C. Zhang R. Chen K. Ran Y. (2017). Precise base editing in rice, wheat and maize with a Cas9-cytidine deaminase fusion. *Nat. Biotechnol.* 35 438–440. 10.1038/nbt.3811 28244994

