# Supplementary material for: Evaluation of Methods to Assess in vivo Activity of Engineered Genome-Editing Nucleases in Protoplasts
Source: Front Plant Sci. 2019 Feb 8;10:110. doi: 10.3389/fpls.2019.00110 (PMC6376315; doi:10.3389/fpls.2019.00110)
Supplement: Supplementary file 1 [file Data_Sheet_1.PDF]

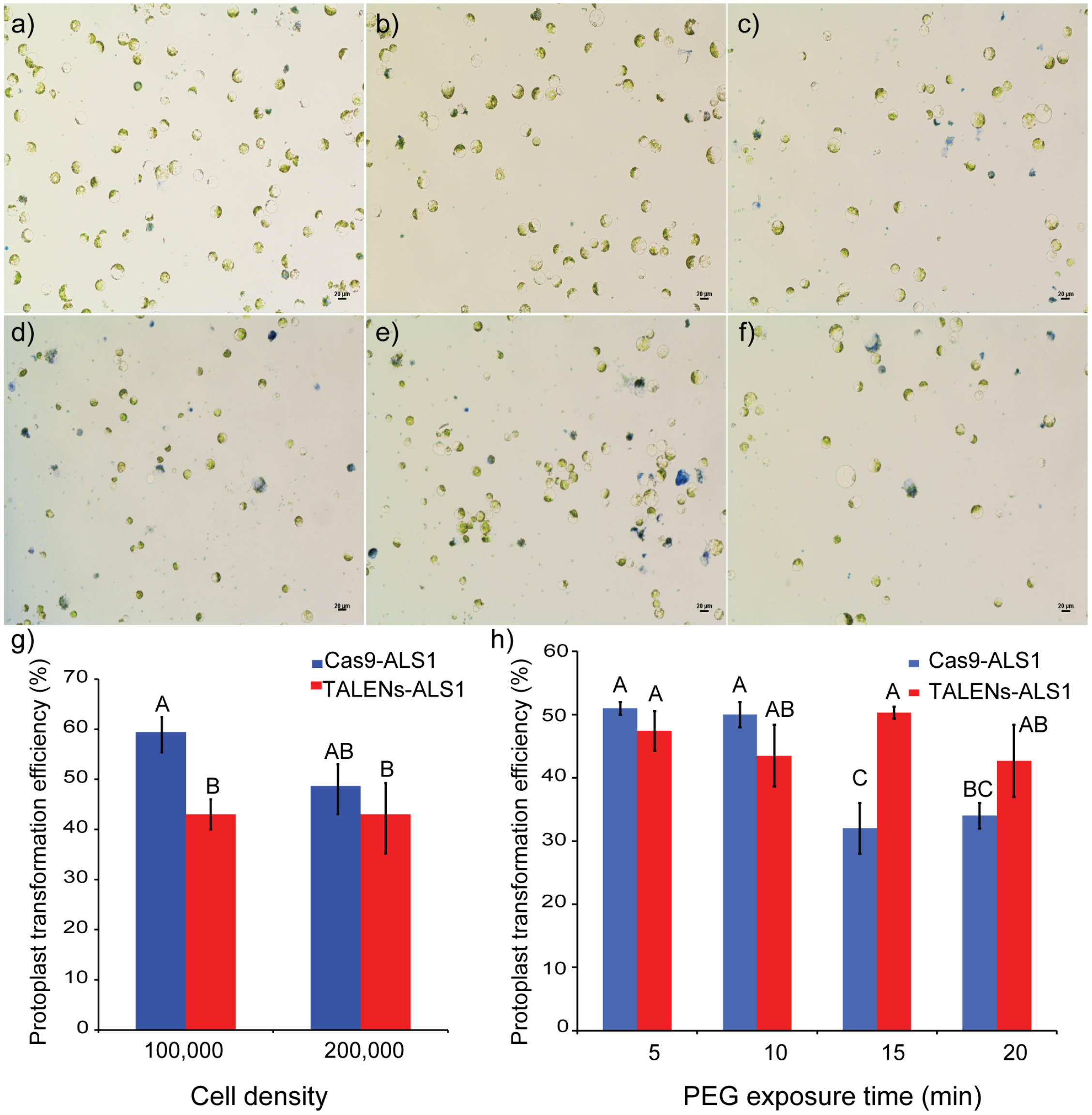

Figure S1. Protoplast viability determination and optimization of transformation efficiency a-f) Protoplast viability determined by counting the number of stained protoplasts relative to the total number of protoplasts. Six independent samples are shown. g) and h) Optimizing the efficiency of protoplast transformation procedures by varying cell density and polyethylene glycol (PEG) exposure time respectively. CRISPR/Cas9 and TALENs plasmid constructs targeting *ALS1* and co-expressing *GFP* were used for the analyses. Each bar represents the % mean value of 3 independent transformations, each with 5 technical replicates ± Standard deviation. Means with different letters are significantly different (Duncan's Multiple Range Test, $P < 0.05$).

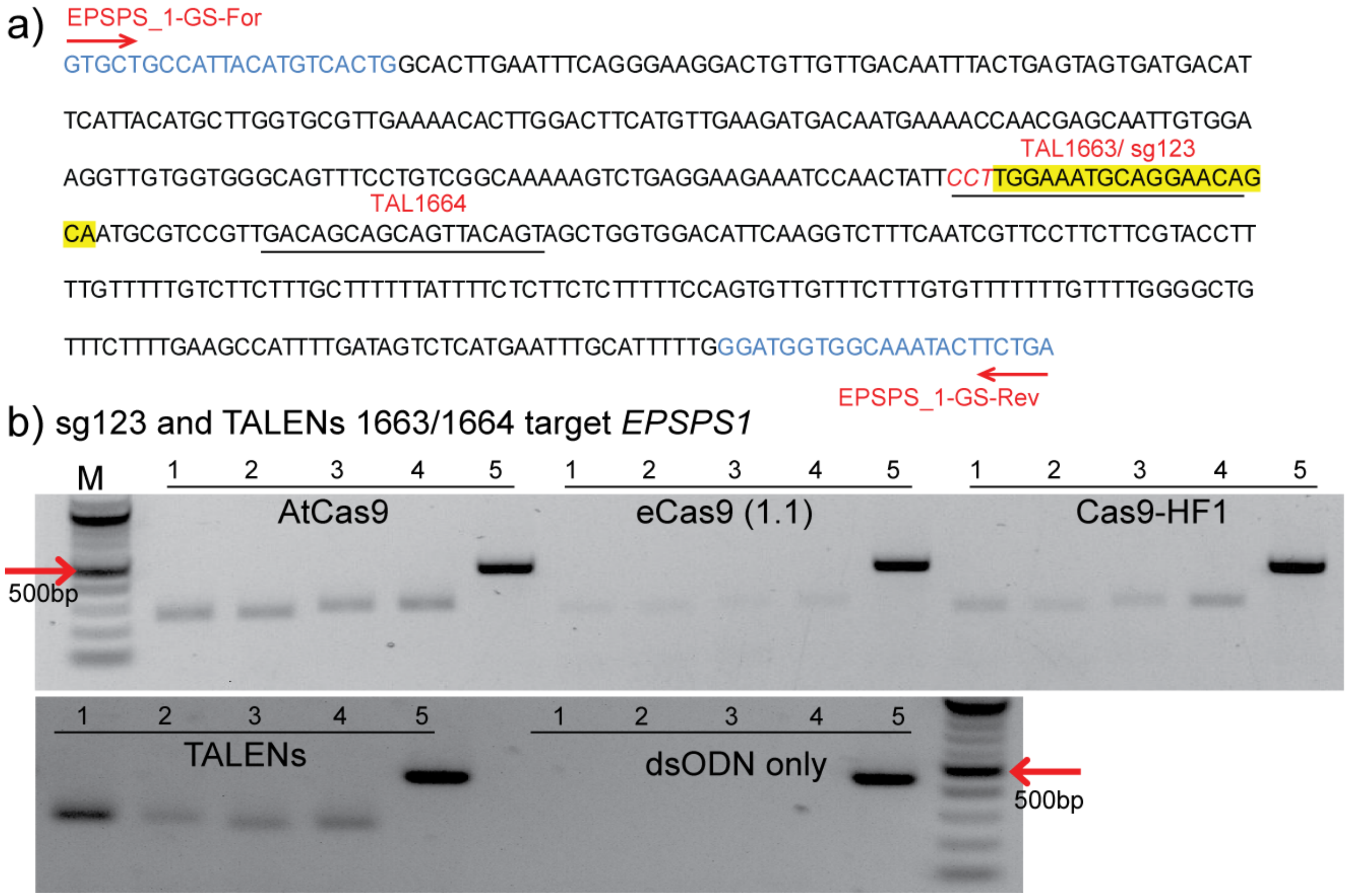

Figure S3. Detection of dsODNs integration by PCR using AtCas9, eCas9 (1.1) and Cas9-HF1 targeting *EPSPS1*. a) *EPSPS1* sequence indicating spacer sequence used to make single guide RNAs (sg123), TALEN binding sites are underlined. Primers used to detect the dsODN integration at the target site by PCR are in blue. b) Gel images showing dsODN integration at *EPSPS1* at the DSB induced by variants of Cas9 including SpCas9, eCas9(1.1) and Cas9-HF1. The order of PCR reactions for each SSN is according to Figure 4a. dsODN only is wild type/negative control without nuclease but with dsODNs in the protoplast transformation reactions to account for background DSB. dsODNs, double stranded oligodeoxynucleotide; *EPSPS1, 5-Enolpyruvylshikimate- 3-phosphate synthase1;* eCas9(1.1), enhanced specificity Cas9; Cas9-HF1, High fidelity Cas9. M = 100bp NEB ladder; DSB = double stranded break. PCR amplicon sizes assuming one dsODN integration are given. Sg123: 1,2 = ~254bp; 3,4=~274bp; 5 = 460bp; TALENs: 1,2 = ~278bp; 3,4=~250bp; 5= 460bp.

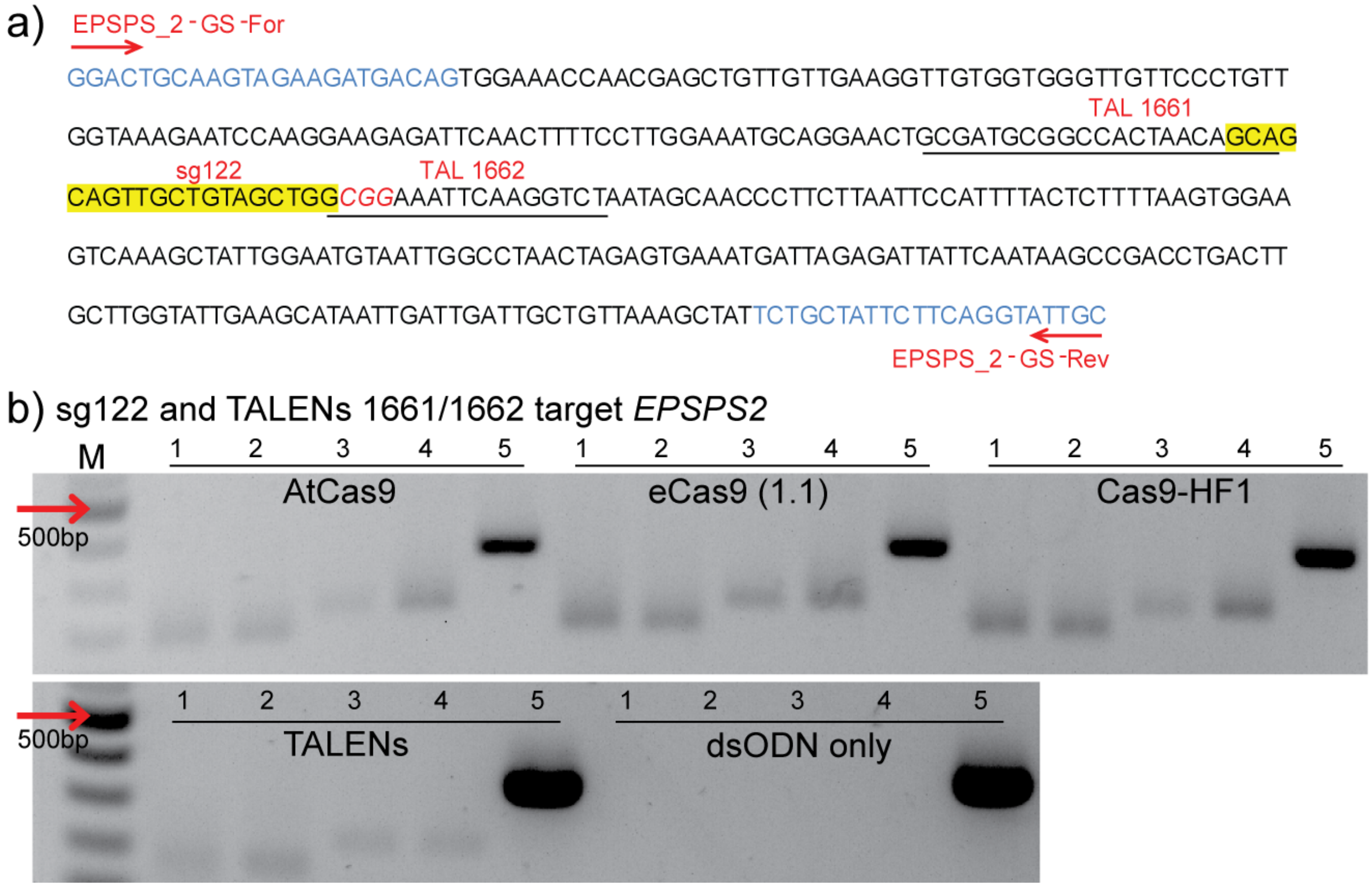

Figure S4. Detection of dsODNs integration by PCR using AtCas9, eCas9 (1.1) and Cas9-HF1 targeting *EPSPS2*. a) *EPSPS2* sequence indicating spacer sequence used to make single guide RNAs (sg122) is highlighted, TALEN binding sites are underlined. Primers used to detect the dsODN integration at the target site by PCR are in blue. b) Gel images showing dsODN integration at *EPSPS2* at the DSB induced by variants of Cas9 including AtCas9, eCas9(1.1) and Cas9-HF1. Target gene has been amplified using dsODN specific primer and gene specific primer. The order of PCR reactions for each SSN is according to Figure 4a. dsODN only is wild type/negative control without nuclease but with dsODNs in the protoplast transformation reactions to account for background DSB. dsODNs, double stranded oligodeoxynucleotide; *EPSPS2, 5-Enolpyruvylshikimate- 3-phosphate synthase2;* eCas9(1.1), enhanced specificity Cas9; Cas9-HF1, High fidelity Cas9. M = 100bp NEB ladder; DSB = double stranded break. PCR amplicon sizes assuming one dsODN integration are given. Sg122: 1,2 = ~192bp; 3,4=~236bp; 5 = 360bp; TALENs: 1,2 = ~187bp; 3,4=~241bp; 5= 360bp.

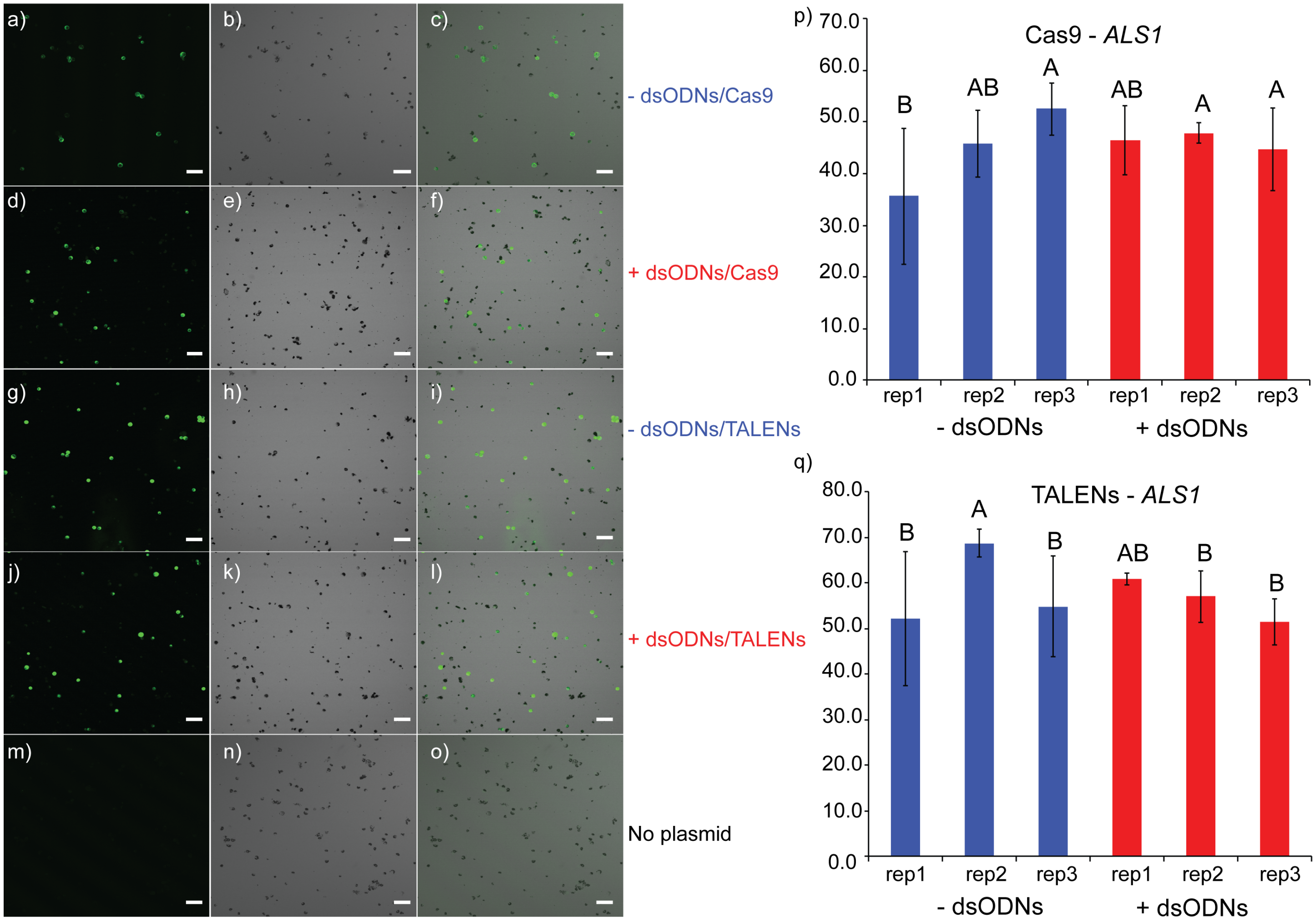

Figure S6. Comparison of transformation efficiency using CRISPR/Cas9 and TALENs targeting *ALS1* in the presence and absence of dsODNs. Confocal laser scanning microscope images 24 hours after protoplast transformation are shown. GFP fluorescence (green), bright field image (grey) and merged images of CRISPR/Cas9 targeting *ALS1* in the absence of dsODNs (a-c) and presence of dsODNS (d-f), TALENs targeting *ALS1* in the absence of dsODNs (g-i) and presence of dsODNS (j-l) and no plasmid control (m-o) are shown. Scale bar = 100µ. p) Transformation efficiencies using CRISPR/Cas9 and q) TALENs in the presence and absence of dsODNs in the transfection reactions are compared. 3 biological replicates per treatment are shown and each bar represents the % mean value of 5 technical replicates ± Standard deviation. Means with different letters are significantly different (Duncan's Multiple Range Test, $P \leq 0.05$).
