# Supplementary material for: Evaluation of Methods to Assess in vivo Activity of Engineered Genome-Editing Nucleases in Protoplasts
Source: Front Plant Sci. 2019 Feb 8;10:110. doi: 10.3389/fpls.2019.00110 (PMC6376315; doi:10.3389/fpls.2019.00110)
Supplement: Supplementary file 2 [file Data_Sheet_2.PDF]

Figure S2. Quantification of targeted mutagenesis of *ALS1* using CRISPR/Cas9 and TALENs in the presence and absence of dsODNs in protoplast transformations. Reads containing mutagenesis are visualized in multiple sequence alignment. To the left of each sequence, the number of reads and its percentage in the total number of reads are provided. The samples represent three biological replicates and three technical replicates of the PCR for each reagent. No reads containing dsODNs were included in this analysis. For CRISPR and TALENs samples, representative reads having greater than 0.5% and 0.1% of total reads respectively are presented. For CRISPR+ODN, TALENs+ODN and only dsODN samples, reads having greater than 0.05% of total reads are presented.

CRISPR 1-1

```
WT              CACGTTCTACCTATGATTCC-CAGCGGCGGTGCTTTCAAAG
9850-68.28      CACGTTCTACCTATGATTCC-CAGCGGCGGTGCTTTCAAAG
603-4.18        CACGTTCTACCTATGA-----CAGCGGCGGTGCTTTCAAAG
603-4.18        CACGTTCTACCTATGATTC--CAGCGGCGGTGCTTTCAAAG
511-3.54        -ACGTTCTACCTATGATTCCCCAGCGGCGGTGCTTTCAAAG
355-2.46        CACGTTCTACCTATG------CAGCGGCGGTGCTTTCAAAG
343-2.37        CACGTTCTACCTATGAC----CAGCGGCGGTGCTTTCAAAG
193-1.33        CACGTTCTACCTATGC-----CAGCGGCGGTGCTTTCAAAG
149-1.03        CACGTTCTACCTATGAT----CAGCGGCGGTGCTTTCAAAG
144-0.99        CACGTTCTACCTATGATT---CAGCGGCGGTGCTTTCAAAG
141-0.97        CACGTTCTACCTATC------CAGCGGCGGTGCTTTCAAAG
125-0.86        CACGTTCTACCTATGATC---CAGCGGCGGTGCTTTCAAAG
122-0.84        CACGTTCTACCTATGATTCCACAGCGGCGGTGCTTTCAAA-
94-0.65         CACGTTCTACCTAT-------CAGCGGCGGTGCTTTCAAAG
79-0.54         CACGTTCTACCTAC-------CAGCGGCGGTGCTTTCAAAG
                 ************        ****************** 
```

CRISPR 1-2

```
WT              CACGTTCTACCTATGATTCC-CAGCGGCGGTGCTTTCAAAG
9782-70.19      CACGTTCTACCTATGATTCC-CAGCGGCGGTGCTTTCAAAG
568-4.07        CACGTTCTACCTATGA-----CAGCGGCGGTGCTTTCAAAG
528-3.78        -ACGTTCTACCTATGATTCCCCAGCGGCGGTGCTTTCAAAG
509-3.65        CACGTTCTACCTATGATTC--CAGCGGCGGTGCTTTCAAAG
373-2.67        CACGTTCTACCTATGAC----CAGCGGCGGTGCTTTCAAAG
279-2.00        CACGTTCTACCTATG------CAGCGGCGGTGCTTTCAAAG
135-0.96        CACGTTCTACCTATGC-----CAGCGGCGGTGCTTTCAAAG
113-0.81        CACGTTCTACCTATGATTCCACAGCGGCGGTGCTTTCAAA-
109-0.78        CACGTTCTACCTATGATT---CAGCGGCGGTGCTTTCAAAG
108-0.77        CACGTTCTACCTATC------CAGCGGCGGTGCTTTCAAAG
107-0.76        CACGTTCTACCTAT-------CAGCGGCGGTGCTTTCAAAG
100-0.71        CACGTTCTACCTATGAT----CAGCGGCGGTGCTTTCAAAG
81-0.58         CACGTTCTACCTATGATC---CAGCGGCGGTGCTTTCAAAG
77-0.55         CACGTTCTACCTATGATTCCTCAGCGGCGGTGCTTTCAAA-
                 *************       ****************** 
```

CRISPR 1-3

```
WT              CACGTTCTACCTATGATTCC-CAGCGGCGGTGCTTTCAAAG
9427-69.90      CACGTTCTACCTATGATTCC-CAGCGGCGGTGCTTTCAAAG
596-4.41        CACGTTCTACCTATGATTC--CAGCGGCGGTGCTTTCAAAG
501-3.71        CACGTTCTACCTATGA-----CAGCGGCGGTGCTTTCAAAG
453-3.35        -ACGTTCTACCTATGATTCCCCAGCGGCGGTGCTTTCAAAG
282-2.09        CACGTTCTACCTATGAC----CAGCGGCGGTGCTTTCAAAG
261-1.93        CACGTTCTACCTATG------CAGCGGCGGTGCTTTCAAAG
173-1.28        CACGTTCTACCTAT-------CAGCGGCGGTGCTTTCAAAG
166-1.23        CACGTTCTACCTATGATT---CAGCGGCGGTGCTTTCAAAG
162-1.20        CACGTTCTACCTATGC-----CAGCGGCGGTGCTTTCAAAG
127-0.94        CACGTTCTACCTATC------CAGCGGCGGTGCTTTCAAAG
124-0.91        CACGTTCTACCTATGATTCCACAGCGGCGGTGCTTTCAAA-
94-0.69         CACGTTCTACCTATGAT----CAGCGGCGGTGCTTTCAAAG
90-0.66         CACGTTCTACCTATGATC---CAGCGGCGGTGCTTTCAAAG
                 *************       ****************** 
```

CRISPR 2-1

```
WT               CACGTTCTACCTATGATTC-CCAGCGGCGGTGCTTTCAAAG
9795-72.34       CACGTTCTACCTATGATTC-CCAGCGGCGGTGCTTTCAAAG
476-3.51         CACGTTCTACCTATGATT--CCAGCGGCGGTGCTTTCAAAG
467-3.44         CACGTTCTACCTATGA-----CAGCGGCGGTGCTTTCAAAG
364-2.68         -ACGTTCTACCTATGATTCCCCAGCGGCGGTGCTTTCAAAG
288-2.12         CACGTTCTACCTATG------CAGCGGCGGTGCTTTCAAAG
280-2.06         CACGTTCTACCTATGAC----CAGCGGCGGTGCTTTCAAAG
205-1.51         CACGTTCTACCTATGC-----CAGCGGCGGTGCTTTCAAAG
160-1.18         CACGTTCTACCTATC------CAGCGGCGGTGCTTTCAAAG
109-0.80         CACGTTCTACCTAT-------CAGCGGCGGTGCTTTCAAAG
99-0.73          CACGTTCTACCTATGAT----CAGCGGCGGTGCTTTCAAAG
84-0.62          CACGTTCTACCTATGATT---CAGCGGCGGTGCTTTCAAAG
82-0.65          CACGTTCTACCTATGATC---CAGCGGCGGTGCTTTCAAAG
                  *************       ********************
```

CRISPR 2-2

```
WT               CACGTTCTACCTATGATTCC-CAGCGGCGGTGCTTTCAAAG
9595-72.71       CACGTTCTACCTATGATTCC-CAGCGGCGGTGCTTTCAAAG
523-3.96         CACGTTCTACCTATGA-----CAGCGGCGGTGCTTTCAAAG
492-3.72         CACGTTCTACCTATGATTC--CAGCGGCGGTGCTTTCAAAG
361-2.73         -ACGTTCTACCTATGATTCCCCAGCGGCGGTGCTTTCAAAG
300-2.27         CACGTTCTACCTATGAC----CAGCGGCGGTGCTTTCAAAG
235-1.78         CACGTTCTACCTATG------CAGCGGCGGTGCTTTCAAAG
139-1.05         CACGTTCTACCTATGATT---CAGCGGCGGTGCTTTCAAAG
123-0.93         CACGTTCTACCTATGC-----CAGCGGCGGTGCTTTCAAAG
115-0.87         CACGTTCTACCTATGATC---CAGCGGCGGTGCTTTCAAAG
107-0.81         CACGTTCTACCTAT-------CAGCGGCGGTGCTTTCAAAG
91-0.68          CACGTTCTACCTATC------CAGCGGCGGTGCTTTCAAAG
80-0.60          CACGTTCTACCTATGATTCCACAGCGGCGGTGCTTTCAAA-
74-0.56          CACGTTCTACCTATGAT----CAGCGGCGGTGCTTTCAAAG
                  *************       *******************
```

CRISPR 2-3

```
WT               CACGTTCTACCTATGATTCCC-AGCGGCGGTGCTTTCAAAG
9607-73.33       CACGTTCTACCTATGATTCCC-AGCGGCGGTGCTTTCAAAG
552-4.21         CACGTTCTACCTATGATTCC--AGCGGCGGTGCTTTCAAAG
443-3.38         CACGTTCTACCTATGAC-----AGCGGCGGTGCTTTCAAAG
353-2.69         -ACGTTCTACCTATGATTCCCCAGCGGCGGTGCTTTCAAAG
261-1.99         CACGTTCTACCTATGC------AGCGGCGGTGCTTTCAAAG
192-1.46         CACGTTCTACCTATGACC----AGCGGCGGTGCTTTCAAAG
111-0.84         CACGTTCTACCTATGATTC---AGCGGCGGTGCTTTCAAAG
107-0.81         CACGTTCTACCTATGCC-----AGCGGCGGTGCTTTCAAAG
101-0.77         CACGTTCTACCTATCC------AGCGGCGGTGCTTTCAAAG
99-0.75          CACGTTCTACCTAT-C------AGCGGCGGTGCTTTCAAAG
87-0.66          CACGTTCTACCTATGATTCCACAGCGGCGGTGCTTTCAAA-
86-0.65          CACGTTCTACCTATGATCC---AGCGGCGGTGCTTTCAAAG
                  *************        ******************
```

CRISPR 3-1

```
WT               CACGTTCTACCTATGATTCC-CAGCGGCGGTGCTTTCAAAG
14088-73.80      CACGTTCTACCTATGATTCC-CAGCGGCGGTGCTTTCAAAG
687-3.59         CACGTTCTACCTATGA-----CAGCGGCGGTGCTTTCAAAG
647-3.38         CACGTTCTACCTATGATTC--CAGCGGCGGTGCTTTCAAAG
506-2.65         -ACGTTCTACCTATGATTCCCCAGCGGCGGTGCTTTCAAAG
395-2.06         CACGTTCTACCTATGAC----CAGCGGCGGTGCTTTCAAAG
340-1.78         CACGTTCTACCTATG------CAGCGGCGGTGCTTTCAAAG
201-1.05         CACGTTCTACCTATGC-----CAGCGGCGGTGCTTTCAAAG
184-0.96         CACGTTCTACCTATGATT---CAGCGGCGGTGCTTTCAAAG
147-0.77         CACGTTCTACCTATC------CAGCGGCGGTGCTTTCAAAG
138-0.72         CACGTTCTACCTATGAT----CAGCGGCGGTGCTTTCAAAG
134-0.70         CACGTTCTACCTAT-------CAGCGGCGGTGCTTTCAAAG
119-0.62         CACGTTCTACCTATGATTCCACAGCGGCGGTGCTTTCAAA-
117-0.61         CACGTTCTACCTATGATC---CAGCGGCGGTGCTTTCAAAG
                  *************       *******************
```

CRISPR 3-2

```
WT               CACGTTCTACCTATGATTCC-CAGCGGCGGTGCTTTCAAAG
13292-72.42      CACGTTCTACCTATGATTCC-CAGCGGCGGTGCTTTCAAAG
709-3.86         CACGTTCTACCTATGA-----CAGCGGCGGTGCTTTCAAAG
638-3.47         CACGTTCTACCTATGATTC--CAGCGGCGGTGCTTTCAAAG
460-2.50         -ACGTTCTACCTATGATTCCCCAGCGGCGGTGCTTTCAAAG
410-2.23         CACGTTCTACCTATG------CAGCGGCGGTGCTTTCAAAG
380-2.07         CACGTTCTACCTATGAC----CAGCGGCGGTGCTTTCAAAG
221-1.20         CACGTTCTACCTATGC-----CAGCGGCGGTGCTTTCAAAG
196-1.06         CACGTTCTACCTATGATT---CAGCGGCGGTGCTTTCAAAG
166-0.90         CACGTTCTACCTATC------CAGCGGCGGTGCTTTCAAAG
144-0.78         CACGTTCTACCTATGAT----CAGCGGCGGTGCTTTCAAAG
128-0.69         CACGTTCTACCTAT-------CAGCGGCGGTGCTTTCAAAG
126-0.68         CACGTTCTACCTATGATC---CAGCGGCGGTGCTTTCAAAG
126a-0.68        CACGTTCTACCTATGATTCCACAGCGGCGGTGCTTTCAAA-
                  *************       *******************
```

CRISPR 3-3

```
WT               CACGTTCTACCTATGATTCCC-AGCGGCGGTGCTTTCAAAG
17942-74.31      CACGTTCTACCTATGATTCCC-AGCGGCGGTGCTTTCAAAG
813-3.36         CACGTTCTACCTATGATTCC--AGCGGCGGTGCTTTCAAAG
805-3.33         CACGTTCTACCTATGAC-----AGCGGCGGTGCTTTCAAAG
630-2.6          -ACGTTCTACCTATGATTCCCCAGCGGCGGTGCTTTCAAAG
433-1.79         CACGTTCTACCTATGC------AGCGGCGGTGCTTTCAAAG
379-1.56         CACGTTCTACCTATGACC----AGCGGCGGTGCTTTCAAAG
294-1.21         CACGTTCTACCTATGCC-----AGCGGCGGTGCTTTCAAAG
242-1.00         CACGTTCTACCTATGATTC---AGCGGCGGTGCTTTCAAAG
222-0.91         CACGTTCTACCTATCC------AGCGGCGGTGCTTTCAAAG
197-0.81         CACGTTCTACCTAT-C------AGCGGCGGTGCTTTCAAAG
178-0.73         CACGTTCTACCTATGATCC---AGCGGCGGTGCTTTCAAAG
158-0.65         CACGTTCTACCTATGATTCCACAGCGGCGGTGCTTTCAAA-
                  *************        ******************
```

CRISPR+ODN 1-1

```
WT               CACGTTCTACCTATGATTCCCAGCGGCGGTGCTTTCAAAG
12115-96.73      CACGTTCTACCTATGATTCCCAGCGGCGGTGCTTTCAAAG
20-0.15          CACGTTCTACCTATGATTCC-AGCGGCGGTGCTTTCAAAG
13-0.10          CACGTTCTGCCTATGATTCCCAGCGGCGGTGCTTTCAAAG
11a-0.08         CACGTTCTACCCATGATTCCCAGCGGCGGTGCTTTCAAAG
11-0.08          CACGTTCTACCTATGATTCCCAGCGGCAGTGCTTTCAAAG
10-0.07          CACGTTCTACCTATGATTCCCAGCGGCGGTTCTTTCAAAG
9-0.07           CACGTTCTACCTATGATTCCCGGCGGCGGTGCTTTCAAAG
9-0.07           CACGTTCTACCTATGATTCCTAGCGGCGGTGCTTTCAAAG
8-0.06           CACGTTCTACCTATGATTCCCAGCGGCGGTACTTTCAAAG
8-0.06           CATATGTTAATAACGGTATCCAGCGGCGGTGCTTTCAAAG
7-0.05           CACGTTCTACCTATGATTCCCAGCGGCGGTGCTTGCAAAG
7-0.05           CACGTTCTACCTATGATTCCCAGCGGCGGTGCGTTCAAAG
7-0.05           CACGTTCTACCTGTGATTCCCAGCGGCGGTGCTTTCAAAG
                 **  *  *      * *  *  ***** ** * * *****
```

CRISPR+ODN 1-2

```
WT               CACGTTCTACCTATGATTCCC-AGCGGCGGTGCTTTCAAAG
10755-97.11      CACGTTCTACCTATGATTCCC-AGCGGCGGTGCTTTCAAAG
21-0.18          -ACGTTCTACCTATGATTCCCCAGCGGCGGTGCTTTCAAAG
21a-0.18         -ATATGACAACTCAATTAAACCAGCGGCGGTGCTTTCAAAG
11-0.09          CACGTTCTACCTATGATTCC-----GGCGGTGCTTTCAAAG
8-0.07           CACGTTCTACCTATGATTCCC--GGCGGCGGTGCTTTCAAAG
7-0.06           CACGTTCTACCTATGATTCCC-AGCGGCTGTGCTTTCAAAG
6-0.05           CACGTTCTACCTATGATTCCC-AGCGGCGGTGCCTTCAAAG
6-0.05           CACGTTCTACCTATGATTCCC-AGCGGCGGTGCTTGCAAAG
6-0.05           CACGTTCTACCTATGATTCCT-AGCGGCGGTGCTTTCAAAG
                  *  *   * **    *        *** **** * *****
```

CRISPR+ODN 1-3

```
WT               CACGTTCTACCTATGATTCCCAGCGGCGGTGCTTTCAAAG
11919-96.92      CACGTTCTACCTATGATTCCCAGCGGCGGTGCTTTCAAAG
```

```
24-0.19          CACGTTCTACCTATGATTC-CAGCGGCGGTGCTTTCAAAG
17-0.13          CACGTTCTACCTATGATTCCCAGCGGCTGTGCTTTCAAAG
12-0.09          CACGTTCTACCTAT-----CCAGCGGCGGTGCTTTCAAAG
10-0.08          CACGTTCTACCTATAATTCCCAGCGGCGGTGCTTTCAAAG
7-0.05           CACGTTCTACCTATGATTCCCTGCGGCGGTGCTTTCAAAG
7-0.05           CACGTTCTACCCATGATTCCCAGCGGCGGTGCTTTCAAAG
7-0.05           CACGTTCTACCTATGATTCTCAGCGGCGGTGCTTTCAAAG
7-0.05           CACGTTCTACCTATGATTCCCAGCGGCGGTTCTTTCAAAG
                 *********** **      * ***** ** *********
```

CRISPR+ODN 2-1

```
WT               -CACGTTCTACCTATGATTCCCAGCGGCGGTGCTTTCAAAG
15047-95.55      -CACGTTCTACCTATGATTCCCAGCGGCGGTGCTTTCAAAG
26-0.16          -CACGTTCTACCTATGATTCC-AGCGGCGGTGCTTTCAAAG
18-0.11          -CACGTTCTACCTATGATTCCCAGCGGCGGTACTTTCAAAG
14-0.08          -CACGTTCTGCCTATGATTCCCAGCGGCGGTGCTTTCAAAG
10-0.06          -CACGTTCTACCTATGATTCCCAGCGGCGGTTCTTTCAAAG
10-0.06          -CACGTTCTACCTATGATTCCCAGCAGCGGTGCTTTCAAAG
9-0.05           -CACGTTCTACCTATGATTCCTAGCGGCGGTGCTTTCAAAG
9-0.05           -CACGTTCTACCTATGATTCCCAGCGGCGGTGCCTTCAAAG
8-0.05           ATATGACAACTCAATTAAACC-AGCGGCGGTGCTTTCAAAG
8-0.05           -CACGTTCTACCTAT------CAGCGGCGGTGCTTTCAAAG
8-0.05           ATATGACAACTCAATTAAACC-AGCGGCGGTGCTTTCAAAG
8-0.05           -CACGTTCTACCTATGATTCCCAGTGGCGGTGCTTTCAAAG
                   * *      * **       **  ***** * *******
```

CRISPR+ODN 2-2

```
WT               -CACGTTCTACCTATGATTCCCAGCGGCGGTGCTTTCAAAG
12439-96.30      -CACGTTCTACCTATGATTCCCAGCGGCGGTGCTTTCAAAG
25-0.19          ATATGACAACTCAATTAAA-CCAGCGGCGGTGCTTTCAAAG
12-0.09          -CACGTTCTACCTATGATT-CCAGCGGCGGTGCTTTCAAAG
10-0.07          -CACGTTCTGCCTATGATTCCCAGCGGCGGTGCTTTCAAAG
10-0.07          -CACGTTCTACCTATGATTCCCGGCGGCGGTGCTTTCAAAG
9-0.06           -CACGTTCTACCTA-------CAGCGGCGGTGCTTTCAAAG
9-0.06           -CACGTTCTACCTATGATTCCCAGCGGCGGTGCGTTCAAAG
8-0.06           -CACGTTCTACCTATGATTTCCAGCGGCGGTGCTTTCAAAG
8-0.06           -CACGTTCTACCTATGATTCCCAGCGGCGGTGCCTTCAAAG
8-0.06           -CACGTTCTACCTATAATTCCCAGCGGCGGTGCTTTCAAAG
8-0.06           -CACGTTCTACCTATGATTCCCAGCGGCAGTGCTTTCAAAG
8-0.06           -CACGTTCTATCTATGATTCCCAGCGGCGGTGCTTTCAAAG
7-0.05           -CACGTTCTACCTATGATTCCCAGCGGCGGTGATTTCAAAG
7-0.05           -CACGTTCTACCTATGATTCCCAGCGGCGGTGCTATCAAAG
                   * *      * *       * ***** ***   ******
```

CRISPR+ODN 2-3

```
WT               CACGTTCTACCTATGATTCCC-AGCGGCGGTGCTTTCAAAG
14639-96.88      CACGTTCTACCTATGATTCCC-AGCGGCGGTGCTTTCAAAG
31-0.20          -ATATGACAACTCAATTAAACCAGCGGCGGTGCTTTCAAAG
15-0.09          CACGTTCTACCTATGATTCC--AGCGGCGGTGCTTTCAAAG
13-0.08          CACGTTCTGCCTATGATTCCC-AGCGGCGGTGCTTTCAAAG
12-0.07          -ACGTTCTACCTATGATTCCCCAGCGGCGGTGCTTTCAAAG
11-0.07          CACGTTCTACCTATGATTCCC-GGCGGCGGTGCTTTCAAAG
9-0.05           CACGTTCTACCTATGATTCCC-AGCGGCGGTGCTTGCAAAG
9-0.05           CACGTTCTACCTATGATTCCC-AGCGGCGATGCTTTCAAAG
8-0.05           CACGTTCTACCTATGATTCCC-AGCGGCGGTACTTTCAAAG
8-0.05           CACGTTCTACCTATGATTCCC-AGCGGCGGTGCCTTCAAAG
                  *  *     **    *      ****** * * * *****
```

CRISPR+ODN 3-1

```
WT               CACGTTCTACCTATGATTCCCAGCGGCGGTGCTTTCAAAG
16038-96.25      CACGTTCTACCTATGATTCCCAGCGGCGGTGCTTTCAAAG
19-0.11          CACGTTCTACCTATGATTCCCAGCGGCGGTGCTTGCAAAG
15-0.09          CACGTTCTACCTATGATTCCCAGCGGCGGTTCTTTCAAAG
14-0.08          CACGTTCTACCTATGATTCCCAGCGGCAGTGCTTTCAAAG
14-0.08          CACGTTCTGCCTATGATTCCCAGCGGCGGTGCTTTCAAAG
12-0.07          CACGTTCTACCTATGATTCCCAGCGGCGGTACTTTCAAAG
10-0.06          CACGTTCTACCTATGATTCCTAGCGGCGGTGCTTTCAAAG
```

```
9-0.05           CACGTTCTACCTATGATTC-----GGCGGTGCTTTCAAAG
9-0.05           CACGTTCTACCTGTGATTCCCAGCGGCGGTGCTTTCAAAG
9-0.05           CACGTTCTACCTATGATTCCCAGCGGCGATGCTTTCAAAG
                 ******** *** ******     ***  * *** *****
```

CRISPR+ODN 3-2

```
WT               CACGTTCTACCTATGATTCCCAGCGGCGGTGCTTTCAAAG
13059-96.85      CACGTTCTACCTATGATTCCCAGCGGCGGTGCTTTCAAAG
12-0.08          CACGTTCTACCTATGATTCCCAGCGGCGGTTCTTTCAAAG
10-0.07          CACGTTCTGCCTATGATTCCCAGCGGCGGTGCTTTCAAAG
10-0.07          CACGTTCTACCTATGATTCCCGGCGGCGGTGCTTTCAAAG
9-0.06           CACGTTCTACCTATGATTCCCAGCGGCGGTGCTTCCAAAG
8-0.05           CACGTTCTACCTATGATTCCCAGCAGCGGTGCTTTCAAAG
8-0.05           CACGTTCTACCTATGATT-CCAGCGGCGGTGCTTTCAAAG
7-0.05           CACGTTCTACCTATGATTCCCTGCGGCGGTGCTTTCAAAG
                 ******** ********* ** ** ***** *** *****
```

CRISPR+ODN 3-3

```
WT               CACGTTCTACCTATGATTCCCAGCGGCGGTGCTTTCAAAG
12171-97.08      CACGTTCTACCTATGATTCCCAGCGGCGGTGCTTTCAAAG
16-0.12          CACGTTCTGCCTATGATTCCCAGCGGCGGTGCTTTCAAAG
10-0.07          CACGTTCTACCTATGATTCCCGGCGGCGGTGCTTTCAAAG
9-0.07           CACGTTCTACCCATGATTCCCAGCGGCGGTGCTTTCAAAG
8-0.06           CACGTTCTACCTATGATTCCCAGCGGCGGTTCTTTCAAAG
8-0.06           CACGTTCTACCTATGATTCCCAGCGGCGGTACTTTCAAAG
8-0.06           CACGTTCTACCTATGATTCCCAGTGGCGGTGCTTTCAAAG
7-0.05           CACGTTCTACCTATAATTCCCAGCGGCGGTGCTTTCAAAG
7-0.05           CACGTTCTACCTGTGATTCCCAGCGGCGGTGCTTTCAAAG
                 ******** **  * ****** * ****** *********
```

TALENs 1-1

```
WT               CACGTTCTACCTATGATTCCCAGCGGCGGTGCTTTCAAAG
12022-87.36      CACGTTCTACCTATGATTCCCAGCGGCGGTGCTTTCAAAG
528-3.83         CACGTTCTACCTA---TTCCCAGCGGCGGTGCTTTCAAAG
174-1.26         CACGTTCTACCTA--ATTCCCAGCGGCGGTGCTTTCAAAG
124-0.90         CACGTTCTACCTT----TCCCAGCGGCGGTGCTTTCAAAG
54-0.39          CACGTTCTACCT-----TCCCAGCGGCGGTGCTTTCAAAG
46-0.33          CACGTTCTACCTG--ATTCCCAGCGGCGGTGCTTTCAAAG
26-0.18          CACGTTCTACCAT----TCCCAGCGGCGGTGCTTTCAAAG
19-0.13          CACGTTCTACCTA----TCCCAGCGGCGGTGCTTTCAAAG
15-0.10          CACGTTCTACCTA--TTTCCCAGCGGCGGTGCTTTCAAAG
14-0.10          CACGTTCTACC------TCCCAGCGGCGGTGCTTTCAAAG
                 ***********      ***********************
```

TALENs 1-2

```
WT               CACGTTCTACCTATGATTCCCAGCGGCGGTGCTTTCAAAG
12586-88.67      CACGTTCTACCTATGATTCCCAGCGGCGGTGCTTTCAAAG
460-3.24         CACGTTCTACCTA---TTCCCAGCGGCGGTGCTTTCAAAG
108-0.76         CACGTTCTACCTA--ATTCCCAGCGGCGGTGCTTTCAAAG
76-0.53          CACGTTCTACCTT----TCCCAGCGGCGGTGCTTTCAAAG
44-0.30          CACGTTCTACCTG--ATTCCCAGCGGCGGTGCTTTCAAAG
36-0.25          CACGTTCTACCAT----TCCCAGCGGCGGTGCTTTCAAAG
30-0.21          CACGTTCTACCT-----TCCCAGCGGCGGTGCTTTCAAAG
29-0.20          CACGTTCTACCTA----TCCCAGCGGCGGTGCTTTCAAAG
23-0.16          CACGTTCTACCTA--TTTCCCAGCGGCGGTGCTTTCAAAG
16-0.11          CACGTTCTACC------TCCCAGCGGCGGTGCTTTCAAAG
                 ***********      ***********************
```

TALENs 1-3

```
WT               CACGTTCTACCTATGATTCCCAGCGGCGGTGCTTTCAAAG
12362-88.45      CACGTTCTACCTATGATTCCCAGCGGCGGTGCTTTCAAAG
435-3.11         CACGTTCTACCTA---TTCCCAGCGGCGGTGCTTTCAAAG
141-1.00         CACGTTCTACCTA--ATTCCCAGCGGCGGTGCTTTCAAAG
116-0.83         CACGTTCTACCT----TTCCCAGCGGCGGTGCTTTCAAAG
33-0.23          CACGTTCTACCT-----TCCCAGCGGCGGTGCTTTCAAAG
28-0.20          CACGTTCTACCTG--ATTCCCAGCGGCGGTGCTTTCAAAG
```

```
27-0.19          CACGTTCTACCA----TTCCCAGCGGCGGTGCTTTCAAAG
19-0.13          CACGTTCTACCT----ATCCCAGCGGCGGTGCTTTCAAAG
18-0.12          CACGTTCTACCTA--TTTCCCAGCGGCGGTGCTTTCAAAG
14-0.10          CACGTTCTACAT-----TCCCAGCGGCGGTGCTTTCAAAG
                 **********       ***********************
```

TALENs 2-1

```
WT              CACGTTCTACCTATGATTCCCAGCGGCGGTGCTTTCAAAG
8586-84.75      CACGTTCTACCTATGATTCCCAGCGGCGGTGCTTTCAAAG
475-4.68        CACGTTCTACCTA---TTCCCAGCGGCGGTGCTTTCAAAG
132-1.30        CACGTTCTACCTA--ATTCCCAGCGGCGGTGCTTTCAAAG
82-0.80         CACGTTCTACCT----TTCCCAGCGGCGGTGCTTTCAAAG
48-0.47         CACGTTCTACCTG--ATTCCCAGCGGCGGTGCTTTCAAAG
28-0.27         CACGTTCTAC-----CTTCCCAGCGGCGGTGCTTTCAAAG
22-0.21         -------CACGTT--CTACCCAGCGGCGGTGCTTTCAAAG
19-0.18         CACGTTCTACCTA--TTTCCCAGCGGCGGTGCTTTCAAAG
16-0.15         -------CACGTT--ATTCCCAGCGGCGGTGCTTTCAAAG
13-0.12         CACGTTCTACCT----ATCCCAGCGGCGGTGCTTTCAAAG
13-0.12         -ACGTTCTACCTAGATTTCCCAGCGGCGGTGCTTTCAAAG
12-0.11         CACGTTCTACCTA-----------GGCGGTGCTTTCAAAG
12-0.11         CACGTTCTACCTATGATTCCCAGCGGCGGTTCTTTCAAAG
11-0.10         CACGTTCTGCCTATGATTCCCAGCGGCGGTGCTTTCAAAG
                         *              ****** *********
```

TALENs 2-2

```
WT              CACGTTCTACCTATGATTCCCAGCGGCGGTGCTTTCAAAG
9307-86.85      CACGTTCTACCTATGATTCCCAGCGGCGGTGCTTTCAAAG
377-3.51        CACGTTCTACCT---ATTCCCAGCGGCGGTGCTTTCAAAG
112-1.04        CACGTTCTACCT--AATTCCCAGCGGCGGTGCTTTCAAAG
102-0.95        CACGTTCTACCT----TTCCCAGCGGCGGTGCTTTCAAAG
69-0.64         CACGTTCTACCT----ATCCCAGCGGCGGTGCTTTCAAAG
42-0.39         CACGTTCTACCT----ATCCCAGCGGCGGTGCTTTCAAAG
33-0.30         CACGTTCTACCT--GATTCCCAGCGGCGGTGCTTTCAAAG
24-0.22         CACGTTCTACCT------CCCAGCGGCGGTGCTTTCAAAG
18-0.16         CACGTTCTACCA----TTCCCAGCGGCGGTGCTTTCAAAG
14-0.13         -ACGTTCTACCT-TATTTCCCAGCGGCGGTGCTTTCAAAG
                 **********       **********************
```

TALENs 2-3

```
WT              CACGTTCTACCTATGATTCCCAGCGGCGGTGCTTTCAAAG
5112-84.80      CACGTTCTACCTATGATTCCCAGCGGCGGTGCTTTCAAAG
305-5.05        CACGTTCTACCT---ATTCCCAGCGGCGGTGCTTTCAAAG
69-1.14         CACGTTCTACCT----TTCCCAGCGGCGGTGCTTTCAAAG
64-1.06         CACGTTCTACCTA--ATTCCCAGCGGCGGTGCTTTCAAAG
30-0.49         CACGTTCTACCTG--ATTCCCAGCGGCGGTGCTTTCAAAG
16-0.26         CACGTTCTACC-----TTCCCAGCGGCGGTGCTTTCAAAG
13-0.21         CACGTTCTACCA----TTCCCAGCGGCGGTGCTTTCAAAG
10-0.16         CACGTTCTA-------TTCCCAGCGGCGGTGCTTTCAAAG
9-0.14          CACGTTCTACC-----TACCCAGCGGCGGTGCTTTCAAAG
9-0.14          CACGTTCTACC------TATGAGCGGCGGTGCTTTCAAAG
8-0.13          CACGTTCTACCTATGATTCCCAGCGGTGGTGCTTTCAAAG
8-0.13          CACGTTCTACC------TCCCAGCGGCGGTGCTTTCAAAG
8-0.13          CACGTTCTA-----------CAGCGGCGGTGCTTTCAAAG
                *********            ***** *************
```

TALENs 3-1

```
WT               CACGTTCTACCTATGATTCCCAGCGGCGGTGCTTTCAAAG
39483-85.96      CACGTTCTACCTATGATTCCCAGCGGCGGTGCTTTCAAAG
1915-4.16        CACGTTCTACCT---ATTCCCAGCGGCGGTGCTTTCAAAG
530-1.15         CACGTTCTACCT--AATTCCCAGCGGCGGTGCTTTCAAAG
475-1.03         CACGTTCTACCT----TTCCCAGCGGCGGTGCTTTCAAAG
179-0.38         CACGTTCTACCT--GATTCCCAGCGGCGGTGCTTTCAAAG
142-0.30         CACGTTCTACCT-----TCCCAGCGGCGGTGCTTTCAAAG
120-0.26         CACGTTCTACCT-----TCCCAGCGGCGGTGCTTTCAAAG
89-0.19          CACGTTCTACCA----TTCCCAGCGGCGGTGCTTTCAAAG
53-0.11          CACGTTCTACCT----ATCCCAGCGGCGGTGCTTTCAAAG
                 ***********      ***********************
```

TALENs 3-2

```
WT               CACGTTCTACCTATGATTCCCAGCGGCGGTGCTTTCAAAG
11661-85.11      CACGTTCTACCTATGATTCCCAGCGGCGGTGCTTTCAAAG
610-4.45         CACGTTCTACCTAT---TCCCAGCGGCGGTGCTTTCAAAG
177-1.29         CACGTTCTACCTA--ATTCCCAGCGGCGGTGCTTTCAAAG
128-0.93         CACGTTCTACCTTT----CCCAGCGGCGGTGCTTTCAAAG
74-0.54          CACGTTCTACCTG--ATTCCCAGCGGCGGTGCTTTCAAAG
72-0.52          CACGTTCTACCTT-----CCCAGCGGCGGTGCTTTCAAAG
39-0.28          CACGTTCTACCATT----CCCAGCGGCGGTGCTTTCAAAG
29-0.21          CACGTTCTACCTAT--TTCCCAGCGGCGGTGCTTTCAAAG
26-0.18          CACGTTCTACCTCC------CAGCGGCGGTGCTTTCAAAG
14-0.10          CACGTTCTACCT--------------CGGTGCTTTCAAAG
                 ***********:              **************
```

TALENs 3-3

```
WT               CACGTTCTACCTATGATTCCCAGCGGCGGTGCTTTCAAAG
9739             CACGTTCTACCTATGATTCCCAGCGGCGGTGCTTTCAAAG
444-3.92         CACGTTCTACC---TATTCCCAGCGGCGGTGCTTTCAAAG
113-1.00         CACGTTCTACCTT----TCCCAGCGGCGGTGCTTTCAAAG
93-0.82          CACGTTCTACCTA--ATTCCCAGCGGCGGTGCTTTCAAAG
37-0.32          CACGTTCTACCTG--ATTCCCAGCGGCGGTGCTTTCAAAG
27-0.24          CACGTTCTACCT-----TCCCAGCGGCGGTGCTTTCAAAG
13-0.11          CACGTTCTACC---GATTCCCAGCGGCGGTGCTTTCAAAG
13-0.11          CACGTTCTA------ATTCCCAGCGGCGGTGCTTTCAAAG
13-0.11          CACGTTCTGCCTATGATTCCCAGCGGCGGTGCTTTCAAAG
13-0.11          CACGTTCTACCAT----TCCCAGCGGCGGTGCTTTCAAAG
13-0.11          CACGTTCTACCTA----TCCCAGCGGCGGTGCTTTCAAAG
12-0.10          CACGTTCTACCTA--TTTCCCAGCGGCGGTGCTTTCAAAG
                 ********         ***********************
```

TALENs+ODN 1-1

```
WT               CACGTTCTACCTATGATTCCCAGCGGCGGTGCTTTCAAAG
8155-96.61       CACGTTCTACCTATGATTCCCAGCGGCGGTGCTTTCAAAG
15-0.17          CACGTTCTACCT---ATTCCCAGCGGCGGTGCTTTCAAAG
6-0.07           CACGTTCTACCTATGATACCCAGCGGCGGTGCTTTCAAAG
6-0.07           CACGTTCTAACTATGATTCCCAGCGGCGGTGCTTTCAAAG
6-0.07           CACGTTCCACCTATGATTCCCAGCGGCGGTGCTTTCAAAG
5-0.05           CACGTTCTACCTATGATTCCCGGCGGCGGTGCTTTCAAAG
5-0.05           CACGTTCTACCTAGGATTCCCAGCGGCGGTGCTTTCAAAG
                 ******* * **   ** *** ******************
```

TALENs+ODN 1-2

```
WT               CACGTTCTACCTATGATTCCCAGCGGCGGTGCTTTCAAAG
7391-96.13       CACGTTCTACCTATGATTCCCAGCGGCGGTGCTTTCAAAG
11-0.14          CACGTTCTGCCTATGATTCCCAGCGGCGGTGCTTTCAAAG
8-0.10           CACGTTCTACCTATGATTCCCAGCGGCGGTGCTTGCAAAG
7-0.09           CACGTTCTACCTA---------GCGGCGGTGCTTTCAAAG
7-0.09           CACGTTCTACCT---ATTCCCAGCGGCGGTGCTTTCAAAG
6-0.07           CACGTTCTACCTATGATTCCCAGCGGCGGTTCTTTCAAAG
6-0.07           CACGTTCTACCTATGATTCCTAGCGGCGGTGCTTTCAAAG
6-0.07           CACGTTCTACCTG--ATTCCCAGCGGCGGTGCTTTCAAAG
5-0.06           CACGTTCTACCTAT-----CCAGCGGCGGTGCTTTCAAAG
5-0.06           CACGTTCTACCTA----------TGGCGGTGCTTTCAAAG
5-0.06           CACGTTCTACCTATGATTCCCGGCGGCGGTGCTTTCAAAG
5-0.06           CACGTTCTACCTATGATTCCCAGCGGCAGTGCTTTCAAAG
4-0.04           CACGTTCTACCTATGATTCCCAGTGGCGGTGCTTTCAAAG
4-0.04           CACGTTCTACCTA--ATTCCCAGCGGCGGTGCTTTCAAAG
4-0.04           CACGTTCTACCTA------------------CTTTCAAAG
4-0.04           CACGTTCTACCTATGATTCCCAGCAGCGGTGCTTTCAAAG
4-0.04           CACGTTCTACCTATAATTCCCAGCGGCGGTGCTTTCAAAG
4-0.04           CACGTTCTAACTATGATTCCCAGCGGCGGTGCTTTCAAAG
4-0.04           CACGTTCTACCTATGATTCCCAGCGGCGGTACTTTCAAAG
4-0.04           CACGTTCTACCTATGATTCCCAGCGGCGGCGCTTTCAAAG
                 ********  **                   *** *****
```

TALENs+ODN 1-3

```
WT               CACGTTCTACCTATGATTCCCAGCGGCGGTGCTTTCAAAG
```

```
6427-96.55      CACGTTCTACCTATGATTCCCAGCGGCGGTGCTTTCAAAG
8-0.12          CACGTTCTACCTATG--TCCCAGCGGCGGTGCTTTCAAAG
5-0.07          CACGTTCTATCTATGATTCCCAGCGGCGGTGCTTTCAAAG
5-0.07          CACGTTCTACCTATGATTCCCAGCGGCGGTGATTTCAAAG
5-0.07          CACGTTCTACCTATGATTCCCAGCGGCAGTGCTTTCAAAG
5-0.07          CACGTTCTACCTAAT--TCCCAGCGGCGGTGCTTTCAAAG
5-0.07          CACGTTCTGCCTATGATTCCCAGCGGCGGTGCTTTCAAAG
4-0.06          CACGTTCTACCTATGATTTCCAGCGGCGGTGCTTTCAAAG
4-0.06          CACGTTCTACCTATGATTCCCAGCAGCGGTGCTTTCAAAG
4-0.06          CACGTTCTACCTATGATTCCCAGCGGCGGTACTTTCAAAG
                ********  ***    * ***** ** **  ********
```

TALENs+ODN 2-1

```
WT              CACGTTCTACCTATGATTCCCAGCGGCGGTGCTTTCAAAG
11811-96.61     CACGTTCTACCTATGATTCCCAGCGGCGGTGCTTTCAAAG
14-0.11         CACGTTCTACCT----TTCCCAGCGGCGGTGCTTTCAAAG
9-0.07          CACGTTCTACCG-------------GCGGTGCTTTCAAAG
9-0.07          CACGTTCTACCTA-----CCCAGCGGCGGTGCTTTCAAAG
9-0.07          CACGTTCTACCT-------------ATGGTGCTTTCAAAG
8-0.06          CACGTTCTGCCTATGATTCCCAGCGGCGGTGCTTTCAAAG
8-0.06          CACGTTCTACCT----ATCCCAGCGGCGGTGCTTTCAAAG
                ******** **                ************* 
```

TALENs+ODN 2-2

```
WT              CACGTTCTACCTATGATTCCCAGCGGCGGTGCTTTCAAAG
11841-97.27     CACGTTCTACCTATGATTCCCAGCGGCGGTGCTTTCAAAG
10-0.08         CACGTTCTACCTAT----CCCAGCGGCGGTGCTTTCAAAG
                **************    **********************
```

TALENs+ODN 2-3

```
WT              CACGTTCTACCTATGATTCCCAGCGGCGGTGCTTTCAAAG
8988-96.89      CACGTTCTACCTATGATTCCCAGCGGCGGTGCTTTCAAAG
11-0.11         CACGTTCTACCTATGATTCCCAGCAGCGGTGCTTTCAAAG
9-0.09          CACGTTCTT--------TCCCAGCGGCGGTGCTTTCAAAG
8-0.08          CACGTTCT----------CCCAGCGGCGGTGCTTTCAAAG
7-0.07          CACGTTCTACCTATGATTCTCAGCGGCGGTGCTTTCAAAG
6-0.06          CACGTTCTACCTATGATTCCTAGCGGCGGTGCTTTCAAAG
6-0.06          CACGTTCTCCCTATGATTCCCAGCGGCGGTGCTTTCAAAG
5-0.05          CACGTTCTACCTATGATTTCCAGCGGCGGTGCTTTCAAAG
5-0.05          CACGTTCTAA------TTCCCAGCGGCGGTGCTTTCAAAG
                ********             *** ***************
```

TALENs+ODN 3-1

```
WT              CACGTTCTACCTATGATTCCCAGCGGCGGTGCTTTCAAAG
13986-96.41     CACGTTCTACCTATGATTCCCAGCGGCGGTGCTTTCAAAG
40-0.27         CACGTTCTACCTA---TTCCCAGCGGCGGTGCTTTCAAAG
15-0.10         CACGTTCTACCCATGATTCCCAGCGGCGGTGCTTTCAAAG
12-0.08         CACGTTCTACCTATGATTCCCAGCGGCGGTTCTTTCAAAG
12-0.08         CACGTTCTACCTATGATTCCCAGCGGCGGTACTTTCAAAG
12-0.08         CACGTTCTGCCTATGATTCCCAGCGGCGGTGCTTTCAAAG
12-0.08         CACGTTCTACCTATGATTCCCGGCGGCGGTGCTTTCAAAG
10-0.06         CACGTTCTACCTATGATTCCCAGTGGCGGTGCTTTCAAAG
10-0.06         CACGTTCTACCTATGATTCCCAGCGGCGGCGCTTTCAAAG
8-0.05          CACGTTCTACCTA--ATTCCCAGCGGCGGTGCTTTCAAAG
8-0.05          CACGTTCTACCTATGATTCCCAGCGACGGTGCTTTCAAAG
8-0.05          CACGTTCCACCTATGATTCCCAGCGGCGGTGCTTTCAAAG
8-0.05          CACGTTCTACCTATGATTCCCAGCGGCAGTGCTTTCAAAG
8-0.05          CACGTTCTACCTATGATTCCCAGCGGCGGTGCTTGCAAAG
8-0.05          ---------CACG---TTCCCAGCGGCGGTGCTTTCAAAG
                         *      ***** * * * *  *** *****
```

TALENs+ODN 3-2

```
WT              CACGTTCTACCTATGATTCCCAGCGGCGGTGCTTTCAAAG
16-0.12         --------------------CAGCGGCGGTGCTTTCAAAG
12-0.09         CACGTTCTACCTATGATTCCCAGCGGTGGTGCTTTCAAAG
12-0.09         CACGTTCTGCCTATGATTCCCAGCGGCGGTGCTTTCAAAG
11-0.08         CACGTTCTACCTATGATTCCCAGCAGCGGTGCTTTCAAAG
```

```
9-0.06          CACGTTCTACCTATGATTCCCAGCGGCGGTGCTTCCAAAG
8-0.06          CACGTTCTACCTATGATTCCCAGCGGCGGTACTTTCAAAG
7-0.05          CACGTTCTACCTATGATTCCCGGCGGCGGTGCTTTCAAAG
7-0.05          CACGTTCTACCTATGATTCCCAGCGGCGGTTCTTTCAAAG
                                    * ** * *** *** *****
```

TALENs+ODN 3-3

```
WT              CACGTTCTACCTATGATTCCCAGCGGCGGTGCTTTCAAAG
13010-97.09     CACGTTCTACCTATGATTCCCAGCGGCGGTGCTTTCAAAG
11-0.08         CACGTTCTACCTATGATTCCCAGCGGTGGTGCTTTCAAAG
9-0.06          CACGTTCTACCTA---TTCCCAGCGGCGGTGCTTTCAAAG
9-0.06          CACGTTCTACCTATGATTCCCAGCGGCGGTTCTTTCAAAG
8-0.05          CACGTTCTGCCTATGATTCCCAGCGGCGGTGCTTTCAAAG
8-0.05          CACGTTCTACCTA--ATTCCCAGCGGCGGTGCTTTCAAAG
7-0.05          CACGTTCTACCTATGATTCCCAGCGGCGGTGCTCTCAAAG
                ******** ****   ********** *** ** ******
```

Only ODN 1-1

```
WT              CACGTTCTACCTATGATTCCCAGCGGCGGTGCTTTCAAAG
12343-97.23     CACGTTCTACCTATGATTCCCAGCGGCGGTGCTTTCAAAG
14-0.11         CACGTTCTGCCTATGATTCCCAGCGGCGGTGCTTTCAAAG
9-0.07          CACGTTCTACCTATGATTCCCAGCGGCGGTTCTTTCAAAG
7-0.05          CACGTTCTACCTATGATTCCCAGCGGCAGTGCTTTCAAAG
7-0.05          CACGTTCTACCTATGATTTCCAGCGGCGGTGCTTTCAAAG
7-0.05          CACGTTCTACCTATAATTCCCAGCGGCGGTGCTTTCAAAG
                ******** ***** *** ******** ** *********
```

Only ODN 1-2

```
WT              CACGTTCTACCTATGATTCCCAGCGGCGGTGCTTTCAAAG
11596-96.98     CACGTTCTACCTATGATTCCCAGCGGCGGTGCTTTCAAAG
16-0.13         CACGTTCTACCTATGATTCCCGGCGGCGGTGCTTTCAAAG
12-0.10         CACGTTCTACCTATGATTCCCAGCGGCGGTTCTTTCAAAG
11-0.09         CACGTTCTACCTATGATTCCCAGCGGCGGTACTTTCAAAG
10-0.08         CACGTTCTACCTATGATTCCCAGCGGCGGTGCTTGCAAAG
7-0.05          CACGTTCTGCCTATGATTCCCAGCGGCGGTGCTTTCAAAG
7-0.05          CACGTTCTACCTATGATTCCCAGCGGCAGTGCTTTCAAAG
7-0.05          CACGTTCTACCTATGATTCCCAGCTGCGGTGCTTTCAAAG
6-0.05          CACGTTCTACCTATGACTCCCAGCGGCGGTGCTTTCAAAG
6-0.05          CACGTTCTACCTATGATTCCCAGTGGCGGTGCTTTCAAAG
6-0.05          CACGTTCTACCTATAATTCCCAGCGGCGGTGCTTTCAAAG
                ******** ***** * **** *  ** ** *** *****
```

Only ODN 1-3

```
WT              CACGTTCTACCTATGATTCCCAGCGGCGGTGCTTTCAAAG
13691-97.17     CACGTTCTACCTATGATTCCCAGCGGCGGTGCTTTCAAAG
14-0.09         CACGTTCTACCTATGATTCCCGGCGGCGGTGCTTTCAAAG
10-0.07         CACGTTCTACCTATGATTCCCAGCGGCGGTTCTTTCAAAG
10-0.07         CACGTTCTGCCTATGATTCCCAGCGGCGGTGCTTTCAAAG
10-0.07         CACGTTCTACCTACGATTCCCAGCGGCGGTGCTTTCAAAG
10-0.07         CACGTTCTACCTATGATTCCCAGCGGCGGTACTTTCAAAG
9-0.06          CACGTTCTACCTATGATTCCCTGCGGCGGTGCTTTCAAAG
8-0.05          CACGTTCTACCTATGATTCCTAGCGGCGGTGCTTTCAAAG
8-0.05          CACGTTCTACCTAGGATTCCCAGCGGCGGTGCTTTCAAAG
                ******** **** ******  ******** *********
```

Only ODN 2-1

```
WT              CACGTTCTACCTATGATTCCCAGCGGCGGTGCTTTCAAAG
19123-97.52     CACGTTCTACCTATGATTCCCAGCGGCGGTGCTTTCAAAG
12-0.06         CACGTTCTGCCTATGATTCCCAGCGGCGGTGCTTTCAAAG
10-0.05         CACGTTCTACCTGTGATTCCCAGCGGCGGTGCTTTCAAAG
10-0.05         CACGTTCTACCTATGATTCCCAGCGGCGGTTCTTTCAAAG
                ******** *** ***************** *********
```

Only ODN 2-2

```
WT              CACGTTCTACCTATGATTCCCAGCGGCGGTGCTTTCAAAG
```

```
16407-96.7      CACGTTCTACCTATGATTCCCAGCGGCGGTGCTTTCAAAG
19-0.11         CACGTTCTGCCTATGATTCCCAGCGGCGGTGCTTTCAAAG
18-0.10         CACGTTCTACCTATGATTCCCAGCGGCGGTACTTTCAAAG
16-0.09         CACGTTCTACCTATGATTCCCAGTGGCGGTGCTTTCAAAG
16-0.09         CACGTTCTACCTATGATTCCCAGCGGCGATGCTTTCAAAG
15-0.08         CACGTTCTACCTATGATTCCCAGCGGCGGTTCTTTCAAAG
13-0.07         CACGTTCTACCTATGATTCCCAGCGGCGGTGCTTGCAAAG
11-0.06         CACGTTCTACCTATGATTCCTAGCGGCGGTGCTTTCAAAG
11-0.06         CACGTTCTACCTATGATTTCCAGCGGCGGTGCTTTCAAAG
11-0.06         CACGTTCTACCTATGATTCCCAGCGGCGGTGCTTCCAAAG
10-0.05         CACGTTCTACCTATGATTCCCGGCGGCGGTGCTTTCAAAG
9-0.05          CACGTTCTACCTATGATTCCCAGCGGCAGTGCTTTCAAAG
9-0.05          CACGTTCTACCTATGATTCCCTGCGGCGGTGCTTTCAAAG
                ******** ********* *  * ***  * *** *****
```

Only ODN 2-3

```
WT               CACGTTCTACCTATGATTCCCAGCGGCGGTGCTTTCAAAG
18777-97.19      CACGTTCTACCTATGATTCCCAGCGGCGGTGCTTTCAAAG
16-0.08          CACGTTCTGCCTATGATTCCCAGCGGCGGTGCTTTCAAAG
15-0.07          CACGTTCTACCTATGATTCCCAGCGGCAGTGCTTTCAAAG
14-0.07          CACGTTCTACCTATGATTCCCAGCGGCGGTTCTTTCAAAG
14-0.07          CACGTTCTACCTATGATTCCTAGCGGCGGTGCTTTCAAAG
12-0.06          CACGTTCTACCTATGATTCCCAGCGGCGGTACTTTCAAAG
11-0.05          CACGTTCTACCTATGATTCCCAGCGGCGGTGATTTCAAAG
11-0.05          CACGTTCTACCTATGATTCCCAGTGGCGGTGCTTTCAAAG
10-0.05          CACGTTCTACCTATGATTCCCAGCGGCGGCGCTTTCAAAG
10-0.05          CACGTTCTACCTATGATTCCCTGCGGCGGTGCTTTCAAAG
                 ******** ***********  * *** *   ********
```

Only ODN 3-1

```
WT               CACGTTCTACCTATGATTCCCAGCGGCGGTGCTTTCAAAG
12013-96.71      CACGTTCTACCTATGATTCCCAGCGGCGGTGCTTTCAAAG
15-0.12          CACGTTCTACCTAT---TCCCAGCGGCGGTGCTTTCAAAG
15-0.12a         CACGTTCTACCTAT-----CCAGCGGCGGTGCTTTCAAAG
14-0.11          CACGTTCTACCCATGATTCCCAGCGGCGGTGCTTTCAAAG
13-0.10          CACGTTCTACCTATGATTCCCAGCGGCAGTGCTTTCAAAG
12-0.09          CACGTTCTGCCTATGATTCCCAGCGGCGGTGCTTTCAAAG
10-0.08          CACGTTCTACCTATGATTCCCAGCGGCGGTGCTTGCAAAG
8-0.06           CACGTTCTACCTATGACTCCCAGCGGCGGTGCTTTCAAAG
8-0.06           CACGTTCTACCTATGATTCCCTGCGGCGGTGCTTTCAAAG
7-0.05           CACGTTCTACCTATGATTCCCAGCGGCGGTTCTTTCAAAG
7-0.05           CACGTTCTACCTATGATTCCCAGCGGCGGTACTTTCAAAG
7-0.05           CACGTTCTACCTATGATTCCCAGCTGCGGTGCTTTCAAAG
7-0.05           CACGTTCTACCTATGATTCCCAGCTGCGGTGCTTTCAAAG
                 ******** ** **     ** ** ** ** *** *****
```

Only ODN 3-2

```
WT               CACGTTCTACCTATGATTCCCAGCGGCGGTGCTTTCAAAG
15618-97.08      CACGTTCTACCTATGATTCCCAGCGGCGGTGCTTTCAAAG
20-0.12          CACGTTCTACCTAT-----CCAGCGGCGGTGCTTTCAAAG
15-0.09          CACGTTCTGCCTATGATTCCCAGCGGCGGTGCTTTCAAAG
14-0.08          CACGTTCTACCTATGATTCCCAGCGGCAGTGCTTTCAAAG
13-0.08          CACGTTCTACCTATGATTCCCAGCAGCGGTGCTTTCAAAG
12-0.07          CACGTTCTACCTATGATTCCCAGCGGCGGTTCTTTCAAAG
11-0.06          CACGTTCTACCTATGATTCCCAGTGGCGGTGCTTTCAAAG
10-0.06          CACGTTCTACCTACGATTCCCAGCGGCGGTGCTTTCAAAG
10-0.06          CACGTTCTACCTATGATTCCCAGCGGCGGTACTTTCAAAG
9-0.05           CACGTTCTACCTATGATTCCCAGCGGCGGTGCTTCCAAAG
                 ******** ****      ****  ** ** *** *****
```

Only ODN 3-3

```
WT               CACGTTCTACCTATGATTCCCAGCGGCGGTGCTTTCAAAG
15440-97.16      CACGTTCTACCTATGATTCCCAGCGGCGGTGCTTTCAAAG
23-0.14          CACGTTCTACCTATGATTCCCAGCGGCAGTGCTTTCAAAG
21-0.13          CACGTTCTGCCTATGATTCCCAGCGGCGGTGCTTTCAAAG
18-0.11          CACGTTCTACCTAT-----CCAGCGGCGGTGCTTTCAAAG
12-0.07          ---------------------CACGGCGGTGCTTTCAAAG
```

```
10-0.06         CACGTTCTACCTATGATTCCTAGCGGCGGTGCTTTCAAAG
8-0.05          CACGTTCTACCTATGATTTCCAGCGGCGGTGCTTTCAAAG
                                       **** ************
```
