# Supplementary material for: Evaluation of Methods to Assess in vivo Activity of Engineered Genome-Editing Nucleases in Protoplasts
Source: Front Plant Sci. 2019 Feb 8;10:110. doi: 10.3389/fpls.2019.00110 (PMC6376315; doi:10.3389/fpls.2019.00110)
Supplement: Supplementary file 3 [file Data_Sheet_3.PDF]

Figure S5. Confirmation of dsODN integration events in the cleavage site of *ALS1* using CRISPR/Cas9 and TALENs by Illumina sequencing. Multiple sequence alignment with all Illumina sequencing reads containing dsODN(s) is presented. The wildtype (WT) sequence is shown at the top. To the left of each sequence, read name is provided. The quantification of reads containing dsODN(s) in all samples was provided in Table S2. The dsODN inserted in forward and reverse orientations are marked by green and red, respectively.

CRISPR+ODN 1-1

```
WT               GCACGTTCTACCTATGATTCC---------------------------------------
Read_1           GCACGTTCTACCTATGATTCCGTTTAATTGAGTTGTCATATGTTAATAACGGTAT-----
Read_2           GCACGTTCTACCTATGATTCCGTTTAATTGAGTTGTCATATGTTAATAACGGTAT-----
Read_3           GCACGTTCTACCTATGATTCCGTTTAATTGAGTTGTCATATGTTAATAACGGTAT-----
Read_4           GCACGTTCTACCTATGATTCCGTTTAATTGAGTTGTCATATGTTAATAACGGTAGTTTAA
Read_5           GCACGTTCTACCTATGATTCCGTTTAATTGAGTTGTCATATGTTAATAACGGTAGTTTAA
Read_6           GCACGTTCTACCTATGATTCCGTTTAATTGAGTTGTCATATGTTAATAACGGTAGTTTAA
Read_7           GCACGTTCTACCTATGATTCCGTTTAATTGAGTTGTCATATGTTAATAACGGTAGTTTAA
Read_8           GCACGTTCTACCTATGAT------------------------------------------
Read_9           GCACGTTCTACCTATGAT------------------------------------------
Read_10          GCACGTTCTACCTATGAT------------------------------------------
Read_11          GCACGTTCTACCTATA--------------------------------------------
Read_12          GCACGTTCTACCTATA--------------------------------------------
Read_13          GCACGTTCTACCTATGATTCCGTTTAATTGAGTTGTCATATGTTAATACCGGTAGTTTAA
Read_14          GCACGTTCTACCTATGATTCCGTTTAATTGAGTTGTCATATGTTAATAACGGTAGGTTAA
Read_15          GCACGTTCTACCTATGATTCCGTTTAATTGAGTTGTCATATGTTAATAACGGTAGTTTAA
Read_16          GCACGTTCTACCTATGATTCCGTTTAATTGAGTTGTCATATGTTAATAACGGTAGTTTAA
Read_17          GCACGTTCTACCTATGATTC-GTTTAATTGAGTTGTCATATGTTAATAACGGTA-TATAC
Read_18          GCACGTTCTACCTATGATTC-GCTTAATTGAATTGTCATATGTTAATAACGGTA-TATAC
Read_19          GCACGTTCTACCTATGATTCCGTTTAATTGAGTTGTCATATGTTAATAACGGTA-TATAC
Read_20          GCACGTTCTACCTATGATTCCGTTTAATTGAGTTGTCATATGTTAATAACGGTA-TATAC
Read_21          GCACGTTCTACCTATGATTC-GTTTAATTGAGTTGTCATATGTTAATAACGGTA-TATAC
                 ***************

Read_1           ATACCGTTATTAACATATGACAACTCAATTAAAC--------------------------
Read_2           ATACCGTTATTAACATATGACAACTCAATTAAAC--------------------------
Read_3           ATACCGTTATTAACATATGACAACTCAATTTAAC--------------------------
Read_4           TTGAGTTGTCATATGTTAATAACGGTATGTTTAATTGAGTTGTCATATGTTAATAACGGT
Read_5           TTGAATTGTCATATGTTAATAACGGTATGTTTAATTGAGTTGTCATATGTTAATAACGGT
Read_6           TTGAGTTGTCATATGTTAATAACGGTATGTTTAATTGAGTTGTCATATGTTAATAACGGT
Read_7           TTGAGTTGTCATATGTTAATAACGGTATGTTTAATTGAGTTGTCATATGTTAATAACGGT
Read_8           -TACCGTTATTAACATATGACAACTCAATTAAAC--------------------------
Read_9           -TACCGTTATTAACATATGACAACTCAATTAAAC--------------------------
Read_10          -TACCGTTATTAACATATGACAACTCAATTAAAC--------------------------
Read_11          -TACCGTTATTAACATATGACAACTCAATTAAAC--------------------------
Read_12          -TACCGTTATTAACATATGACAACTCAATTAAAC--------------------------
Read_13          TTGAGTTGTCATATGTTAATAACGGTATGTTTAATTGAGTTGTCATATGTTAATAACGGT
Read_14          TTGAGTTGTCATATGTTAATAACGGTATGTTTAATTGATTTGTCATATGTTAATAACGGT
Read_15          TTGAGTTGTCATATGTTAATAACGGTATGTTTAATTGAGTTGTCATATGTTAATAACGGT
Read_16          TTGAGTTGTCATATGTTAATAACGGTATGTTTAATTGAGTTGTCATATGTTAATAACGGT
Read_17          GTTATTAACATATGACAACTCAATTAAACATACCGTTATTAACATATGACAACTCAATTA
Read_18          GCTATTATCATATGACAACTCAATTAAACGTACCGTTATTAACATATGACAACTCAATTA
Read_19          GTTATTAACATATGACAACTCAATTAAAC-------------------------------
Read_20          GTTATTAACATATGACAACTCAATTAAAC-------------------------------
Read_21          GTTATTAACATATGACAACTCAATTAAACATACCGTTATTAACATATGACAACTCAATTA

WT               -----CAGCGGCGGTGCTTTCAAAGATGTGATCACAGAGGGTGATGGGAGACGTTCCTAT
Read_1           -----CAGCGGCGGTGCTTTCAAAGATGTGATCACAGAGGGTGATGGGAGACGTTCCTAT
Read_2           -----CAGCGGCGGTGCTTTCAAAGATGTGATCACAGAGGGTGATGGGAGACGTTCCTAT
Read_3           -----CAGCGGCGGTGCTTTCAAAGATGTGATCACAGAGGGTGATGGGAGACGTTCCTAT
Read_4           ATCCAGCGGCGGTGCTTTCAAAGATGTGATCACAGAGGGTGATGGGAGACGTTCCTAT--
Read_5           ATCCAGCGGCGGTGCTTTCAAAGATGTGATCACAGAGGGTGATGGGAGACGTTCCTAT--
Read_6           ATCCAGCGGCGGTGCTTTCAAAGATGTGATCACAGAGGGTGATGGGAGACGTTCCTAT--
Read_7           ATCCAGCGGCGGTGCTTTCAAAGATGTGATCACAGAGGGTGATGGGAGACGTTCCTAT--
Read_8           ----CCAGCGGCGGTGCTTTCAAAGATGTGATCACAGAGGGTGATGGGAGACGTTCCTAT
Read_9           ----CCAGCGGCGGTGCTTTCAAAGATGTGATCACAGAGGGTGATGGGAGACGTTCCTAT
Read_10          ----CCAGCGGCGGTGCTTTCAAAGATGTGATCACAGAGGGTGATGGGAGACGTTCCTAT
```

```
Read_11         GTTTAATTGAGTTGTCATATGTTAATAA--------------------------------
Read_12         GTTTAATTGAGTTGTCATATGTTAATAA--------------------------------
Read_13         AT--CCAGCGGCGGTGCTTTCAAAGATGTGATCACAGAGGGTGATGGGAGACGTTCCTAT
Read_14         AT--CCAGCGGCGGTGCTTTCAAAGATGTGATCACAGAGGGTGATGGGAGACGTTCCTAT
Read_15         AT--CCAGCGGCGGTGCTTTCAAAGATGTGATCACAGAGGGTGATGGGAGACGTTCCTAT
Read_16         AT--CCAGCGGCGGTGCTTTCAAAGATGTGATCACAGAGGGTGATGGGAGACGTTCCTAT
Read_17         AC--CCAGCGGCGGTGCTTTCAAAGATGTGATCACAGAGGGTGATGGGAGACGTTCCTAT
Read_18         TC--CCAGCGGCGGTGCTTTCAAAGATGTGATCACAGAGGGTGATGGGAGACGTTCCTAT
Read_19         -----CAGCGGCGGTGCTTTCAAAGATGTGATCACAGAGGGTGATGGGAGACGTTCCTAT
Read_20         -----CAGCGGCGGTGCTTTCAAAGATGTGATCACAGAGGGTGATGGGAGACGTTCCTAT
Read_21         AAC--CCAGCGGCGGTGCTTTCAAAGATGTGATCACAGAGGGTGATGGGAGACGTTCCTAT
                                *  * ***  * * **        * * **  * * ***  **
```

CRISPR+ODN 1-2

```
WT              CGTTCTACCTATGATTC-------------------------------------------
Read_1          CGTTCTACCTATGATTCCTACCGTTATTAACATATGACAACTCAATTAAAC---------
Read_2          CGTTCTACCTATGATTCATACCGTTATTAACATATGACAACTCAATTAAA----------
Read_3          CGTTCTACCTATGATTCCTACCGTTATTAACATATGACAACTCAATTAAA----------
Read_4          CGTTCTACCTATGATTCCTACCGTTATTAACATATGACAACTCAATTAAACCAGCGGCGG
Read_5          CGTTCTACCTATGATTCCTACCGTTATTAACATATGACAACTCAATTAAAC---------
Read_6          CGTTCTACCTATGATTCCTACCGTTATTAACATATGACAACTCAATTAAAC---------
Read_7          CGTTCTACCTATGATTCCTACCGTTATTAACATATGACAACTCAATTAAAC---------
Read_8          CGTTCTACCTATGATTCATACCGTTATTAACATATGACAACTCAATTAAA----------
Read_9          CGTTCTACCTATGATTCCTACCGTTATTAACATATGACAACTCAATTAAAC---------
Read_10         CGTTCTACCTATGATTCATACCGTTATTAACATATGACAACTCAATTAAA---------
Read_11         CGTTCTACCTATGATTCATACCGTTATTAACATATGACAACTCAATTAAA----------
Read_12         CGTTCTACCTATGATTCATACCGTTATTAACATATGACAACTCAATTAAA----------
Read_13         CGTTCTACCTATGATTCATACCGTTATTAACATATGACAACTCAATTAAA----------
Read_14         CGTTCTACCTATGATTCATACCGTTATTAACATATGACAACTCAATTAAA----------
Read_15         CGTTCTACCTATGATTCATACCGTTATTAACATATGACAACTCAATTAAA----------
Read_16         CGTTCTACCTATGATTCATACCGTTATTAACATATGACAACTCAATTAAAC---------
Read_17         CGTTCTACCTATGATTCATACCGTTATTAACATATGACAACTCAATTAAAC---------
Read_18         CGTTCTACCTATGATTCATACCGTTATTAACATATGACAACTCAATTAAAC---------
Read_19         CGTTCTACCTATGATTCATACCGTTATTAACATATGACAACTCAATTAAAC---------
Read_20         CGTTCTACCTATGATTCCTACCGTTATTAACATATGACAACTCAATTAAAC---------
Read_21         CGTTCTACCTATGATTCCTACCGTTATTAACATATGACAACTCAATTAAAC---------
                *****************

WT              ------------------------------------------------------------
Read_1          ------------------------------------------------------------
Read_2          ------------------------------------------------------------
Read_3          ------------------------------------------------------------
Read_4          ATCTTTCAAAGATGTGATCAAAGAGGGTGATGGGAGACGTTCCGATTGACCTTTAGAAAC
Read_5          ------------------------------------------------------------
Read_6          ------------------------------------------------------------
Read_7          ------------------------------------------------------------
Read_8          ------------------------------------------------------------
Read_9          ------------------------------------------------------------
Read_10         ------------------------------------------------------------
Read_11         ------------------------------------------------------------
Read_12         ------------------------------------------------------------
Read_13         ------------------------------------------------------------
Read_14         ------------------------------------------------------------
Read_15         ------------------------------------------------------------
Read_16         ------------------------------------------------------------
Read_17         ------------------------------------------------------------
Read_18         ------------------------------------------------------------
Read_19         ------------------------------------------------------------
Read_20         ------------------------------------------------------------
Read_21         ------------------------------------------------------------


WT              ------------------------------------------------------------
Read_1          ------------------------------------------------------------
Read_2          --------------------------ATACCGTTATTAACATATGACAACTCAATTAAAC
Read_3          ------------------------------------------------------------
Read_4          TACATACTTGTTGGATGTGATTGTGCCTTATCAGGAGCACGTTCTACCTATGATTCCTAC
Read_5          ------------------------------------------------------------
Read_6          ------------------------------------------------------------
Read_7          ------------------------------------------------------------
```

```
Read_8          --------------------------ATACCGTTATTAACATATGACAACTCAATTAAAC
Read_9          ------------------------------------------------------------
Read_10         --------------------------ATACCGTTATTAACATATGACAACTCAATTAAAC
Read_11         --------------------------ATACCGTTATTAACATATGACAACTCAATTAAAC
Read_12         --------------------------ATACCGTTATTAACATATGACAACTCAATTAAAC
Read_13         --------------------------ATACCGTTATTAACATATGACAACTCAATTAAAC
Read_14         --------------------------ATACCGTTATTAACATATGACAACTCAATTAAAC
Read_15         --------------------------ATACCGTTATTAACATATGACAACTCAATTAAAC
Read_16         ------------------------------------------------------------
Read_17         ------------------------------------------------------------
Read_18         ------------------------------------------------------------
Read_19         ------------------------------------------------------------
Read_20         ------------------------------------------------------------
Read_21         ------------------------------------------------------------

WT              -----------------------------CCAGCGGCGGTGCTTTCAAAGATGTGATCAC
Read_1          ------------------------------CAGCGGCGGTGCTTTCAAAGATGTGATCAC
Read_2          CAGCGGCGGTGCTTTCAAAGATGTGATCAC
Read_3          ------------------------------CAGCGGCGGTGCTTTCAAAGATGTGATCAC
Read_4          CGTTATTAACATATGACAACTCAATTAAACCAGCGGCGGTGCTTTCAAAGATGTGATCAC
Read_5          ------------------------------CAGCGGCGGTGCTTTCAAAGATGTGATCAC
Read_6          ------------------------------CAGCGGCGGTGCTTTCAAAGATGTGATCAC
Read_7          ------------------------------CAGCGGCGGTGCTTTCAAAGATGTGATCAC
Read_8          ------------------------------CAGCGGCGGTGCTTTCAAAGATGTGATCAC
Read_9          ------------------------------CAGCGGCGGTGCTTTCAAAGATGTGATCAC
Read_10         ------------------------------CAGCGGCGGTGCTTTCAAAGATGTGATCAC
Read_11         ------------------------------CAGCGGCGGTGCTTTCAAAGATGTGATCAC
Read_12         ------------------------------CAGCGGCGGTGCTTTCAAAGATGTGATCAC
Read_13         ------------------------------CAGCGGCGGTGCTTTCAAAGATGTGATCAC
Read_14         ------------------------------CAGCGGCGGTGCTTTCAAAGATGTGATCAC
Read_15         ------------------------------CAGCGGCGGTGCTTTCAAAGATGTGATCAC
Read_16         ------------------------------CAGCGGCGGTGCTTTCAAAGATGTGATCAC
Read_17         ------------------------------CAGCGGCGGTGCTTTCAAAGATGTGATCAC
Read_18         ------------------------------CAGCGGCGGTGCTTTCAAAGATGTGATCAC
Read_19         ------------------------------CAGCGGCGGTGCTTTCAAAGATGTGATCAC
Read_20         ------------------------------CAGCGGCGGTGCTTTCAAAGATGTGATCAC
Read_21         ------------------------------CAGCGGCGGTGCTTTCAAAGATGTGATCAC
                                             *******************************
```

CRISPR+ODN 1-3

```
WT              CGTTCTACCTATGATTCC------------------------------------------
Read_1          CGTTCTACCTATGATTCCATACCGTTATTAACATATGACAACTCAATTAAACATACCG--
Read_2          CGTTCTACCTATGATTCCATACCGTTATTAACATATGACAACTCAATTAAACATACCG--
Read_3          CGTTCTACCTATGATTCCGTTTAATTGAGTTGTCATATGTTAATAACGGTATATACCG--
Read_4          CGTTCTACCTATGATTCCCTACCGTTATTAACATATGACAACTCAATTAAACATACCG--
Read_5          CGTTCTACCTATGATTCCGTTTAATTGAGTTGTCATATGTTAATAACGGTATATACCG--
Read_6          CGTTCTACCTATGATTCCATACCGTTATTAACATATGACAACTCAATTAAACATACCG--
Read_7          CGTTCTACCTATGATTCCATACCGTTATTAACATATGCCAACTCAATTAAACATACCG--
Read_8          CGTTCTACCTATGATTCCATACCGTTATTAACATATGACAACTCAATTAAACATACCG--
Read_9          CGTTCTACCTATGATTCCATACCGTTATTAACATATGACAACTCAATTAAACATACCG--
                ******************

WT              ------------------------------------------------------------
Read_1          TTATTAACATATGACAACTCAATTAAAC--------------------------------
Read_2          TTATTAACATATGACAACTCAATTAAAC--------------------------------
Read_3          TTATTAACATATGACAACTCAATTAAACGTTTAATTGAGTTGTCATATGTTAATAACGGT
Read_4          TTATTAACATATGACAACTCAATTAAAC--------------------------------
Read_5          TTATTAACATATGACAACTCAATTAAACGTTTAATTGAGTTGTCATATGTTAATAACGGT
Read_6          TTATTAACATATGACAAGTCAATTAAAC--------------------------------
Read_7          TTATTAACATATGACAACTCAATTAAAC--------------------------------
Read_8          TTATTAACATATGACAACTCAATTAAAC--------------------------------
Read_9          TTATTAACATATGACAACTCAATTAAAC--------------------------------

WT              --CAGCGGCGGTGCTTTCAAAGATGTGATCACAGAGGGTGATGGGAGACGTTCCTATTGA
Read_1          --CAGCGGCGGTGCTTTCAAAGATGTGATCACAGAGGGTGATGGGAGACGTTCCTATTGA
Read_2          --CAGCGGCGGTGCTTTCAAAGATGTGATCACAGAGGGTGATGGGAGACGTTCCTATTGA
Read_3          ATCAGCGGCGGTGCTTTCAAAGATGTGATCACAGAGGGTGATGGGAGACGTTCCTATTGA
Read_4          --CAGCGGCGGTGCTTTCAAAGATGTGATCACAGAGGGTGATGGGAGACGTTCCTATTGA
```

```
Read_5          ATCAGCGGCGGTGCTTTCAAAGATGTGATCACAGAGGGTGATGGGAGACGTTCCTATTGA
Read_6          --CAGCGGCGGTGCTTTCAAAGATGTGATCACAGAGGGTGATGGGAGTCGTTCCTATTGT
Read_7          --CAGCGGCGGTGCTTTCAAAGATGTGATCACGGGGGGTGATGGGAGACGTTACTATTAA
Read_8          --CAGCGGCGGTACTTTAAAAGATGTGATCACAGAGGGTGAGGGGAGACGTTTATATTGA
Read_9          --CAGCGGCGGTGCTTTCAAAGATGTGATCACAGAGGGTGATGGGAGACGTTCCTATTGA
                  ********** **** ************** * ****** ***** ****  ****
```

CRISPR+ODN 2-1

```
WT              CGTTCTACCTATGATTCC------------------------------------------
Read_1          CGTTCTACCTATGATTCCATACCGTTATTAACATATGACAACTCAATTAAACCAGCGG
Read_2          CGTTCTACCTATGA----TTC---------------------------------------
Read_3          CGTTCTACCTATGA----TTC---------------------------------------
Read_4          CGTTCTACCTATGATTCCATACCGTTATTAACATATGACAACTCAATTAAAC--------
Read_5          CGTTCTACCTATGATTCC------------------------------------------
Read_6          CGTTCTACCTATGATTCC------------------------------------------
Read_7          CGTTCTACCTATGATTCCGTTTAATTGAGTTGTCATATGTTAATAACGGTATCAGCGGCG
Read_8          CGTTCTACCTATGATTCCGTTTAATTGAGTTGTCATATGTTAATAACGGTAT--------
Read_9          CGTTCTACCT--------ATACCGTTATTAACATATGACAACTCAATTAAAC--------
Read_10         CGTTCTACCT--------ATACCGTTATTAACATATGACAACTCAATTAAAC--------
Read_11         CGTTCTACCTATGATTCCGTTTAATTGAGTTGTCATATGTTAATAACGGTAT--------
Read_12         CGTTCTACCTATGATTCCGTTTAATTGAGTTGTCATATGTTAATAACGGTAT--------
Read_13         CGTTCTACCTATGATTCCGTTTAATTGAGTTGTCATATGTTAATACCGGTAT--------
Read_14         CGTTCTACCTATGATTCCGTTTAATTGAGTTGTCATATGTTAATAACGGTAT--------
Read_15         CGTTCTACCTATGATTCCATACCGTTATTAACATATGACAACTCAATTAAAC--------
Read_16         CGTTCTACCTATGATTCCATACCGTTATTAACATATGACAACTCAATTAAAC--------
Read_17         CGTTCTACCTATGATTCCATACCGTTATTAACATATGACAACTCAATTAAAC--------
Read_18         CGTTCTACCTATGATTCCGTTTAATTGAGTTGTCATATGTTAATACCGGTAT--------
Read_19         CGTTCTACCTATGATTCCGTTTAATTGAGTTGTCATATGTTAATAACGGTAT--------
Read_20         CGTTCTACCTATGATTC-------------------------------------------
Read_21         CGTTCTACCTATTA----ATACCGTTATTAACATATGACAACTCAATTAAAC--------
Read_22         CGTTCTACCTATGA----ATACCGTTATTAACATATGACAACTCAATTAAAC--------
Read_23         CGTTCTACCTATGAGTCCATACAGTTATTAACATATGACAACTCAATTAAAC--------
Read_24         CGTTCTACCTATGATTCCATACCGTTATTAACATATGACAACTCAATTAAAC--------
Read_25         CGTTCTACCTATGATTCCATACCGTTATTAACATATGACAACTCAATTAAAC--------
Read_26         CGTTCTACCTATGATTCCATACCGTTATTAACATATGACAACTCAATTAAAC--------
Read_27         CGTTCTACCTAT------GTTTAATTGAGTTGTCATATGTTAATAACGGTAT--------
Read_28         CGTTCTACCTAT------GTTTAATTGAGTTGTCATATGTTAATAACGGTAT--------
Read_29         CGTTCTACCTAA------------------------------------------------
Read_30         CGTTCTACCTATGATTCCATACCGTTATTAACATATGACAACTCAATTAAAC--------
Read_31         CGTTCTACCTATGATTCCATACCGTTATTAACATATGACAACTCAATTAAAC--------
Read_32         CGTTCTACCTAT------GTTTAATTGAGTTGTCATATGTTAATAACGGTAT--------
Read_33         CGTTCTACCTATG-----------------------------------------------
Read_34         CGTTCTACCTATG-----------------------------------------------
Read_35         CGTTCTACCTATG-----------------------------------------------
Read_36         ------------------------------------------------------------
Read_37         ------------------------------------------------------------
Read_38         CGTTCTACCTATGATTCCGTTTAATTGAGTTGTCATATGTTAATAACGGTAT--------
Read_39         CGTTCTACCTATGATTCCGTTTAATTGAGTTGTCATATGTTAATAACGGTAT--------
Read_40         CGTTCTACCTATGATTCCGTTTAATTGAGTTGTCATATGTTAATAACGGTAT--------


WT              ------------------------------------------------------------
Read_1          CGGTGCTTCCAAAGATGTGATCCCCGAGGGTGATGGGAGACGTTCCTATTGACTTTTAGA
Read_2          ------------------------------------------------------------
Read_3          ------------------------------------------------------------
Read_4          ------------------------------------------------------------
Read_5          ------------------------------------------------------------
Read_6          ------------------------------------------------------------
Read_7          --GTGCTTTCAAAGATGTGATCCCAGAGGGTGATGGGAGACGTTACTATTGACTTTTAGA
Read_8          ------------------------------------------------------------
Read_9          ------------------------------------------------------------
Read_10         ------------------------------------------------------------
Read_11         ------------------------------------------------------------
Read_12         ------------------------------------------------------------
Read_13         ------------------------------------------------------------
Read_14         ------------------------------------------------------------
Read_15         ------------------------------------------------------------
Read_16         ------------------------------------------------------------
Read_17         ------------------------------------------------------------
Read_18         ------------------------------------------------------------
```

```
Read_19          ------------------------------------------------------------
Read_20          ------------------------------------------------------------
Read_21          ------------ATACCGTTATTAACATATGACAACTCAATTAAAC--------------
Read_22          -----------ATACCGTTATTAACATATGACAACTCAATTAAAC
Read_23          ------------------------------------------------------------
Read_24          ------------------------------------------------------------
Read_25          ------------------------------------------------------------
Read_26          ------------------------------------------------------------
Read_27          -------------ACCGTTATTAACATATGACAACTCAATTAAAC---------------
Read_28          -------------ACCGTTATTAACATATGACAACTCAATTAAAC---------------
Read_29          ------------------------------------------------------------
Read_30          ------------------------------------------------------------
Read_31          ------------------------------------------------------------
Read_32          -------------ACCGTTATTAACATATGACAACTCAATTAAAC---------------
Read_33          ------------------------------------------------------------
Read_34          ------------------------------------------------------------
Read_35          ------------------------------------------------------------
Read_36          ------------------------------------------------------------
Read_37          ------------------------------------------------------------
Read_38          ------------------------------------------------------------
Read_39          ------------------------------------------------------------
Read_40          ------------------------------------------------------------


WT               ------------------------------------------------------------
Read_1           AACTACATACTTGTTGGATGTGATTGTACCTCATCAGGAGCACGTTCTACCTATGATTCC
Read_2           -----------------------------------------------------------C
Read_3           -----------------------------------------------------------C
Read_4           -----------------------------------------------------------C
Read_5           -----------------------------------------------------------C
Read_6           -----------------------------------------------------------C
Read_7           AACTACATACTTGTTGGATGTGATTGTACCTCATCAGGAGCACGTTCTACCTATGATTCC
Read_8           ------------------------------------------------------------
Read_9           ------------------------------------------------------------
Read_10          ------------------------------------------------------------
Read_11          ------------------------------------------------------------
Read_12          ------------------------------------------------------------
Read_13          ------------------------------------------------------------
Read_14          ------------------------------------------------------------
Read_15          ------------------------------------------------------------
Read_16          ------------------------------------------------------------
Read_17          ------------------------------------------------------------
Read_18          ------------------------------------------------------------
Read_19          ------------------------------------------------------------
Read_20          ------------------------------------------------------------
Read_21          ATACCGTTATTAAAATATGACAACTCAATTAAAC--------------------------
Read_22          ATACTGTTATTAAAATATGACAACTCAATTAAAC--------------------------
Read_23          ------------------------------------------------------------
Read_24          ------------------------------------------------------------
Read_25          ------------------------------------------------------------
Read_26          ------------------------------------------------------------
Read_27          ------------------------------------------------------------
Read_28          ------------------------------------------------------------
Read_29          ------------------------------------------------------------
Read_30          ------------------------------------------------------------
Read_31          ------------------ACAACTCAATTAAA----------------------------
Read_32          ------------------------------------------------------------
Read_33          ------------------------------------------------------------
Read_34          ------------------------------------------------------------
Read_35          ------------------------------------------------------------
Read_36          ------------------------------------------------------------
Read_37          ------------------------------------------------------------
Read_38          ------------------------------------------------------------
Read_39          ------------------------------------------------------------
Read_40          ------------------------------------------------------------


WT               -------------------------------------CAGCGGCGGTGCTTTCAAAGATG
Read_1           ATACCGTTATTAACATATGACAACTCAATTAAAC---CAGCGGCGGTGCTTTCAAAGATG
Read_2           ATACCGTTATTAACATATGACAACTCAATTAAAC---CAGCGGCGGTGCTTTCAAAGATG
Read_3           ATACCGTTATTAACATATGACAACTCAATTAAAC---CAGCGGCGGTGCTTTCAAAGATG
```

```
Read_4          ATACCGTTATTAACATATGACAACTCAATTAAAC----AGCGGCGGTGCTTTCAAAGATG
Read_5          ATACCGTTATTAACATATGACAACTCAATTAAAC---CAGCGGCGGTGCTTTCAAAGATG
Read_6          ATACCGTTATTAACATATGACAACTCAATTAAAC---CAGCGGCGGTGCTTTCAAAGATG
Read_7          GTTTAATTGAGTTGTCATATGTTAATAACGGTAT---CAGCGGCGGTGCTTTCAAAGATG
Read_8          -------------------------------------CAGCGGCGGTGCTTTCAAAGATG
Read_9          -------------------------------------CAGCGGCGGTGCTTTCAAAGATG
Read_10         -------------------------------------CAGCGGCGGTGCTTTCAAAGATG
Read_11         GTTTAATTGAGTTGTCATATGTTAATAACGGTAT---CAGCGGCGGTGCTTTCAAAGATG
Read_12         GTTTAATTGAGTTGTCATATGTTAATAACGGTAT---CAGCGGCGGTGCTTTCAAAGATG
Read_13         GTTTAATTGAGTTGTCATATGTTAATAACGGTAT---CAGCGGCGGTGCTTTCAAAGATG
Read_14         GTTTAATTGAGTTGTCATATGTTAATAACGGTAT---CAGCGGCGGTGCTTTCAAAGATG
Read_15         GTTTAATTGAGTTGTCATATGTTAATAACGGTAT-----------------TCAAAGATG
Read_16         GTTTAATTGAGTTGTCATATGTTAATAACGGTAT-----------------TCAAAGATG
Read_17         GGTTAATTGAGTTTTCATATGTTAATAACTGTAT-----------------TCAAAGATG
Read_18         GTTTAATTGAGTTGTCATATGTTAATAACGGTAT---CAGCGGCGGTGCTTTCAAAGATG
Read_19         GTTTAATTGAGTTGTCATATGTTAATAACGGTAT---CAGCGGCGGTGCTTTCAAAGATG
Read_20         ATACCGTTATTAACATATGACAACTCAATTAAAC----AGCGGCGGTGCTTTCAAAGATG
Read_21         ATAACGTTATTAACATATGACAACTCAATTAAAC---CAGCGGCGGTGCTTTCAAAGATG
Read_22         ATACCGTTATTAACATATGACAACTCAATTAAAC---CAGCGGCGGTGCTTTCAAAGATG
Read_23         ATACCGTTGTTAATTTATGACAACTCAATTAAAC----AGCGGCGGTGCTTTCAAAGATG
Read_24         ATACCGTTATTAACATATGACAACTCAATTAAAC----AGCGGCGGTGCTTTCAAAGATG
Read_25         ATACCGTTATTAACATATGACAACTCAATTAAAC----AGCGGCGGTGCTTTCAAAGATG
Read_26         --TTAATTGAGTTGTCATATGTTAATAACGGTAT-----------------TCAAAGATG
Read_27         ATACCGTTATTAACATATGACAACTCAATTAAAC---CAGCGGCGGTGCTTTCAAAGATG
Read_28         ATACCGTTATTAACATATGACAACTCAATTAAAC---CAGCGGCGGTGCTTTCAAAGATG
Read_29         --ACCGTTATTAACATATGACAACTCAATTAAAC---CAGCGGCGGTGCTTTCAAAGATG
Read_30         ATACCGTTATTAACATATGACAACTCAATTAAAC----AGCGGCGGTGCTTTCAAAGATG
Read_31         ATACCGTTATTAACATATGACAACTCAATTAAAC----AGCGGCGGTGCTTTCAAAGATG
Read_32         ATACCGTTATTAACATATGACAACTCAATTAAAC---CAGCGGCGGTGCTTTCAAAGATG
Read_33         ATTCCGTTATTAACATATGACAACTCAATTAAAC---CAGCGGCGGTGCTTTCAAAGATG
Read_34         ATTCCGTTATTAACATATGACAACTCAATTAAAC---CAGCGGCGGTGCTTTCAAAGATG
Read_35         ATTCCGTTATTAACATATGACAACTCAATTAAAC---CAGCGGCGGTGCTTTCAAAGATG
Read_36         ATACCGTTATTAACATATGACAACTCAATTAAAC----AGCGGCGGTGCTTTCAAAGATG
Read_37         ATACCGTTATTAACATATGACAACTCAATTAAAC----AGCGGCGGTGCTTTCAAAGATG
Read_38         GTTTAATTGAGTTGTCATATGTTAATAACGGTAT---CAGCGGCGGTGCTTTCAAAGATG
Read_39         GTTTAATTGAGTTGTCATATGTTAATAACGGTAT---CAGCGGCGGTGCTTTCAAAGATG
Read_40         GTTTAATTGAGTTGTCATATGTTAATAACGGTAT---CAGCGGCGGTGCTTTCAAAGATG
                                                      *    * **   **********
```

CRISPR+ODN 2-2

```
WT              AAAGATGTTAGACACTCCTGGGCCATACTTGTTGGATGTGATTGTACCTCATCAGGAGCA
Read_1          AAAGATGTTAGACACTCCTGGGCCATACTTGTTGGATGTGATTGTACCTCATCAGGAGCA
Read_2          AAAGATGTTAGACACTCCTGGGCCATACTTGTTGGATGTGATTGAACCTCATCAGGAGCA
Read_3          AAAGATGTTAGACACTCCTGGGCCATACTTGTTGGATGTGATTGTACCTCATCAGGAGCA
Read_4          AAAGATGTTAGACACTCCTGGGCCATACTTGTTGGATGTGATTGTACCTCATCAGGAGCA
Read_5          AAAGATGTTAGACACTCCTGGGCCATACTTGTTGGATGTGATTGTACCTCATCAGGAGCA
Read_6          AAAGATGTTAGACACTCCTGGGCCATACTTGTTGGATGTGATTGTACCTCATCAGGAGCA
Read_7          AAAGATGTTAGACACTCCTGGGCCATACTTGTTGGATGTGATTGTACCTCATCAGGAGCA
Read_8          AAAGATGTTAGACACTCCTGGGCCATACTTGTTGGATGTGATTGTACCTCATCAGGAGCA
Read_9          AAAGATGTTAGACACTCCTGGGCCATACTTGTTGGATGTGATTGTACCTCATCAGGAGCA
Read_10         AAAGATGTTAGACACTCCTGGGCCATACTTGTTGGATGTGATTGTACCTCATCAGGAGCA
Read_11         AAAGATGTTAGACACTCCTGGGCCATACTTGTTGGATGTGATTGTACCTCATCAGGAGCA
Read_12         AAAGATGTTAGACACTCCTGGGCCATACTTGTTGGATGTGATTGTACCTCATCAGGAGCA
Read_13         AAAGATGTTAGACACTCCTGGGCCATACTTGTTGGATGTGATTGTACCTCATCAGGAGCA
Read_14         AAAGATGTTAGACACTCCTGGGCCATACTTGTTGGATGTGATTGTACCTCATCAGGAGCA
Read_15         AAAGATGTTAGACACTCCTGGGCCATACTTGTTGGATGTGATTGTACCTCATCAGGAGCA
Read_16         AAAGATGTTAGACACTCCTGGGCCATACTTGTTGGATGTGATTGTACCTCATCAGGAGCA
Read_17         AAAGATGTTAGACACTCCTGGGCCATACTTGTTGGATGTGATTGTACCTCATCAGGAGCA
Read_18         AAAGATGTTAGACACTCCTGGGCCATACTTGTTGGATGTGATTGTACCTCATCAGGAGCA
Read_19         AAAGATGTTAGACACTCCTGGGCCATACTTGTTGGATGTGATTGTACCTCATCAGGAGCA
Read_20         AAAGATGTTAGACACTCCTGGGCCATACTTGTTGGATGTGATTGTACCTCATCAGGAGCA
Read_21         AAAGATGTTAGACACTCCTGGGCCATACTTGTTGGATGTGATTGTACCTCATCAGGAGCA
Read_22         AAAGATGTTAGACACTCCTGGGCCATACTTGTTGGATGTGATTGTACCTCATCAGGAGCA
Read_23         AAAGATGTTAGACACTCCTGGGCCATACTTGTTGGATGTGATTGTACCTCATCAGGAGCA
Read_24         AAAGATGTTAGACACTCCTGGGCCATACTTGTTGGATGTGATTGTACCTCATCAGGAGCA
Read_25         AAAGATGTTAGACACTCCTGGGCCATACTTGTTGGATGTGATTGTACCTCATCAGGAGCA
Read_26         AAAGATGTTAGACACTCCTGGGCCATACTTGTTGGATGTGATTGTACCTCATCAGGAGCA
Read_27         AAAGATGTTAGACACTCCTGGGCCATACTTGTTGGATGTGATTGTACCTCATCAGGAGCA
Read_28         AAAGATGTTAGACACTCCTGGGCCATACTTGTTGGATGTGATTGTACCTCATCAGGAGCA
Read_29         AAAGATGTTAGACACTCCTGGGCCATACTTGTTGGATGTGATTGTACCTCATCAGGAGCA
```

```
Read_30         AAAGATGTTAGACACTCCTGGGCCATACTTGTTGGATGTGATTGTACCTCATCAGGAGCA
Read_31         AAAGATGTTAGACACTCCTGGGCCATACTTGTTGGATGTGATTGTACCTCATCAGGAGCA
Read_32         AAAGATGTTAGACACTCCTGGGCCATACTTGTTGGATGTGATTGTACCTCATCAGGAGCA
Read_33         AAAGATGTTAGACACTCCTGGGCCATACTTGTTGGATGTGATTGTACCTCATCAGGAGCA
Read_34         AAAGATGTTAGACACTCCTGGGCCATACTTGTTGGATGTGATTGTACCTCATCAGGAGCA
Read_35         AAAGATGTTAGACACTCCTGGGCCATACTTGTTGGATGTGATTGTACCTCATCAGGAGCA
Read_36         AAAGATGTTAGACACTCCTGGGCCATACTTGTTGGATGTGATTGTACCTCATCAGGAGCA
Read_37         AAAGATGTTAGACACTCCTGGGCCATACTTGTTGGATGTGATTGTACCTCATCAGGAGCA
Read_38         AAAGATGTTAGACACTCCTGGGCCATACTTGTTGGATGTGATTGTACCTCATCAGGAGCA
                ******************************************** ***************

WT              ------------------------------------------------------------
Read_1          CGTTCTACCTATGATTCC-TACCGTTATTAACATATGACAACTCAATTAAAC--------
Read_2          CGTTCTACCTATGATTCC------------------------------------------
Read_3          CGTTCTACCTATGATTCC------------------------------------------
Read_4          CGTTCTACCTATGATTCC------------------------------------------
Read_5          CGTTCTACCTATGATTC-------------------------------------------
Read_6          CGTTCTACCTATGATTCC------------------------------------------
Read_7          CGTTCTACCTATGATTCC------------------------------------------
Read_8          CGTTCTACCTATGATTCCATACCGTTATTAACATATGACAACTCAATTAAAC--CAGCTG
Read_9          CGTTCTACCTATGATTCA------------------------------------------
Read_10         CGTTCTACCTATGATTCA------------------------------------------
Read_11         CGTTCTACCTATGATTCC-TACCGTTATTAACATATGACAACTCAATTAAAC--------
Read_12         CGTTCTACCTATGATTCCATACCGTTATTAACATATGACAACTCAATTAAAC--------
Read_13         CGTTCTACCTATGATTCC------------------------------------------
Read_14         CGTTCTACCTATGATTCC------------------------------------------
Read_15         CGTTCTACCTATGATTCCATACCGTTATTAACATATGACAACTCAATTAAAC--------
Read_16         CGTTCTACCTATGATTCA------------------------------------------
Read_17         CGTTCTACCTATGATTC-------------------------------------------
Read_18         CGTTCTACCTATGATTC-------------------------------------------
Read_19         CGTTCTACCTATGATTC-GTTTAATTGAGTTGTCATATGTTAATAACGGTGTT--------
Read_20         ------------------------------------------------------------
Read_21         ------------------------------------------------------------
Read_22         ------------------------------------------------------------
Read_23         CGTTCTACCTATGATTCCATACCGTTATTAACATATGACAACTCAATTAAAC------GT
Read_24         CGTTCTACCTATGATTCCATACCGTTATTAACATATGACAACTCAATTAAAC------GT
Read_25         CGTTCTACCTATGATTCA------------------------------------------
Read_26         CGTTCTACCTATGATTC-------------------------------------------
Read_27         CGTTCTACCTATGATTC-------------------------------------------
Read_28         ------------------------------------------------------------
Read_29         CGTTCTACCTATGATTC-------------------------------------------
Read_30         CGTTCTACCTATGATTCCGTTTAATTGAGTTGTCATATGTTAATAACGGTAT--CAGCGG
Read_31         C-----------------------------------------------------------
Read_32         C-----------------------------------------------------------
Read_33         CGTTCTACCTATGATTCTATACCGTTATTAACATATGACAACTCAATTAAAC--------
Read_34         CGTTCTACCTATGATTCTATACCGTTATTAACATATGACAACTCAATTAAAC--------
Read_35         CGTTCTACCTATGATTCC-TACCGTTATTAACATATGACAACTCAATTAAAC--------
Read_36         CGTTCTACCTATGATTCC-TACCGTTATTAACATATGACAACTCAATTAAAC--------
Read_37         CGTTCTACCTATGATTCCATACCGTTATTAACATATGACAACTCAATTAAAC--CAGCGG
Read_38         CGTTCTACCTATGATTCC------------------------------------------


WT              ------------------------------------------------------------
Read_1          ------------------------------------------------------------
Read_2          ------------------------------------------------------------
Read_3          ------------------------------------------------------------
Read_4          ------------------------------------------------------------
Read_5          ------------------------------------------------------------
Read_6          ------------------------------------------------------------
Read_7          ------------------------------------------------------------
Read_8          CGGTGCTTTCAAAGATGTGATCCCCGAGGGTGATGGGAGACGTTCCTATTGACTTTTAGA
Read_9          ------------------------------------------------------------
Read_10         ------------------------------------------------------------
Read_11         ------------------------------------------------------------
Read_12         ------------------------------------------------------------
Read_13         ------------------------------------------------------------
Read_14         ------------------------------------------------------------
Read_15         ------------------------------------------------------------
Read_16         ------------------------------------------------------------
Read_17         ------------------------------------------------------------
Read_18         --------------------------GTTTAATTGAGTTGTCATATGTTAATAACGGTGT
```

```
Read_19          -----------------------------TAATTGAGTTGTCATATGTTAATAACGGTAT
Read_20          ------------------------------------------------------------
Read_21          ------------------------------------------------------------
Read_22          ------------------------------------------------------------
Read_23          TTAATTGAGTTGTCATATGTTAATAACGGTATATACCGTTATTAACATATGACAACTCAA
Read_24          TTAATTGAGTTGACATATGTTAATAACGGTATATACCGTTATTAACATATGACAACTCAA
Read_25          ------------------------------------------------------------
Read_26          ------------------------------------------------------------
Read_27          ------------------------------------------------------------
Read_28          ------------------------------------------------------------
Read_29          ------------------------------------------------------------
Read_30          CGTTTCTTTCAAAGATGTGATCCCAGAGGGTGATGGGAGACGTTCCTATTTACTTTTAGA
Read_31          GTTTAATTGAGTTGTCATATGTTAATAACGGTATACCGTTATTAACATATGACAACTCAA
Read_32          GTTTAATTGAGTTGTCATATGTTAATAACGGTATACCGTTATTAACATATGACAACTCAA
Read_33          ------------------------------------------------------------
Read_34          ------------------------------------------------------------
Read_35          ------------------------------------------------------------
Read_36          ------------------------------------------------------------
Read_37          CGGTTCTTACAAAGATGTGATCCCAGAGGGTGATGGGAGACGTTCCTATTTACTTTTAGA
Read_38          ------------------------------------------------------------

WT               ------------------------------------------------------------
Read_1           ------------------------------------------------------------
Read_2           ----TTTTAATTGAGTTGTCATATGTTAATAACGGTAT-----ACCGTTATTAACATATG
Read_3           ----GTTTAATTGAGTTGTCATATGTTAATAACGGTAT-----ACCGTTATTAACATATG
Read_4           ----GTTTAATTGAGTTGTCATATGTTAATAACGGTAT-----ACCGTTATTAACATATG
Read_5           ------ATACCGTTATTAACATATGACAACTCAATTAAAC--------------------
Read_6           ------ATACCGTTATTAACATATGACAACTCAATTAAAC--------------------
Read_7           ------ATACCGTTATTAACATATGACAACTCAATTAAAC--------------------
Read_8           AAATACATACTTGTTGGATGTGATTGTACCTCATCAGGAGCACGTTCTACCTATGATTCC
Read_9           -------TACCGTTATTAACATATGACAACTCAATTAAAC--------------------
Read_10          -------TACCGTTATTAACATATGACAACTCAATTAAAC--------------------
Read_11          ------------------------------------------------------------
Read_12          ------ATACCGTTATTAACATATGACAACTCAATTAAAC--------------------
Read_13          ------ATACCGTTATTAACATATGACAACTCAATTAAAC--------------------
Read_14          ------ATACCGTTATTAACATATGACAACTCAATTAAAC--------------------
Read_15          ------ATACCGTTATTAACATATGACAACTCAATTAAAC--------------------
Read_16          -------TACCGTTATTAACATATGACAACTCAATTAAAC--------------------
Read_17          ------ATACCGTTATTAACATATGACAACTCAATTAAAC--------------------
Read_18          ------TTAATTGAGTTGTCATATGTTAATAACGGTAT-----ATACCGTTATTAACATA
Read_19          --------ATACCGTTATTAACATATGACAACTCAATTAAAC------------------
Read_20          ---------TACCGTTATTAACATATGACAACTCAATTAAAC------------------
Read_21          ---------TACCGTTATTAACATATGACAACTCAATTAAAC------------------
Read_22          ---------TACCGTTATTAACATATGACAACTCAATTAAAC------------------
Read_23          --TTAAACATACCGTTATTAACATATGACAACTCAATTAAACTACCGTTATTAACATA--
Read_24          --TTAAACATACCGTTATTAACATATGACAACTCAATTAAACTACCGTTATTAACATA--
Read_25          ---------TACCGTTATTAACATATGACAACTCAATTAAAC------------------
Read_26          --------ATACCGTTATTAACATATGACAACTCAATTAAAC------------------
Read_27          --------ATACCGTTATTAACATATGACAACTCAATTAAAC------------------
Read_28          ---------TACCGTTATTAACATATGACAACTCAATTAAAC------------------
Read_29          ---CGTTTAATTGAGTTGTCATATGTTAATAACGGTAT----------------------
Read_30          AACTACATACTTGTTGGATGTGATTGTACCTCATCAGGAGCACGTTCTACCTATGATTCC
Read_31          TTAAAC--ATACCGTTATTAACATATGACAACTCAATTAAAC------------------
Read_32          TTAAAC--ATACCGTTATTAACATATGACAACTCAATTAAAC------------------
Read_33          --------ATACCGTTATTAACATATGACAACTCAATTAAAC------------------
Read_34          --------ATACCGTTATTAACATATGACAACTCAATTAAAC------------------
Read_35          ------------------------------------------------------------
Read_36          ------------------------------------------------------------
Read_37          AACTACATACTTGTTGGATGTGATTGTACCTCATCAGGAGCACGTTCTACCTATGATTCC
Read_38          --------ATACCGTTATTAACATATGACAACTCAATTAAAC------------------

WT               -----------------------------------CCAGCGGCGGTGCTTTCAAAGATGT
Read_1           -----------------------------------CCAGCGGCGGTGCTTTCAAAGATGT
Read_2           ACAACTCAATTAAACC--------------------CAGCGGCGGTGCTTTCAAAGATGT
Read_3           ACAACTCAATTAAACC--------------------CAGCGGCGGTGCTTTCAAAGATGT
Read_4           ACAACTCAATTAAACC--------------------CAGCGGCGGTGCTTTCAAAGATGT
Read_5           ------------------------------------CAGCGGCGGTGCTTTCAAAGATGT
Read_6           ------------------------------------CAGCGGCGGTGCTTTCAAAGATGT
Read_7           ------------------------------------CAGCGGCGGTGCTTTCAAAGATGT
```

```
Read_8          ATACCGTTATTAACATATGACAACTCAATTAAAC--CAGCGGCGGTGCTTTCAAAGATGT
Read_9          ------------------------------------CAGCGGCGGTGCTTTCAAAGATGT
Read_10         ------------------------------------CAGCGGCGGTGCTTTCAAAGATGT
Read_11         -----------------------------------CCAGCGGCGGTGCTTTCAAAGATGT
Read_12         ------------------------------------CAGCGGCGGTGCTTTCAAAGATGT
Read_13         ------------------------------------CAGCGGCGGTGCTTTCAAAGATGT
Read_14         ------------------------------------CAGCGGCGGTGCTTTCAAAGATGT
Read_15         ------------------------------------CAGCGGCGGTGCTTTCAAAGATGT
Read_16         ------------------------------------CAGCGGCGGTGCTTTCAAAGATGT
Read_17         ------------------------------------CAGCGGCGGTGCTTTCAAAGATGT
Read_18         TGACAACTCAATTAAAC-------------------CAGCGGCGGTGCTTTCAAAGATGT
Read_19         ------------------------------------CAGCGGCGGTGCTTTCAAAGATGT
Read_20         --------------------------------------GCGGCGGTGCTTTCAAAGATGT
Read_21         --------------------------------------GCGGCGGTGCTTTCAAAGATGT
Read_22         --------------------------------------GCGGCGGTGCTTTCAAAGATGT
Read_23         TGACAACTCAATTAAAC-------------------CAGCGGCGGTGCTTTCAAAGATGT
Read_24         TGACAACTCAATTAAAC-------------------CAGCGGCGGTGCTTTCAAAGATGT
Read_25         ------------------------------------CAGCGGCGGTGCTTTCAAAGATGT
Read_26         ------------------------------------CAGCGGCGGTGCTTTCAAAGATGT
Read_27         ------------------------------------CAGCGGCGGTGCTTTCAAAGATGT
Read_28         --------------------------------------GCGGCGGTGCTTTCAAAGATGT
Read_29         ------------------------------------CAGCGGCGGTGCTTTCAAAGATGT
Read_30         GTTTAATTGAGTTGTCATATGTTAATAACGGTAT--CAGCGGCGGTGCTTTCAAAGATGT
Read_31         ------------------------------------CAGCGGCGGTGCTTTCAAAGATGT
Read_32         ------------------------------------CAGCGGCGGTGCTTTCAAAGATGT
Read_33         ------------------------------------CAGCGGCGGTGCTTTCAAAGATGT
Read_34         ------------------------------------CAGCGGCGGTGCTTTCAAAGATGT
Read_35         ------------------------------------CAGCGGCGGTGCTTTCAAAGATGT
Read_36         ------------------------------------CAGCGGCGGTGCTTTCAAAGATGT
Read_37         ATACCGTTATTAACATATGACAACTCAATTAAAC--CAGCGGCGGTGCTTTCAAAGATGT
Read_38         ------------------------------------CAGCGGCGGTGCTTTCAAAGATGT
                                                      **********************
```

CRISPR+ODN 2-3

```
WT              GCACGTTCTACCTATGATTC----------------------------------------
Read_1          GCACGTTCTACCTATTATTCCTTTTAATTGAGTTGTCATATGTTAATAACGGTAT-TTAA
Read_2          GCACGTTCTACCTATGATTCCGTTTAATTGAGTTGTCATATGTTAATAACGGTAT-TTAA
Read_3          GCACGTTCTACCTATGATTCCA--------------------------------------
Read_4          GCACGTTCTACCTATGATTCCATACCGTTATTAACATATGACAACTCAATTAAACCAGCG
Read_5          GCACGTTCTACCTATGATTC----------------------------------------
Read_6          GCACGTTCTACCTATGATTCC---------------------------------------
Read_7          GCACGTTCTACCTA-------ATACCGTTATTAACATATGACAACTCAATTAAAC-----
Read_8          GCACGTTCTACCTATGATTC-GTTTAATTGAGTTGTCATATGTTAATAACGGTAT-----
Read_9          GCACGTTCTACCTATGATTC-GTTTAATTGAGTTGTCATATGTTAATAACGGTAT-----
Read_10         GCACGTTCTACCTATGATTC-GTTTAATTGAGTTGTCATATGTTAATAACGGTAT-----
Read_11         GCACGTTCTACCTATGATTCCATACCGTTATTAACATATGACAACTCAATTAA-------
Read_12         GCACGTTCTACCTATGATTA----------------------------------------
Read_13         GCACGTTCTACCTATGATTA----------------------------------------
Read_14         GCACGTTCTACCTATGATTCCCTACCGTTATTAACATATGACAACTCAATTAAAC-----
Read_15         GCACGTTCTACCTATGATTCCATACCGTTATTAACATATGACAACTCAATTAAAC-----
Read_16         GCACGTTCTACCTATGATTCCGTTTAATTGAGTTGTCATATGTTAATAACGGTAT------
Read_17         GCACGTTCTACCTATGATTC----------------------------------------
Read_18         GCACGTTCTACCTATGATTC----------------------------------------
Read_19         GCACGTTCTACCTATGATTC----------------------------------------
Read_20         GCACGTTCTACCTATGATTCA---------------------------------------
Read_21         GCACGTTCTACCTATGATTCA---------------------------------------
Read_22         GCACGTTCTACCTATGATTA----------------------------------------
Read_23         GCACGTTCTACCTATGATTA----------------------------------------
Read_24         GCACGTTCTACCTATGATTCCT--------------------------------------
Read_25         GCACGTTCTACCTATGATTCA---------------------------------------
Read_26         GCACGTTCTACCTATGAATA----------------------------------------
Read_27         GCACGTTCTACCTATGATTA----------------------------------------
Read_28         GCACGTTCTACCTATGATTA----------------------------------------
Read_29         GCACGTTCTACCTATGATTCCGTTTAATTGAGTTGTCATATGTTAATAACGGTA-TTTAA
Read_30         GCACGTTCTACCTATGATTCA---------------------------------------
Read_31         GCACGTTCTACCTATGATTCA---------------------------------------
Read_32         GCACGTTCTACCTATGATTCCGTTTAATTGAGTTGTCATATGTTAATAACGGTAGTTTAA
Read_33         GCACGTTCTACCTATGATTCCCTTTAATTGAGCTGTCATATGTTAATAACGGTAGTTTAA
Read_34         GCACGTTCTACCTATGATTCC---------------------------------------
Read_35         GCACGTTCTACCTATGATTCCT--------------------------------------
```

```
Read_36          GCACGTTCTACCTATGAATA----------------------------------------
Read_37          GCACGTTCTACCTATGAATA----------------------------------------
Read_38          GCACGTTCTACCTATGATTCCGTTTAATTGAGTTGTCATATGTTAATAACGGTAGTTTAA
Read_39          GCACGTTCTACCTATGATTCC---------------------------------------
Read_40          GCACGTTCTACCTATGATTCCATACCGTTATTAACATATGACAACTCAATTAAACCAGCG
Read_41          GCACGTTCTACCTATGATTCGTTTAATTGAGTTGTCATATGCTAATAACGGTAT------
Read_42          GCACGTTCTACCTATGATTC----------------------------------------
Read_43          GCACGTTCTACCTATGATTC----------------------------------------
Read_44          GCACGTTCTACCTA-------ATACCGTTATTAACATATGACAACTCAATTAAA------
Read_45          GCACGTTCTACCTATGATTCCATACCGTTATTAACATATGACAACTCAATTAAACCAGCG
                 **************  *

WT               ------------------------------------------------------------
Read_1           TTGATTTGTCATATGTTAATAACGGTAT--------------------------------
Read_2           TTGAGTTGTCATATGTTAATAACGGTAT--------------------------------
Read_3           ------------------------------------------------------------
Read_4           GCGGTGCTTTCAAAGATGTGATCACAGAGGGTGATGGGAGACGTTCCTATTGACTTTTAG
Read_5           ------------------------------------------------------------
Read_6           ------------------------------------------------------------
Read_7           ------------------------------------------------------------
Read_8           ------------------------------------------------------------
Read_9           TTAATAACGGTA------------------------------------------------
Read_10          TTAATAACGGTA------------------------------------------------
Read_11          ------------------------------------------------------------
Read_12          ------------------------------------------------------------
Read_13          ------------------------------------------------------------
Read_14          ------------------TGATC-------------------------------------
Read_15          ------------------------------------------------------------
Read_16          TTGAGTTGTCATATGGTTTAATTAATAACGGTAT--------------------------
Read_17          TTGAGTTGTCATATGGTTTAATTAATAACGGTAT--------------------------
Read_18          TTGAGTTGTCATATGGTTTAATTAATAACGGTAT--------------------------
Read_19          TTGAGTTGTCATATGGTTTAATTAATAACGGTAT--------------------------
Read_20          ------------------------------------------------------------
Read_21          ------------------------------------------------------------
Read_22          ------------------------------------------------------------
Read_23          ------------------------------------------------------------
Read_24          ------------------------------------------------------------
Read_25          ------------------------------------------------------------
Read_26          ------------------------------------------------------------
Read_27          ------------------------------------------------------------
Read_28          ------------------------------------------------------------
Read_29          TTGAGTTGTCATATG------TTAATAACGGTAT--------------------------
Read_30          ------------------------------------------------------------
Read_31          ------------------------------------------------------------
Read_32          TTGAGTTGTCATATG------TTAATAACGGTAT-------------------------
Read_33          TTGAGTTGTCATATG------TTAATAACGGTAT--------------------------
Read_34          ------------------------------------------------------------
Read_35          ------------------------------------------------------------
Read_36          ------------------------------------------------------------
Read_37          ------------------------------------------------------------
Read_38          TTGAGTTGTCACATG------TTAATAACGGTAT--------------------------
Read_39          ------------------------------------------------------------
Read_40          GCGGTGCTCTCAAAGATGTGATCACAGAGGGTGATGGGAGACGTTCCTATTGACTTTTAG
Read_41          ------------------------------------------------------------
Read_42          GTTTAATTGAGTTGTCATATGTTAATAACGGTAT--------------------------
Read_43          GTTTAATTGAGTTGTCATATGTTAATAACGGTAT--------------------------
Read_44          ------------------------------------------------------------
Read_45          GCGTTGCTTTCAAAGATGTGATCACAGAGGGTGATGGGAGACGTTCCTATTGACTTTTAG


WT               ------------------------------------------------------------
Read_1           ------------------------------------------------------------
Read_2           ------------------------------------------------------------
Read_3           ------------------------------------------------------------
Read_4           AAACTACATACTTGTTGGATGTGATTGTACCTCATCAGGAGCAGGTTCTACCTATGATTC
Read_5           ------------------------------------------------------------
Read_6           ------------------------------------------------------------
Read_7           ------------------------------------------------------------
Read_8           ------------------------------------------------------------
Read_9           ------------------------------------------------------------
Read_10          ------------------------------------------------------------
```

```
Read_11         ------------------------------------------------------------
Read_12         ------------------------------------------------------------
Read_13         ------------------------------------------------------------
Read_14         ------------------------------------------------------------
Read_15         -----------------------------------------------------------T
Read_16         ------------------------------------------------------------
Read_17         ------------------------------------------------------------
Read_18         ------------------------------------------------------------
Read_19         ------------------------------------------------------------
Read_20         ------------------------------------------------------------
Read_21         ------------------------------------------------------------
Read_22         ------------------------------------------------------------
Read_23         ------------------------------------------------------------
Read_24         ------------------------------------------------------------
Read_25         ------------------------------------------------------------
Read_26         ------------------------------------------------------------
Read_27         ------------------------------------------------------------
Read_28         ------------------------------------------------------------
Read_29         ------------------------------------------------------------
Read_30         ------------------------------------------------------------
Read_31         ------------------------------------------------------------
Read_32         ------------------------------------------------------------
Read_33         ------------------------------------------------------------
Read_34         ------------------------------------------------------------
Read_35         ------------------------------------------------------------
Read_36         ------------------------------------------------------------
Read_37         ------------------------------------------------------------
Read_38         ------------------------------------------------------------
Read_39         ------------------------------------------------------------
Read_40         AAAATACATACTTGTTGGATGTGATTGTACCTCATCAGGAGCACGTTCTACCTATGATTC
Read_41         ------------------------------------------------------------
Read_42         ------------------------------------------------------------
Read_43         ------------------------------------------------------------
Read_44         ------------------------------------------------------------
Read_45         AAACTACATACTTGTTGGATGTGATTGTACCTCATCAGGAGCACGTTCTACCTATGATTC


WT              ----------------------------------CC-AGCGGCGGTGCTTTCAAAGATGT
Read_1          -ATACAGTTATTAACATATGACAACTCAATTAAACC-AGCGGCGGTGCTTTCAAAGATGT
Read_2          -ATACCGTTATTAACATATGACAACTCAATTAAACC-AGCGGCGGTGCTTTCAAAGATGT
Read_3          --TACCGTTATTAACATATGACAACTCAATTAAACC-AGCGGCGGTGCTTTCAAAGATGT
Read_4          CATACCGTTATTAACATATGACAACTCAATTAAACC-AGCGGCGGTGCTTTCAAAGATGT
Read_5          -ATACCGTTATTAACATATGACAACTCAATTAAACC-AGCGGCGGTGCTTTCAAAGATGT
Read_6          --TACCGTTATTAACATATGACAACTCAATTAAACC-AGCGGCGGTGCTTTCAAAGATGT
Read_7          -ATACCGTTATTAACATATGACAACTCAATTAAACCCAGCGGCGGTGCTTTCAAAGATGT
Read_8          -ATACCGTTATTAACATATTACAACGCAATTAAACC-AGCGGCGGTGCTTTCAAAGATGT
Read_9          -ATACCGTTATTAACATATGACAACTCAATTAAACC-AGCGGCGGTGCTTTCAAAGATGT
Read_10         -ATACCGTTATTAACATATGACAACTCAATTAAACC-AGCGGCGGTGCTTTCAAAGATGT
Read_11         ACAACCGTTATTAACATATGACAACTCAATTAAACA--GCGGCGGTGCTTTCAAAGATGT
Read_12         --TACCGTTATTAACATATGACAACTCAATTAAAC-----GGCGGTGCTTTCAAAGATGT
Read_13         --TACCGTTATTAACATATGACAACTCAATTAAAC-----GGCGGTGCTTTCAAAGATGT
Read_14         ----CCGTTATTAACATATGACAACTCAATTAAACC-AGCGGCGGTGCTTTCAAAGATGT
Read_15         -ATACCGTTATTAACATATGACAACTCAATTAAACC-AGCGGCGGTGCTTTCAAAGATGT
Read_16         -ATACCGTTATTAACATATGACAACTCAATTAAACC-AGCGGCGGTGCTTTCAAAGATGT
Read_17         -ATACCGTTATTAACATATGACAACTCAATTAAACC-AGCGGCGGTGCTTTCAAAGATGT
Read_18         -ATACCGTTATTAACATATGACAACTCAATTAAACC-AGCGGCGGTGCTTTCAAAGATGT
Read_19         -ATACCGTTATTAACATATGACAACTCAATTAAACC-AGCGGCGGTGCTTTCAAAGATGT
Read_20         --TACCGTTATTAACATATGACAACTCAATTAAACC-AGCGGCGGTGCTTTCAAAGATGT
Read_21         --TACCGTTATTAACATATGACAACTCAATTAAACC-AGCGGCGGTGCTTTCAAAGATGT
Read_22         --TACCGTTATTAACATATGACAACTCAATTAAAC-----GGCGGTGCTTTCAAAGATGT
Read_23         --TACCGTTATTAACATATGACAACTCAATTAAAC-----GGCGGTGCTTTCAAAGATGT
Read_24         ---ACCGTTATTAACATATGACAACTCAATTAAACC-AGCGGCGGTGCTTTCAAAGATGT
Read_25         --TACCGTTATTAACATATGACAACTCAATTAAACC-AGCGGCGGTGCTTTCAAAGATGT
Read_26         ----CCGTTATTAACATATGACAACTCAATTAAACC-AGCGGCGGTGCTTTCAAAGATGT
Read_27         --TACCGTTATTAACATATGACAACTCAATTAAAC-----GGCGGTGCTTTCAAAGATGT
Read_28         --TACCGTTATTAACATATGACAACTCAATTAAAC-----GGCGGTGCTTTCAAAGATGT
Read_29         -ATACCGTTATTAACATATGACAACTCAATTAAACC-AGCGGCGGTGCTTTCAAAGATGT
Read_30         --TACCGTTATTAACATATGACAACTCAATTAAACC-AGCGGCGGTGCTTTCAAAGATGT
Read_31         --TACCGTTATTAACATATGACAACTCAATTAAACC-AGCGGCGGTGCTTTCAAAGATGT
Read_32         -ATACCGTTATTAACATATGACAACTCAATTAAACC-AGCGGCGGTGCTTTCAAAGATGT
Read_33         -ATACCGTTATTAACATATGACAACTCAATTAAACC-AGCGGCGGTGCTTTCAAAGATGT
```

```
Read_34          --TACCGTTATTAACATATGACAACTCAATTAAACC-AGCGGCGGTGCTTTCAAAGATGT
Read_35          ---ACCGTTATTAACATATGACAACTCAATTAAACC-AGCGGCGGTGCTTTCAAAGATGT
Read_36          ----CCGTTATTAACATATGACAACTCAATTAAACC-AGCGGCGGTGCTTTCAAAGATGT
Read_37          ----CCGTTATTAACATATGACAACTCAATTAAACC-AGCGGCGGTGCTTTCAAAGATGT
Read_38          -ATACCGTTATTAACATATGACAACTCAATTAAACC-AGCGGCGGTGCTTTCAAAGATGT
Read_39          -ATACCGTTATTAACATATGACAACTCAATTAAACC-AGCGGCGGTGCTTTCAAAGATGT
Read_40          CATACCGTTATTAACATATGACAACTCAATTAAACC-AGCGGCGGTGCTCTCAAAGATGT
Read_41          -ATACCGTTATTAACATATGACAACTCAATTAAACC-AGCGGCGGTGCTTTCAAAGATGT
Read_42          -ATACCGTTATTAACATATGACAACTCAATTAAACC-AGCGGCGGTGCTTTCAAAGATGT
Read_43          -ATACCGTTATTAACATATGACAACTCAATTAAACC-AGCGGCGGTGCTTTCAAAGATGT
Read_44          CATACCGTTATTAACATATGACAACTCAATTAAACCCAGCGGCGGTGCTTTCAAAGATGT
Read_45          CATACCGTTATTAACATATGACAACTCAATTAAACC-AGCGGCGGTGCTTTCAAAGATGT
                                                   *     ********* **********
```

CRISPR+ODN 3-1

```
WT               AAAGATGTTAGACACTCCTGGGCCATACTTGTTGGATGTGATTGTACCTCATCAGGA---
Read_1           AAAGATGTTAGACACTCCTGGGCCATACTTGTTGGATGTGATTGTACCTCATCAGGAGCA
Read_2           AAAGATGTTAGACACTCCTGGGCCATACTTGTTGGATGTGATTGTACCTCATCAGGAGCA
Read_3           AAAGATGTTAGACACTCCTGGGCCATACTTGTTGGATGTGATTGTACCTCATCAGGAGCA
Read_4           AAAGATGTTAGACACTCCTGGGCCATACTTGTTGGATGTGATTGTACCTCATCAGGAGCA
Read_5           AAAGATGTTAGACACTCCTGGGCCATACTTGTTGGATGTGATTGTACCTCATCAGGAGCA
Read_6           AAAGATGTTAGACACTCCTGGGCCATACTTGTTGGATGTGATTGTACCTCATCAGGAGCA
Read_7           AAAGATGTTAGACACTCCTGGGCCATACTTGTTGGATGTGATTGTACCTCATCAGGAGCA
Read_8           AAAGATGTTAGACACTCCTGGGCCATACTTGTTGGATGTGATTGTACCTCATCAGGA---
Read_9           AAAGATGTTAGACACTCCTGGGCCATACTTGTTGGATGTGATTGTACCTCATCAGGA---
Read_10          AAAGATGTTAGACACTCCTGGGCCATACTTGTTGGATGTGATTGTACCTCATCAGGAGCA
Read_11          AAAGATGTTAGACACTCCTGGGCCATACTTGTTGGATGTGATTGTACCTCATCAGGAGCA
Read_12          AAAGATGTTAGACACTCCTGGGCCATACTTGTTGGATGTGATTGTACCTCATCAGGA---
Read_13          AAAGATGTTAGACACTCCTGGGCCATACTTGTTGGATGTGATTGTACCTCATCAGGA---
                 *********************************************************

WT               ------------------------------------------------------------
Read_1           CGTTCTACCTATGA-------------------------------------------
Read_2           CGTTCTACCTATGA-------------------------------------------
Read_3           CGTTCTACCTATGA-------------------------------------------
Read_4           CGTTCTACCTATGATTC-GTTTAATTGAGTTGTCATATGTTAATAACGGTATGTTTAATT
Read_5           CGTTCTACCTATGATTC-GTTTAATTGAGTTGTCATATGTTAATAACGGTATGTTTAATT
Read_6           CGTTCTACCTATGATTC-GTTTAATTGAGTTGTCATATGTTAATAACGGTATGTTTAATT
Read_7           CGTTCTACCTATGATTC-GTTTCATTGAGTTGTCATATGTTAATAACGGTATGTTTAATT
Read_8           ------------------------------------------------------------
Read_9           ------------------------------------------------------------
Read_10          CGTTCTACCTATGATTCCGTTTAATTGAGTTGTCATATGTTAATAACGGTATGTTTAATT
Read_11          CGTTCTACCTATGATTCCGTTTAATTGAGTTGTCATATGTTAATAACGGTATGTTTAATT
Read_12          ------------------------------------------------------------
Read_13          ------------------------------------------------------------

WT               -----------------------------GCACGTTCTACCTATGAT---TC-------C
Read_1           -------------------------GTTTAATTGAGTTGTCATATGTTAATAACGGTATG
Read_2           -------------------------GTTTAATTGAGTTGTCATATGTTAATAACGGTATG
Read_3           -------------------------GTTTAATTGAGTTGTCATATGTTAATAACGGTATG
Read_4           GAGTTGTCATATGTTAATAACGGT-TATACCGTTATTAACATATGACAACTCAATTAAAC
Read_5           GAGTTGTCATATGTTAATAACGG-ATATACCGTTATTAACATATGACAACTCAATTAAAC
Read_6           GAGTTGTCATATGTTAATAACGG-ATATACCGTTATTAACATATGACAACTCAATTAAAC
Read_7           GAGTTGTCATATGTTAATAACG-TAT-ATACCGTTATTAACATATGACAACTCAATTAAAC
Read_8           --------------------------ATACCGTTATTAACATATGACAACTCAATTAAAC
Read_9           --------------------------ATACCGTTATTAACATATGACAACTCAATTAAAC
Read_10          GAGTTGTCATATGTTAATAACGGTATATACCGTTATTAACATATGACAACTCAATTAAAC
Read_11          GAGTTGTCATATGTTAATAACGGTATATACCGTTATTAACATATGACAACTCAATTAAAC
Read_12          --------------------------ATACCGTTATTAACATATGACAACTCAATTAAAC
Read_13          --------------------------ATACCGTTATTAACATATGACAACTCAATTAAAC
                                                   *   * ****     *

WT               CAGCGGCGGTGCTTTCAAAGATGTGATCACAGAGGGTGATGGGAGACGTTCCTATTGACT
Read_1           CAGCGGCGGTGCTTTCAAAGATGTGATCACAGAGGGTGATGGGAGACGTTCCTATTGACT
Read_2           CAGCGGCGGTGCTTTCAAAGATGTGATCACAGAGGGTGATGGGAGACGTTCCTATTGACT
Read_3           CAGCGGCGGTGCTTTCAAAGATGTGATCACAGAGGGTGATGGGAGACGTTCCTATTGACT
Read_4           CAGCGGCGGTGCTTTCAAAGATGTGATCACAGAGGGTGATGGGAGACGTTCCTATTGACT
Read_5           CAGCGGCGGTGCTTTCAAAGATGTGATCACAGAGGGTGATGGGAGACGTTCCTATTGACT
Read_6           CAGCGGCGGTGCTTTCAAAGATGTGATCACAGAGGGTGATGGGAGACGTTCCTATTGACT
```

```
Read_7          CAGCGGCGGTGCTTTCAAAGATGTGATCACAGAGGGTGATGGGAGACGTTCCTATTGACT
Read_8          CAGCGGCGGTGCTTTCAAAGATGTGATCACAGAGGGTGATGGGAGACGTTCCTATTGACT
Read_9          CAGCGGCGGTGCTTTCAAAGATGTGATCACAGAGGGTGATGGGAGACGTTCCTATTGACT
Read_10         CAGCGGCGGTGCTTTCAAAGATGTGATCACAGAGGGTGATGGGAGACGTTCCTATTGACT
Read_11         CAGCGGCGGTGCTTTCAAAGATGTGATCACAGAGGGTGATGGGAGACGTTCCTATTGACT
Read_12         CAGCGGCGGTGCTTTCAAAGATGTGATCACAGAGGGTGATGGGAGACGTTCCTATTGACT
Read_13         CAGCGGCGGTGCTTTCAAAGATGTGATCACAGAGGGTGATGGGAGACGTTCCTATTGACT
                ************************************************************
```

CRISPR+ODN 3-2

```
WT              CGTTCTACCTATGATTCC------------------------------------------
Read_1          CGTTCTACCTATGATTCGTTTAATTGAGTTGTCATATGTTAATAACGGTAGTTTAATTGA
Read_2          CGTTCTACCTATGATTCGTTTAATTGAGTTGTCATATGTTAATAACGGTAGTTTAATTGA
Read_3          CGTTCTACCTATGATTCGTTTAATTGAGTTGTCATATGTTAATAACGGTAGTTTAATTGA
Read_4          CGTTCTACCTATGATTCGTTTAATTGAGTTGTCATATGTTAATAACGGTAGTTTAATTGA
Read_5          CGTTCTACCTATGATTCC--------------------------------GTTTAATTGA
Read_6          CGTTCTACCTATGATTCATA------------------------------GTTTAATTGA
Read_7          CGTTCTACCTATGATTCATA------------------------------GTTTAATTGA
Read_8          CGTTCTACCTATGATTCATA------------------------------GTTTAATTGA
Read_9          CGTTCTACCTATGATTCC--------------------------------GTTTAATTGA
Read_10         CGTTCTACCTATGATTCGTTTAATTGAGTTGTCATATGTTAATAACGG--GTTTAATTGA
Read_11         CGTTCTACCTATGATTCATA------------------------------GTTTAATTGA
Read_12         CGTTCTACCTATGATTCGTTTAATTGAGTTGTCATATGTTAATAACGGTAGTTTAATTGA
Read_13         CGTTCTACCTATGATTCGTTTAATTGAGTTGTCATATGTTAATAACGGTAGTTTAATTGA
                *****************

WT              ----------------------------------------------------------CA
Read_1          GTTGTCATATGTTAATAACGGTTTATACCGTTATTAACATATGACAACTCAATTAAACCA
Read_2          GTTGTCATATGTTAATAACGGTATATACCGTTATTAACATATGACAACTCAATTAAACCA
Read_3          GTTGTCATATGTTAATAACGGTATATACCGTTATTAACATATGACAACTCAATTAAACCA
Read_4          GTTGTCATATGTTAATAACGGTATATACCGTTATTAACATATGACAACTCAATTAAACCA
Read_5          GTTGTCATATGTTAATAACGGTAT----------------------------------CA
Read_6          GTTGTCATATGTTAATAACGGTAT------------------------------------
Read_7          GTTGTCATATGTTAATAACGGTAT------------------------------------
Read_8          GTTGTCATATGTTAATAACGGTAT------------------------------------
Read_9          GTTGTCATATGTTAATAACGGTATC---------------------------------CA
Read_10         GTTGTCATATGTTAATAACGGT--ATACCGTTATTAACATATGACAACTCAATTAAACCA
Read_11         GTTGTCATATGTTAATAACGGTAT------------------------------------
Read_12         GTTGTCATATGTTAATAACGGTATATACCGTTATTAACATATGACAACTCAATTAAACCA
Read_13         GTTGTCATATGTTAATAACGGTATATACCGTTATTAACATATGACAACTCAATTAAACCA

WT              GCGGCGGTGCTTTCAAAGATGTGATCACAGAGGGTGATGGGAGACGTTCCTATTGACTTT
Read_1          GCGGCGGTGCTTTCAAAGATGTGATCACAGAGGGTGATGGGAGACGTTCCTATTGACTTT
Read_2          GCGGCGGTGCTTTCAAAGATGTGATCACAGAGGGTGATGGGAGACGTTCCTATTGACTTT
Read_3          GCGGCGGTGCTTTCAAAGATGTGATCACAGAGGGTGATGGGAGACGTTCCTATTGACTTT
Read_4          GCGGCGGTGCTTTCAAAGATGTGATCACAGAGGGTGATGGGAGACGTTCCTATTGACTTT
Read_5          GCGGCGGTGCTTTCAAAGATGTGATCACAGAGGGTGATGGGAGACGTTCCTATTGACTTT
Read_6          ---------------------TGATCACAGAGGGTGATGGGAGACGTTCCTATTGACTTT
Read_7          ---------------------TGATCACAGAGGGTGATGGGAGACGTTCCTATTGACTTT
Read_8          ---------------------TGATCACAGAGGGTGATGGGAGACGTTCCTATTGACTTT
Read_9          GCGGCGGTGCTTTCAAAGATGTGATCACAGAGGGTGATGGGAGACGTTCCTATTGACTTT
Read_10         GCGGCGGTGCTTTCAAAGATGTGATCACAGAGGGTGATGGGAGACGTTCCTATTGACTTT
Read_11         ---------------------TGATCACAGAGGGTGATGGGAGACGTTCCTATTGACTTT
Read_12         GCGGCGGTGCTTTCAAAGATGTGATCACAGAGGGTGATGGGAGACGTTCCTATTGACTTT
Read_13         GCGGCGGTGCTTTCAAAGATGTGATCACAGAGGGTGATGGGAGACGTTCCTATTGACTTT
                                     ***************************************
```

CRISPR+ODN 3-3

```
WT              GCACGTTCTACCTATGATTCC---------------------------------------
Read_1          GCACGTTCTACCTATGATTCC---------------------------------------
Read_2          GCACGTTCTACCTATGATTCCATACCGTTATTAACATATGACAACTCAATTAAACCAGCG
Read_3          GCACGTTCTACCTATGATTC-ATACCGTTATTAAC-TATGACAACTCAATTAAAC
Read_4          GCACGTTCTACCTATGATTC-ATACCGTTATTAAC-TATGACAACTCAATTAAAC-----
Read_5          GCACGTTCTACCTATACCGTTATTAACATAT--------GACAATTCAATTAAAC-----
Read_6          GCACGTTCTACCTATGATTCC-----------------------------
Read_7          GCACGTTCTACCTATGATTCCATACCGTTATTAACATATGACAACTCAATTAAACCAGCG
Read_8          GCACGTTCTACCTATGATACC---------------------------------------
Read_9          GCACGTTCTACCTATGATACC---------------------------------------
```

```
Read_10         GCACGTTCTACCTATGATACC---------------------------------------
                ***************

WT              ------------------------------------------------------------
Read_1          ------------------------------------------------------------
Read_2          GCGGTGCTTGCAAAGATCTGATCACAGAGGGTGATGGGAGACGTTCCTATCGACTTTTAG
Read_3          ------------------------------------------------------------
Read_4          ------------------------------------------------------------
Read_5          -----------------GTTTAATTGAGTTGTCATATGTTAATAACGGTAT---------
Read_6          -----------------GTTTAATTGAGTTGTCATATGTTAATAACGGTAT--ATACCGT
Read_7          GCGGTGCTTCCAAAGATGTGATCACAGAGGGTGATGGGAGACGTTCCTATTGACTTTTAG
Read_8          ------------------------------------------------------------
Read_9          ------------------------------------------------------------
Read_10         ------------------------------------------------------------


WT              ------------------------------------------------------------
Read_1          ------------------------------------------------------------
Read_2          AAAATACATATTTGTTGGATGTGATTGTACCTCATCAAGAGCAGGTTCTACCTATGATTC
Read_3          ------------------------------------------
Read_4          ------------------------------------------
Read_5          ---------------------------ATACCGTTATTAACATATGACAACT--------
Read_6          TATTAACATATGACAACTCAATTAAACATACCGTTATTAACATATGACAACTCAATTAAA
Read_7          AAACTACATACTTGTTGGATGTGATTGTACCTCATCAGGAGCACGTTCTACCTATGATTC
Read_8          ------------------------------------------------------------
Read_9          ------------------------------------------------------------
Read_10         ------------------------------------------------------------


WT              ------------------------------------CAGCGGCGGTGCTTTCAAAGATGT
Read_1          -ATACCGTTATTAACATATGACAACTCAATTAAAC-CAGCGGCGGTGCTTTCAAAGATGT
Read_2          CATACCGTTATTAACATATGACAACTCAATTAAAC-CAGCGGCGGTGCTTTCAAAGATGT
Read_3          -----CGTTATTAACATATGACAACTCAATTAAAC-CAGCGGCGGTGCTTTCAAAGATGT
Read_4          -----CGTTATTAACATATGACAACTCAATTAAAC-CAGCGGCGGTGCTTTCAAAGATGT
Read_5          CATACCGTTATTAACATATGACAACTCAATTAAACCCAGCGGCGGTGCTTTCAAAGATGT
Read_6          CATACCGTTATTAACATATGACAACTCAATTAAAC-CAGCGGCGGTGCTTTCAAAGATGT
Read_7          CATACCGTTATTAACATATGACAACTCAATTAAAC-CAGCGGCGGTGCTTTCAAAGATGT
Read_8          ------GTTATTAACATATGACAACTCAATTAAAC-CAGCGGCGGTGCTTTCAAAGATGT
Read_9          ------GTTATTAACATATGACAACTCAATTAAAC-CAGCGGCGGTGCTTTCAAAGATGT
Read_10         ------GTTATTAACATATGACAACTCAATTAAAC-CAGCGGCGGTGCTTTCAAAGATGT
                                                    ************************
```

TALENs+ODN 1-1

```
WT              TCAAAAGATGTTAGACACTCCTGGGCCATACTTGTTGGATGTGATTGTACCTCATCAGGA
Read_1          TCAAAAGATGTTAGACACTCCTGGGCCATACTTGTTGGATGTGATTGTACCTCATCAGGA
Read_2          TCAAAAGATGTTAGACACTCCTGGGCCATACTTGTTGGATGTGATTGTACCTCATCAGGA
Read_3          TCAAAAGATGTTAGACACTCCTGGGCCATACTTGTTGGATGTGATTGTACCTCATCAGGA
Read_4          TCAAAAGATGTTAGACACTCCTGGGCCATACTGGATGGATGTGATTGTACCTCATCAGGA
Read_5          TCAAAAGATGTTAGACACTCCTGGGCCATACTTGTTGGATGTGATTGTACCTCATCAGGA
Read_6          TCAAAAGATGTTAGACACTCCTGGGCCATACTTGTTGGATGTGATTGTACCTCATCAGGA
Read_7          TCAAAAGATGTTAGACACTCCTGGGCCATACTTGTTGGATGTGATTGTACCTCATCAGGA
Read_8          TCAAAAGATGTTAGACACTCCTGGGCCATACTTGTTGGATGTGATTGTACCTCATCAGGA
Read_9          TCAAAAGATGTTAGACACTCCTGGGCCATACTTGTTGGATGTGATTGTACCTCATCAGGA
Read_10         TCAAAAGATGTTAGACACTCCTGGGCCATACTTGTTGGATGTGATTGTACCTCATCAGGA
Read_11         TCAAAAGATGTTAGACACTCCTGGGCCATACTTGTTGGATGTGATTGTACCTCATCAGGA
Read_12         TCAAAAGATGTTAGACACTCCTGGGCCATACTTGTTGGATGTGATTGTACCTCATCAGGA
Read_13         TCAAAAGATGTTAGACACTCCTGGGCCATACTTGTTGGATGTGATTGTACCTCATCAGGA
Read_14         TCAAAAGATTTTAGACACTCCTGGGCCATACTTGTTGGATGTGATTGTACCTCATCAGGA
Read_15         TCAAAAGATGTTAGACACTCCTGGGCCATACTTGTTGGATGTGATTGTACCTC-------
Read_16         TCAAAAGATGTTAGACACTCCTGGGCCATACTTGTTGGATGTGATTGTACCTCATCAGGA
Read_17         TCAAAAGATGTTAGACACTCCTGGGCCATACTTGTTGGATGTGATTGTACCTCATCAGGA
Read_18         TCAAAAGATGTTAGACACTCCTGGGCCATACTTGTTGGATGTGATTGTACCTCATCAGGA
Read_19         TCAAAAGATGTTAGACACTCCTGGGCCATACTTGTTGGATGTGATTGTACCTCATCAGGA
Read_20         TCAAAAGATGTTAGACACTCCTGGGCCATACTTGTTGGATGTGATTGTACCTCATCAGGA
Read_21         CCAAAAGATGTTAGACACTCCTGGGCCATACTTGTTGGATGTGATTGTACCTCATCAGGA
Read_22         TCAAAAGATGTTAGACACTCCTGGGCCATACTTGTTGGATGTGATTGTACCTCATCAGGA
Read_23         TCAAAAGATGTTAGACACTCCTGGGCCATACTTGTTGGATGTGATTGTACCTCATCAGGA
Read_24         TCAAAAGATGTTAGACACTCCTGGGCCATACTTGTTGGATGTGATTGTACCTCATCAGGA
Read_25         TCAAAAGATGTTAGACACTCCTGGGCCATACTTGTTGGATGTGATTGTACCTCATCAGGA
Read_26         TCAAAAGATGTTAGACACTCCTGGGCCATACTTGTTGGATGTGATTGTACCTCATCAGGA
```

```
Read_27          TCAAAAGATGTTAGACACTCCTGGGCCATACTTGTTGGATGTGATTGTACCTCATCAGGA
Read_28          TCAAAAGATGTTAGACACTCCTGGGCCATACTTGTTGGATGTGATTGTACCTCATCAGGA
Read_29          TCAAAAGATGTTAGACACTCCTGGGCCATACTTGTTGGATGTGATTGTACCTC-------
                  ******** ********************** * ******************

WT               GCACGTTCTACCTA------------------------------------------TGA-
Read_1           GCACGTTCTACCTA--------------------------------TGAGTTTAATTGAG
Read_2           GCACGTTCTACCTA--------------------------------TGAGTTTAATTGAG
Read_3           GCACGTTCTACCTA--------------------------------TGAGTTTAATTGAG
Read_4           GCACGTTCTACCTATCTTTAATTGAGTTGTCATATGTTAATAACGGTATGTTTAATTGAG
Read_5           GCACGTTCTACCTATGTTTAATTGAGTTGTCATATGTTAATAACGGTATGTTTAATTGAG
Read_6           GCACGTTCTACCTA----------------------------------TGTTTAATTGAG
Read_7           GCACGTTCTACCTA----------------------------------TGTTTAATTGAG
Read_8           GCACGTTCTACCTA----------------------------------TGTTTAATTGAG
Read_9           GCACGTTCTACCT---------------------------------------TACCGTTA
Read_10          GCACGTTCTACCT----------------------------------------ACCGTTA
Read_11          GCACGTTCTACCTA--------------------------------TGAGTTTAATTGAG
Read_12          GCACGTTCTACCTA----------------------------------TGTTTAATTGAG
Read_13          GCACGTTCTACCTA----------------------------------TGTTTAATTGAG
Read_14          GCACGTTCTACCTA----------------------------------TGTTTAATTGAG
Read_15          -------------------------------------------------GTTTAATTGAG
Read_16          GCACGTTCTACCTA----------------------------------TGTTTAATTGAG
Read_17          GCACGTTCTACCTA--------------------------------TGAGTTTAATTGAG
Read_18          GCACGTTCTACCTA--------------------------------TGAGTTTAATTGAG
Read_19          GCACGTTCTACCTATATACCGTTATTAACATATGACAACTCAATTAAAC---TACCGTTA
Read_20          GCACGTTCTACCTATATACCGTTATTAACATATGACAACTCAATTAAAC---TACCGTTA
Read_21          GCACGTTCTACCTA--------------------------------TGAGTTTAATTGAG
Read_22          GCACGTTCTACCTA--------------------------------TGAGTTTAATTGAG
Read_23          GCACGTTCTACCTA--------------------------------TGAGTTTAATTGAG
Read_24          GCACGTTCTACCTA--------------------------------TGAGTTTAATTGAG
Read_25          GCACGTTCTACCTAATTTTAATTGAGTTGTCATATGTTACTAACGGTATGTTTAATTGAG
Read_26          GCACGTTCTACCTATGTTTAATTGAGTTGTCATATGTTAATAACGGTATGTTTAATTGAG
Read_27          GCACGTTCTACCTA--------------------------------TGAGTTTAATTGAG
Read_28          GCACGTTCTACCTA--------------------------------TGAGTTTAATTGAG
Read_29          -------------------------------------------------GTTTAATTGAG

WT               ----------------------TTCC----------------------------------
Read_1           TTGTCATATGTTAATAACGGTTTTCC----------------------------------
Read_2           TTGTCATATGTTAATAACGGTTTTCC----------------------------------
Read_3           TTGTCATATGTTAATAACGGTTTTCC----------------------------------
Read_4           TTGTCATATGTTAATAACGGTATACCGTTATTAACATATGATAACGCAATTAAACATTCC
Read_5           TTGTCATATGTTAATAACGGTATACCGTTATTAACATATGACAACTCAATTAAACATTCC
Read_6           TTGTCATATGTTAATAACGGTATTCC----------------------------------
Read_7           TTGTCATATGTTAATAACGGTATTCC----------------------------------
Read_8           TTGTCATATGTTAATAACGGTATTCC----------------------------------
Read_9           TTAACATATGACAACTCAATTAAAC-----------------------------------
Read_10          TTAACATATGACAACTCAATTAAAC-----------------------------GATTCC
Read_11          TTGTCATATGTTAATAACGGTTTTCC----------------------------------
Read_12          TTGTCATATGTTAATAACGGTATTCC----------------------------------
Read_13          TTGTCATATGTTAATAACGGTATTCC----------------------------------
Read_14          TTGTCATATGTTAATAACGGTATTCC----------------------------------
Read_15          TTGTCATATGTTAATAACGGTAT-------------------------------------
Read_16          TTGTCATATGTTAATAACGGTATTCC----------------------------------
Read_17          TTGTCATATGTTAATAACGGTTTTCC----------------------------------
Read_18          TTGTCATATGTTAATAACGGTTTTCC----------------------------------
Read_19          TTAACATATGACAACTCAATTAAAC-----------------------------------
Read_20          TTAACATATGACAACTCAATTAAAC-----------------------------------
Read_21          TTGTCATATGTTAATAACGGTTTTCC----------------------------------
Read_22          TTGTCATATGTTAATAACGGTTTTCC----------------------------------
Read_23          TTGTCATATGTTAATAACGGTTTTCC----------------------------------
Read_24          TTGTCATATGTTAATAACGGTTTTCC----------------------------------
Read_25          TTGTCATATGTTAATAACGGTATTCCGTTATTAACATATGAAAACACAATTATACATTCC
Read_26          TTGTCATATGTTAATAACGGTATACCGTTATTAACATATGACAACTCAATTAAACATTCC
Read_27          TTGTCATATGTTAATAACGGTTTTCC----------------------------------
Read_28          TTGTCATATGTTAATAACGGTTTTCC----------------------------------
Read_29          TTGTCATATGTTAATAACGGTAT-------------------------------------

WT               CAGCGGCGGTGCTTTCAAAGATGTGATCACAGAGGGTGATGGGAGACGTTCCTATTGACT
Read_1           CAGCGGCGGTGCTTTCAAAGATGTGATCACAGAGGGTGATGGGAGACGTTCCTATTGACT
```

```
Read_2          CAGCGGCGGTGCTTTCAAAGATGTGATCACAGAGGGTGATGGGAGACGTTCCTATTGACT
Read_3          CAGCGGCGGTGCTTTCAAAGATGTGATCACAGAGGGTGATGGGAGACGTTCCTATTGACT
Read_4          CAGCGGCGGTGCTTTCAAAGATGTGATCACAGAGGGTGATGGGAGACGTTCCTATTGACT
Read_5          CAGCGGCGGTGCTTTCAAAGATGTGATCACAGAGGGTGATGGGAGACGTTCCTATTGACT
Read_6          CAGCGGCGGTGCTTTCAAAGATGTGATCACAGAGGGTGATGGGAGACGTTCCTATTGACT
Read_7          CAGCGGCGGTGCTTTCAAAGATGTGATCACAGAGGGTGATGGGAGACGTTCCTATTGACT
Read_8          CAGCGGCGGTGCTTTCAAAGATGTGATCACAGAGGGTGATGGGAGACGTTCCTATTGACT
Read_9          -AGCGGCGGTGCTTTCAAAGATGTGATCACAGAGGGTGATGGGAGACGTTCCTATTGACT
Read_10         CAGCGGCGGTGCTTTCAAAGATGTGATCACAGAGGGTGATGGGAGACGTTCCTATTGACT
Read_11         CAGCGGCGGTGCTTTCAAAGATGTGATCACAGAGGGTGATGGGAGACGTTCCTATTGACT
Read_12         CAGCGGCGGTGCTTTCAAAGATGTGATCACAGAGGGTGATGGGAGACGTTCCTATTGACT
Read_13         CAGCGGCGGTGCTTTCAAAGATGTGATCACAGAGGGTGATGGGAGACGTTCCTATTGACT
Read_14         CAGCGGCGGTGCTTTCAAAGATGTGATCACAGAGGGTGATGGGAGACGTTCCTATTGACT
Read_15         -------------------------ATCACAGAGGGTGATGGGAGACGTTCCTATTGACT
Read_16         CAGCGGCGGTGCTTTCAAAGATGTGATCACAGAGGGTGATGGGAGACGTTCCTATTGACT
Read_17         CAGCGGCGGTGCTTTCAAAGATGTGATCACAGAGGGTGATGGGAGACGTTCCTATTGACT
Read_18         CAGCGGCGGTGCTTTCAAAGATGTGATCACAGAGGGTGATGGGAGACGTTCCTATTGACT
Read_19         CAGCGGCGGTGCTTTCAAAGATGTGATCACAGAGGGTGATGGGAGACGTTCCTATTGACT
Read_20         CAGCGGCGGTGCTTTCAAAGATGTGATCACAGAGGGTGATGGGAGACGTTCCTATTGACT
Read_21         CAGCGGCGGTGCTTTCAAAGATGTGATCACAGAGGGTGATGGGAGACGTTCCTATTGACT
Read_22         CAGCGGCGGTGCTTTCAAAGATGTGATCACAGAGGGTGATGGGAGACGTTCCTATTGACT
Read_23         CAGCGGCGGTGCTTTCAAAGATGTGATCACAGAGGGTGATGGGAGACGTTCCTATTGACT
Read_24         CAGCGGCGGTGCTTTCAAAGATGTGATCACAGAGGGTGATGGGAGACGTTCCTATTGACT
Read_25         CAGCGGCGGTGCTTTCAAAGATGTGATCACAGAGGGTGATGGGAGACGTTCCTATTGACT
Read_26         CAGCGGCGGTGCTTTCAAAGATGTGATCACAGAGGGTGATGGGAGACGTTCCTATTGACT
Read_27         CAGCGGCGGTGCTTTCAAAGATGTGATCACAGAGGGTGATGGGAGACGTTCCTATTGACT
Read_28         CAGCGGCGGTGCTTTCAAAGATGTGATCACAGAGGGTGATGGGAGACGTTCCTATTGACT
Read_29         -------------------------ATCACAGAGGGTGATGGGAGACGTTCCTATTGACT
                                         ***********************************
```

TALENs+ODN 1-2

```
WT              AAAGATGTTAGACACTCCTGGGCCATACTTGTTGGATGTGATTGTACCTCATCAGGAGCA
Read_1          AAAGATGTTAGACACTCCTGGGCCATACTTGTTGGATGTGATTGTACCTCATCAGGAGCA
Read_2          AAAGATGTTAGACACTCCTGGGCCATACTTGTTGGATGTGATTGTACCTCATCAGGAGCA
Read_3          AAAGATGTTAGACACTCCTGGGCCATACTTGTTGGATGTGATTGTACCTCATCAGGAGCA
Read_4          AAAGATGTTAGACACTCCTGGGCCATACTTGTTGGATGTGATTGTACCTCATCAGGAGCA
Read_5          AAAGATGTTAGACACTCCTGGGCCATACTTGTTGGATGTGATTGTACCTCATCAGGAGCA
Read_6          AAAGATGTTAGACACTCCTGGGCCATACTTGTTGGATGTGATTGTACCTCATCAGGAGCA
Read_7          AAAGATGTTAGACACTCCTGGGCCATACTTGTTGGATGTGATTGTACCTCATCAGGAGCA
Read_8          AAAGATGTTAGACACTCCTGGGCCATACTTGTTGGATGTGATTGTACCTCATCAGGAGCA
Read_9          AAAGATGTTAGACACTCCTGGGCCATACTTGTTGGATGTGATTGTACCTCATCAGGAGCA
Read_10         AAAGATGTTAGACACTCCTGGGCCATACTTGTTGGATGTGATTGTACCTCATCAGGAGCA
Read_11         AAAGATGTTAGACACTCCTGGGCCATACTTGTTGGATGTGATTGTACCTCATCAGGAGCA
Read_12         AAAGATGTTAGACACTCCTGGGCCATACTTGTTGGATGTGATTGTACCTCATCAGGAGCA
                ************************************************************

WT              CGTTCT----ACCTA----------------TGA--------------------------
Read_1          CGTTCTACCTGTTTAATTGAGTTGTCATATGTTAATAACGGTAT----------------
Read_2          CGTTCTACCTGTTTAATTGAGTTGTCATATGTTAATAACGGTAT----ATACCGTTATTA
Read_3          CGTTCTACCTGTTTAATTGAGTTGTCATATGTTAATAACGGTAT----ATACCGTTATTA
Read_4          CGTTCTACCTATTTAATTGAGTTGTCATATGTTAATAACGGTAT----ATACCGTTTTTA
Read_5          CGTTCTACCTGTTTAATTGAGTTGTCATATGTTAATAACGGTAT----ATACCGTTATTA
Read_6          CGTTCTACCTGTTTAATTGAGTTGTCATATGTTAATAACGGTAT----------------
Read_7          CGTTCTACCTGTTTAATTGAGTTGTCATATGTTAATAACGGTAT----------------
Read_8          CGTTCT----GTTTAATTGAGTTGTCATATGTTAATAACGGTA-----------------
Read_9          CGTTCT----ACCTAATACCGTTATTACAAATGACAACTCAATTAAACATACCGTTATTA
Read_10         CGTTCTACCTGTTTAATTGAGTTGTCATATGTTAATAACGGTAT----------------
Read_11         CGTTCT----GTTTAATTGAGTTGTCATATGTTAATAACGGTA-----------------
Read_12         CGTTCT----GTTTAATTGAGTTGTCATATGTTAATAACGGTA-----------------
                ******       **                **

WT              --------------------------TTCCCAGCGGCGGTGCTTTCAAAGATGTGATCAC
Read_1          --------------------------TTCCCAGCGGCGGTGCTTTCAAAGATGTGATCAC
Read_2          ACATATGACAACTCAATTAAACATGATTCCCAGCGGCGGTGCTTTCAAAGATGTGATCAC
Read_3          ACATATGACAACTCAATTAAACATGATTCCCAGCGGCGGTGCTTTCAAAGATGTGATCAC
Read_4          ACATATGACAACTCAATTAAACATGATTCCCAGCGGCGGTGCTTTCAAAGATGTGATCAC
Read_5          ACATATGACAACTCAATTAAACATGATTCCCAGCGGCGGTGCTTTCAAAGATGTGATCAC
Read_6          --------------------------TTCCCAGCGGCGGTGCTTTCAAAGATGTGATCAC
Read_7          --------------------------TTCCCAGCGGCGGTGCTTTCAAAGATGTGATCAC
Read_8          --------------------------------GCGGCGGTGCTTTCAAAGATGTGATCAC
```

```
Read_9          ACATATGACAACTCAATTAAACA---TTCCCAGCGGCGGTGCTTTCAAAGATGTGATCAC
Read_10         --------------------------TTCCCAGCGGCGGTGCTTTCAAAGATGTGATCAC
Read_11         --------------------------------GCGGCGGTGCTTTCAAAGATGTGATCAC
Read_12         --------------------------------GCGGCGGTGCTTTCAAAGATGTGATCAC
                                                ****************************
```

TALENs+ODN 1-3

```
WT              GCACGTTCTACCTATG--------------------------------------------
Read_1          GCACGTTCTACCT--GTTTAATTGAGTTGTCATATGTTAATAACGGTAT---------
Read_2          GCACGTTCTACCTATACCGTTATTAACATATGACAACTCAATTAAACATACCGTTATTAA
Read_3          GCACGTTCTACCTATGTTTAATTGAGTTGTCATATGTTAATAACGGTAT--TTTAATTGA
Read_4          GCACGTTCTACCT--GTTTAATTGAGTTGTCATATGTTAATAACGGTAT-----------
Read_5          GCACGTTCTACCT--GTTTAATTGAGTTGTCATATGTTAATAACGGTAT-----------
Read_6          GCACGTTCTACCT--GTTTAATTGAGTTGTCATATGTTAATAACGGTAT-----------
Read_7          GCACGTTCTACCT--GTTTAATTGAGTTGTCATATGTTAATAACGGTAT-----------
Read_8          GCACGTTCTACCTATACCGTTATTAACATATAACAACTCAATTAAACATACCGTTATTAA
Read_9          GCACGTTCTACCTATACCGTTATTAACATATGACAACTCAATTAAACATACCGTTATTAA
Read_10         GCACGTTCTACCTATACCGTTATTAACATATGACAACTCAATTAAACATACCGTTATTAA
Read_11         GCACGTTCTACCTATACCGTTATTAACATATGACAACTCAATTAAACATACCGTTATTAA
Read_12         GCACGTTCTACCT--GTTTAATTGAGTTGTCATATGTTAATAACGGTAT-----------
Read_13         GCACGTTCTACCTATGTTTAATTGAGTTGTCATATGTTAATAACGGTAT--TTTAATTGA
Read_14         GCACGTTCTACCTATGTTTAATTGAGTTGTCATATGTTAATAACGGTAT--TTTAATTGA
Read_15         GCACGTTCTACCTATACCGTTATTAACATATGACAACTCAATTAAACATACCGTTATGAA
Read_16         GCACGTTCTACCTATACCGTTATTAACATATGACAACTCAATTAAACATACCGTTATTAA
Read_17         GCACGTTCTACCTATACCGTTATTAACATATGACAACTCAATTAAACATACCGTTATTAA
Read_18         GCACGTTCTACCT--GTTTAATTGAGTTGTCATATGTTAATAACGGTAT-----------
Read_19         GCACGTTCTACCTATGTTTAATTGAGTTGTCATATGTTAATAACGGTAT--TTTAATTGA
Read_20         GCACGTTCTACCTATGTTTAATTGAGTTGTCATATGTTAATAACGGTAT--TTTAATTGA
Read_21         GCACGTTCTACCTATACCGTTATTAACATATGACAACTCAATTAAACATACCGTTATTAA
Read_22         GCACGTTCTACCTATACCGTTATTAACATATGACAACTCAATTAAACATACCGTTATTAA
Read_23         GCACGTTCTACCT--GTTTAATTGAGTTGTCATATGTTAATAACGGTAT-----------
Read_24         GCACGTTCTACCT--GTTTAATTGAGTTGTCATATGTTAATAACGGTAT-----------
Read_25         GCACGTTCTACCT--GTTTAATTGAGTTGTCATATGTTAATAACGGTAT-----------
                *************

WT              -------------------------ATTCCCAGCGGCGGTGCTTTCAAAGATGTGATCAC
Read_1          -------------------------------------GGTGCTTTCAAAGATGTGATCAC
Read_2          CATATGACAACTCAATTAAAC------TCCCAGCGGCGGTGCTTTCAAAGATGTGATCAC
Read_3          GTTGTCATATGTTAATAACGGTATGATTCCCAGCGGCGGTGCTTTCAAAGATGTGATCAC
Read_4          -------------------------------------GGTGCTTTCAAAGATGTGATCAC
Read_5          -------------------------------------GGTGCTTTCAAAGATGTGATCAC
Read_6          -------------------------------------GGTGCTTTCAAAGATGTGATCAC
Read_7          -------------------------------------GGTGCTTTCAAAGATGTGATCAC
Read_8          CATATGACAACTCAATTAAAC------TCCCAGCGGCGGTGCTTTCAAAGATGTGATCAC
Read_9          CATATGACAACTCAATTAAAC------TCCCAGCGGCGGTGCTTTCAAAGATGTGATCAC
Read_10         CATATGACAACTCAATTAAAC------TCCCAGCGGCGGTGCTTTCAAAGATGTGATCAC
Read_11         CATATGAAAACTCAATTAAAC------TCCCAGCGGCGGTGCTTTCAAAGATGTGATCAC
Read_12         -------------------------------------GGTGCTTTCAAAGATGTGATCAC
Read_13         GTTGTCATATGTTAATAACGGTATGATTCCCAGCGGCGGTGCTTTCAAAGATGTGATCAC
Read_14         GTTGTCATATGTTAATAACGGTATGATTCCCAGCGGCGGTGCTTTCAAAGATGTGATCAC
Read_15         CATATGACAACTGAATTAAAC------TCCCAGCGGCGGTGCTTTCAAAGATGTGATCAC
Read_16         CATATGACAACTCAATTAAAC------TCCCAGCGGCGGTGCTTTCAAAGATGTGATCAC
Read_17         CATATGACAACTCAATTAAAC------TCCCAGCGGCGGTGCTTTCAAAGATGTGATCAC
Read_18         -------------------------------------GGTGCTTTCAAAGATGTGATCAC
Read_19         GTTGTCATATGTTAATAACGGTATGATTCCCAGCGGCGGTGCTTTCAAAGATGTGATCAC
Read_20         GTTGTCATATGTTAATAACGGTATGATTCCCAGCGGCGGTGCTTTCAAAGATGTGATCAC
Read_21         CATATGACAACTCAATTAAAC------TCCCAGCGGCGGTGCTTTCAAAGATGTGATCAC
Read_22         CATATGACAACTCAATTAAAC------TCCCAGCGGCGGTGCTTTCAAAGATGTGATCAC
Read_23         -------------------------------------GGTGCTTTCAAAGATGTGATCAC
Read_24         -------------------------------------GGTGCTTTCAAAGATGTGATCAC
Read_25         -------------------------------------GGTGCTTTCAAAGATGTGATCAC
                                                     ***********************
```

TALENs+ODN 2-1

```
WT              GCACGTTCTACCTATG--------------------------------------------
Read_1          GCACGTTCTACCT---------------------------ATACCGTTATTAACATATGA
Read_2          GCACGTTCTACCT---------------------------ATACCGTTATTAACATATGA
Read_3          GCACGTTCTACCT------------------------------GTTTAATTGAGTTGTCA
Read_4          GCACGTTCTACCTATGTTTAATTGAGTTGTCATATGTTAATAACGGTAGTTTAATTGAGT
```

```
Read_5        GCACGTTCTACCT------------------------------GTTTAATTGAGTTGTCA
Read_6        GCACGTTCTACCT------------------------------GTTTAATTGAGTTGTCA
Read_7        GCACGTTCTACCTAT------------------------------TTAATTGAGTTGTCA
Read_8        GCACGTTCTACCTATGTTTAATTGAGTTGTCATATGTTAATAACGGTAGTTTAATTGAGT
Read_9        GCACGTTCTACCTATG--------------------------------------------
Read_10       GCACGTTCTACCTATGAT------------------------------------------
Read_11       GCACGTTCTACCTATGAT------------------------------------------
Read_12       GCACGTTCTACC-AT---------------------------------------------
Read_13       GCACGTTCTACC-AT---------------------------------------------
Read_14       GCACGGTCTACCT---------------------------ATACCGTTATTAACATATGA
Read_15       GCACGTTCTACCT---------------------------ATACCGTTATTAACATATGA
Read_16       GCACGTTCTACCTAT----------------------------GTTTAATTGAGTTGTCA
Read_17       GCACGTTCTACCTAT----------------------------GTTTAATTGAGTTGTCA
Read_18       GCACGTTCTACCTATG--------------------------------------------
Read_19       GCACGTTCTACCTATGA--------------------------GTTTAATTGAGTTGTCA
Read_20       GCACGTTCTACCTATG--------------------------------------------
Read_21       GCACGTTCTACCTATG--------------------------------------------
Read_22       GCACGTTCTACCT------------------------------GTTTAATTGAGTTGTCA
Read_23       GCACGTTCTACCT------------------------------GTTTAATTGAGTTGTCA
Read_24       GCACGTTCTACCT------------------------------GTTTAATTGAGTTGTCA
Read_25       GCACGTTCTACCT------------------------------GTTTAATTGAGTTGTCA
Read_26       GCACGTTCTACCTATGA--------------------------GTTTAATTGAGTTGTCA
Read_27       GCACGTTCTACCT------------------------------GTTTAATTGAGTTGTCA
Read_28       GCACGTTCTACCT------------------------------GTTTAATTGAGTTGTCA
Read_29       GCACGTTCTACCTATG--------------------------------------------
Read_30       GCACGTTCTACCTATG--------------------------------------------
Read_31       GCACGTTCTACCTATGA--------------------------GTTTAATTGAGTTGTCA
Read_32       GCACGTTCTACCTATG------------------------ATACCGTTATTAACATATGA
Read_33       GCACGTTCTACCTATG------------------------ATACCGTTATTAACATATGA
              ***** ******  *

WT            -----------------------------------------------------ATTCCCA
Read_1        CAACTCAATTAAACGTTTAATT----GAGTTGTCATATGTTAATAACGGTATGATTCCCA
Read_2        CAACTCAATTAAACGTTTAATT----GAGTTGTCATATGTTAATAACGGTATGATTCCCA
Read_3        TATGTTAATAACGGTAT--------------------------------------TCCCA
Read_4        TGTCATATGTTAATAACGGTTTACCGTTATTAACATATGACAACTCAATTAAAC------
Read_5        TATGTTAATAACGGTAT--------------------------------------TCCCA
Read_6        TATGTTAATAACGGTAT--------------------------------------TCCCA
Read_7        TATGTTAATAACGGTAT--------------------------------------TCCCA
Read_8        TGTCATATGTTAATAACGGTTTACCGTTATTAACATATGACAACTCAATTAAAC------
Read_9        ---------------------TACCGTTATTAACATATGACAACTCAATTAAACTTCCCA
Read_10       ----------------------ACCGTTATTAACATATGACAACTCAATTAAAC------
Read_11       ----------------------ACCGTTATTAACATATGACAACTCAATTAAAC------
Read_12       ----------------------ACCGTTATTAACATATGACAACTCAATTAAAC------
Read_13       ----------------------ACCGTTATTAACATATGACAACTCAATTAAAC------
Read_14       CAACTCAATTAAAC---GTTTAATTGAGTTGTCATATGTTAATAACGGTAT-GATTCCCA
Read_15       CAACTCAATTAAAC---GTTTAATTGAGTTGTCATATGTTAATAACGGTAT-GATTCCCA
Read_16       TATGTTAATAACGGTA-GTTTAATTGAGTTGTCATATGCTAATAACGGTAT--ATTCCCA
Read_17       TATGTTAATAACGGTA-GTTTAATTGAGTTGTCATATGTTAATAACGGTAT--ATTCCCA
Read_18       ---------------------TACCGTTATTAACATATGACAACTCAATTAAACTTCCCA
Read_19       TATGTTAATAACGGTA----ATACCGTTATTAACATATGACAACTCAATTAAACTTCCCA
Read_20       ---------------------TACCGTTATTAACATATGACAACTCAATTAAACTTCCCA
Read_21       ---------------------TACCGTTATTAACATATGACAACTCAATTAAACTTCCCA
Read_22       TATGTTAATAACGGTAT--------------------------------------TCCCA
Read_23       TATGTTAATAACGGTAT-----------------------------------GATTCCCA
Read_24       TATGTTAATAACGGTAT-----------------------------------GATTCCCA
Read_25       TATGTTAATAACGGTAT-----------------------------------GATTCCCA
Read_26       TATGTTAATAACGGTAATACCGTTATTAACATATGACAACTCAATTAAAC----TTCCCA
Read_27       TATGTTAATAACGGTAT-----------------------------------GATTCCCA
Read_28       TATGTTAATAACGGTAT-----------------------------------GATTCCCA
Read_29       ----------------ATACCGTTATTAACATATGACAACTCAATTAAAC----------
Read_30       ----------------ATACCGTTATTAACATATGACAACTCAATTAAAC----------
Read_31       TATGTTAATAACGGTAATACCGTTATTAACATATGACAACTCAATTAAAC----TTCCCA
Read_32       CAACTCAATTAAAC---GTTTAATTGAGTTGTCATATGTTAATAACGGTAT--ATTCCCA
Read_33       CAACTCAATTAAAC---GTTTAATTGAGTTGTCATATGTTAATAACGGTAT--ATTCCCA

WT            GCGGCGGTGCTTTCAAAGATGTGATCACAGAGGGTGATGGGAGACGTTCCTATTGACTTT
Read_1        GCGGCGGTGCTTTCAAAGATGTGATCACAGAGGGTGATGGGAGACGTTCCTATTGACTTT
Read_2        GCGGCGGTGCTTTCAAAGATGTGATCACAGAGGGTGATGGGAGACGTTCCTATTGACTTT
Read_3        GCGGCGGTGCTTTCAAAGATGTGATCACAGAGGGTGATGGGAGACGTTCCTATTGACTTT
```

```
Read_4          -----GGTGCTTTCAAAGATGTGATCACAGAGGGTGATGGGAGACGTTCCTATTGACTTT
Read_5          GCGGCGGTGCTTTCAAAGATGTGATCACAGAGGGTGATGGGAGACGTTCCTATTGACTTT
Read_6          GCGGCGGTGCTTTCAAAGATGTGATCACAGAGGGTGATGGGAGACGTTCCTATTGACTTT
Read_7          GCGGCGGTGCTTTCAAAGATGTGATCACAGAGGGTGATGGGAGACGTTCCTATTGACTTT
Read_8          -----GGTGCTTTCAAAGATGTGATCACAGAGGGTGATGGGAGACGTTCCTATTGACTTT
Read_9          GCGGCGGTGCTTTCAAAGATGTGATCACAGAGGGTGATGGGAGACGTTCCTATTGACTTT
Read_10         -----GGTGCTTTCAAAGATGTGATCACAGAGGGTGATGGGAGACGTTCCTATTGACTTT
Read_11         -----GGTGCTTTCAAAGATGTGATCACAGAGGGTGATGGGAGACGTTCCTATTGACTTT
Read_12         --GGCGGTGCTTTCAAAGATGTGATCACAGAGGGTGATGGGAGACGTTCCTATTGACTTT
Read_13         --GGCGGTGCTTTCAAAGATGTGATCACAGAGGGTGATGGGAGACGTTCCTATTGACTTT
Read_14         GCGGCGGTGCTTTCAAAGATGTGATCACAGAGGGTGATGGGAGACGTTCCTAGTGACGTG
Read_15         GCGGCGGTGCTTTCAAAGATGTGATCACAGAGGGTGATGGGAGACGTTCCTATTGACTTT
Read_16         GCGGCGGTGCTTTCAAAGATGTGATCACAGAGGGTGATGGGAGCCGTTCCTATTGACTTT
Read_17         GCGGCGGTGCTTTCAAAGATGTGATCACAGAGGGTGATGGGAGACGTTCCTATTGACTTT
Read_18         GCGGCGGTGCTTTCAAAGATGTGATCACAGAGGGTGATGGGAGACGTTCCTATTGACTTT
Read_19         GCGGCGGTGCTTTCAAAGATGTGATCACAGAGGGTGATGGGAGACGTTCCTATTGACTTT
Read_20         GCGGCGGTGCTTTCAAAGATGTGATCACAGAGGGTGATGGGAGACGTTCCTATTGACTTT
Read_21         GCGGCGGTGCTTTCAAAGATGTGATCACAGAGGGTGATGGGAGACGTTCCTATTGACTTT
Read_22         GCGGCGGTGCTTTCAAAGATGTGATCACAGAGGGTGATGGGAGACGTTCCTATTGACTTT
Read_23         GCGGCGGTGCTTTCAAAGATGTGATCACAGAGGGTGATGGGAGACGTTCCTATTGACTTT
Read_24         GCGGCGGTGCTTTCAAAGATGTGATCACAGAGGGTGATGGGAGACGTTCCTATTGACTTT
Read_25         GCGGCGGTGCTTTCAAAGATGTGATCACAGAGGGTGATGGGAGACGTTCCTATTGACTTT
Read_26         GCGGCGGTGCTTTCAAAGATGTGATCACAGAGGGTGATGGGAGACGTTCCTATTGACTTT
Read_27         GCGGCGGTGCTTTCAAAGATGTGATCACAGAGGGTGATGGGAGACGTTCCTATTGACTTT
Read_28         GCGGCGGTGCTTTCAAAGATGTGATCACAGAGGGTGATGGGAGACGTTCCTATTGACTTT
Read_29         -----GGTGCTTTCAAAGATGTGATCACAGAGGGTGATGGGAGACGTTCCTATTGACTTT
Read_30         -----GGTGCTTTCAAAGATGTGATCACAGAGGGTGATGGGAGACGTTCCTATTGACTTT
Read_31         GCGGCGGTGCTTTCAAAGATGTGATCACAGAGGGTGATGGGAGACGTTCCTATTGACTTT
Read_32         GCGGCGGTGCTTTCAAAGATGTGATCACAGAGGGTGATGGGAGACGTTCCTATTGACTTT
Read_33         GCGGCGGTGCTTTCAAAGATGTGATCACAGAGGGTGATGGGAGACGTTCCTATTGACTTT
                     ************************************** ******** **** *
```

TALENs+ODN 2-2

```
WT              GCACGTTCTACCTATGAT------------------------------------------
Read_1          GCACGTTCTACCTATACCGTTATTAACATATGACAACTCAATTAAAC-TACCGTTATCAA
Read_2          GCACGTTCTACCTATACCGTTATTAACATATGACAACTCAATTAAAC-TACCGTTATCAA
Read_3          GCACGTTCTACCTATACCGTTATTAACATATGACAACTCAATTAAAC-TACCGTTATCAA
Read_4          GCACGTTCT-------GTTTAATTGAGTTGTCATATGTTAATAACGGTAT----------
Read_5          GCACGTTCTACCTATACCGTTATTAACATATGACAACTCAATTAATC---GAT-------
Read_6          GCACGTTCTACCTATACCGTTATTAACATATGACAACTCAATTAAAC---GAT-------
Read_7          GCACGTTCTACCTAT-GTTTAATTGAGTTGTCATATGTTAATAACGGTAT----------
Read_8          GCACGTTCTACCTATGGTTTAATTGAGTTGTCATATGTTAATAACGGTATGATTCTCAT
Read_9          GCACGTT------------TAATTGAGTTGTCATATGTTAATAACGGTAT----------
Read_10         GCACGTT------------TAATTGAGTTGTCATATGTTAATAACGGTAT----------
Read_11         GCACGTT------------TAATTGAGTTGTCATATGTTAATAACGGTAT----------
Read_12         GCACGTT------------TAATTGAGTTGTCATATGTTAATAACGGTAT----------
Read_13         GCACGTT-----------------------------------------------------
Read_14         GCACGTT---------GTTTAATTGAGTTGTCATATGTTAATAACGGTAT----------
Read_15         GCACGTTCTA------GTTTAATTTAGTTGTCATATGTTAATAACGT-AATGTTAATTTG
Read_16         GCACGTTCTACCTATACCGTTATTAACATATGACAACTCAATTAAAC-TACCGTTATTAA
Read_17         GCACGTTCT-------GTTTAATTGAGTTGTCATATGTTAATAACGGTAT----------
Read_18         GCACGTTCT-------GTTTAATTGAGTTGTCATATGTTAATAACGGTAT----------
Read_19         GCACGTTCT-------GTTTAATTGAGTTGTCATATGTTAATAACGGTAT----------
Read_20         GCACGTTCTACCTATACCGTTATTAACATATGACAACTCAATTAAAC-GAT---------
Read_21         GCACGTTCTACCTAT-GTTTAATTGAGTTGTCATATGTTAATAACGGTAT----------
Read_22         GCACGTTCTACCTAT-GTTTAATTGAGTTGTCATATGTTAATAACGGTAT----------
Read_23         GCACGTTCTACCTATACCGTTATTAACATATGACAACTCAATTAAAC-T-----------
Read_24         GCACGTTCTA------GTTTAATTGAGTTGTCATATGTTAATAACGGTAT----------
Read_25         GCACGTT------------TAATTGAGTTGTCATATGTTAATAACGGTAT----------
Read_26         GCACGTT------------TAATTGAGTTGTCATATGTTAATAACGGTAT----------
Read_27         GCACGTT------------TAATTGAGTTGTCATATGTTAATAACGGTAT----------
Read_28         GCACGTTCTACCTACACCGTTATTAACATATGACAACTCAATTAAACATACCGTTATTAA
Read_29         GCACGTTCTACCTAAACCGTTATTAACATATGACAACTCAATTAAACATTCCGTTATTAA
Read_30         GCACGTTCTACCTATACCGTTATTAACATATGACAACTAAATTAAACATACCGTTATTAA
Read_31         GCACGTT---------GTTTAATTGAGTTGTCATATGTTAATAACGGTAT----------
Read_32         GCACGTT---------GTTTAATTGAGTTGTCATATGTTAATAACGGTAT----------
Read_33         GCACGTT---------GTTTAATTGAGTTGTCATATGTTAATAACGGTATG--------
Read_34         GCACGTTCTACCTATACCGTTATTAACATATGACAACTCAATTAAAC-TACCGTTATTAA
Read_35         GCACGTTCTACCTAT-GTTTAATTGAGTTGTCATATGTTAATAACGGTATATACCGT--
Read_36         GCACGTTCTACCTAT-GTTTAATTGAGTTGTCATATGTTAATAACGGTATATACCGT--
```

```
Read_37         GCACGTTCTACCTAT-GTTTAATTGAGTTGTCATATGTTAATAACGGTATATACCGT--
Read_38         GCACGTTCTACCTATACCGTTATTAACATATGACAACTCAATTAAAC-T-----------
                *******

WT              ------------------------------------------------------------
Read_1          CATATGACAACTCAATTAAACTT-------------------------------------
Read_2          CATATGACAACTCAATTAAACTT-------------------------------------
Read_3          CATATGACAACTCAATTAAACTT-------------------------------------
Read_4          ------------------------------------------------------------
Read_5          ------------------------------------------------------------
Read_6          ------------------------------------------------------------
Read_7          ------------------------------------------------------------
Read_8          AGTGAATTGTTTAGTTAA------------------------------------ACACTT
Read_9          ------------------------------------------------------------
Read_10         ------------------------------------------------------------
Read_11         ------------------------------------------------------------
Read_12         ------------------------------------------------------------
Read_13         ------------------------------------------------------------
Read_14         ------------------------------------------------------------
Read_15         ATTGTTCATTGCTAATAACCGAATCCCATTTTTTCACGCCTTCGGTCCTTCCACAGATTG
Read_16         CATATGACAACTCAATTACACT--------------------------------------
Read_17         ------------------------------------------------------------
Read_18         ------------------------------------------------------------
Read_19         ------------------------------------------------------------
Read_20         ------------------------------------------------------------
Read_21         ------------------------------------------------------------
Read_22         ------------------------------------------------------------
Read_23         ------------------------------------------------------------
Read_24         ------------------------------------------------------------
Read_25         ------------------------------------------------------------
Read_26         ------------------------------------------------------------
Read_27         ------------------------------------------------------------
Read_28         CATATGGCAACTCAATTAAAC---------------------------------------
Read_29         CATATGTCAACTCAATTAAAC---------------------------------------
Read_30         CATATGACAACTCAATTAAAC----------------------------------------
Read_31         ------------------------------------------------------------
Read_32         ------------------------------------------------------------
Read_33         ------------------------------------------------------------
Read_34         CATATGACAACTCAATTAAACT--------------------------------------
Read_35         ------------------------------------------------------------
Read_36         ------------------------------------------------------------
Read_37         ------------------------------------------------------------
Read_38         ------------------------------------------------------------


WT              -----------------------------------------------------------T
Read_1          ----TAATTGAGTTGTCATATGTTAATAACGGTAT-GTTGTCATATGTTAATAACGGTAT
Read_2          ----TAATTGAGTTGTCATATGTTAATAACGGTAT-GTTGTCATATGTTAATAACGGTAT
Read_3          ----TAATTGAGTTGTCATATGTTAATAACGGTAT-GTTGTCATATGTTAATAACGGTAT
Read_4          -GTTTAATTGAGTTGTCATATGTTAATAACGGTAT------------------------T
Read_5          -----------------------------------------------------------T
Read_6          -----------------------------------------------------------T
Read_7          ATACCGTTATTAACATATGACAACTCAATTAATACCGTTATTA--ACATATGACAACTCA
Read_8          TATGTAAATAAATATTACCGATTCAATAACTTATATGTAACAAAAAAATAT----TGATT
Read_9          -----------------------------------------------------------T
Read_10         -----------------------------------------------------------T
Read_11         -----------------------------------------------------------T
Read_12         -----------------------------------------------------------T
Read_13         ---------------GTTTAATTGAGTTGTCATATGTTAATAACGGTAT-----------
Read_14         ------------------------------------------------------------
Read_15         AATAACCAACGGTATGTTTAATTGAGTTGTCATATGTTAATAACGGTATCCTATGATT--
Read_16         -----------------------------------------------------------T
Read_17         ---------------GTTTAATTGAGTTGTCATATGTTAATAACGGTAT----------T
Read_18         ---------------GTTTAATTGAGTTGTCATATGTTAATAACGGTAT----------T
Read_19         ---------------GTTTAATTGAGTTGTCATATGTTAATAACGGTAT----------T
Read_20         -----------------------------------------------------------T
Read_21         ATACCGTTATTAACATATGACAACTCAATTAATACCGTTATTA--ACATATGACAACTCA
Read_22         ATACCGTTATTAACATATGACAACTCAATTAATACCGTTATTA--ACATATGACAACTCA
Read_23         -----------------------------------------------------------T
Read_24         ---------------GTTTAATTGAGTTGTCATATGTTAATAACGGTATCCTATGATT--
Read_25         -----------------------------------------------------------T
```

```
Read_26         -----------------------------------------------------------T
Read_27         -----------------------------------------------------------T
Read_28         ------------------------------------------------------------
Read_29         ------------------------------------------------------------
Read_30         ------------------------------------------------------------
Read_31         ------------------------------------------------------------
Read_32         ------------------------------------------------------------
Read_33         ------------------------------------------------------------
Read_34         -----------------------------------------------------------T
Read_35         -TATTAACATATGACAACTCAATTAA-TACCGTTATTAACATATGACAACTCAATTAAAC
Read_36         -TATTAACATATGACAACTCAATTAA-TACCGTTATTAACATATGACAACTCAATTAAAC
Read_37         -TATTAACATATGACAACTCAATTAA-TACCGTTATTAACATATGACAACTCAATTAAAC
Read_38         -----------------------------------------------------------T


WT              CCCAGCGGCGGTGCTTTCAAAGATGTGATCACAGAGGGTGATGGGAGACGTTCCTATTGA
Read_1          CCCAGCGGCGGTGCTTTCAAAGATGTGATCACAGAGGGTGATGGGAGACGTTCCTATTGA
Read_2          CCCAGCGGCGGTGCTTTCAAAGATGTGATCACAGAGGGTGATGGGAGACGTTCCTATTGA
Read_3          CCCAGCGGCGGTGCTTTCAAAGATGTGATCACAGAGGGTGATGGGAGACGTTCATATTGA
Read_4          CCCAGCGGCGGTGCTTTCAAAGATGTGATCACAGAGGGTGATGGGAGACGTTCCTATTGA
Read_5          CCCAGCGGCGGTGCTTTCAAAGATGTGATCACAGAGGGTGATGGGAGACGTTCCTATTGA
Read_6          CCCAGCGGCGGTGCTTTCAAAGATGTGATCACAGAGGGTGATGGGAGACGTTCCTATTGA
Read_7          ATTAAACGCGGTGCTTTCAAAGATGTGATCACAGAGGGTGATGGGAGACGTTCCTATTGA
Read_8          CCCAGCGGCGGTGCTTTCAAAGATGTGATCACAGAGGGTGATGGGAGACGTTCCTATTGA
Read_9          CCCAGCGGCGGTGCTTTCAAAGATGTGATCACAGAGGGTGATGGGAGACGTTCCTATTGA
Read_10         CCCAGCGGCGGTGCTTTCAAAGATGTGATCACAGAGGGTGATGGGAGACGTTCCTATTGA
Read_11         CCCAGCGGCGGTGCTTTCAAAGATGTGATCACAGAGGGTGATGGGAGACGTTCCTATTGA
Read_12         CCCAGCGGCGGTGCTTTCAAAGATGTGATCACAGAGGGTGATGGGAGACGTTCCTATTGA
Read_13         ------------GCTTTCAAAGATGTGATCACAGAGGGTGATGGGAGACGTTCCTATTGA
Read_14         ------------GCTTTCAAAGATGTGATCACAGAGGGTGATGGGAGACGTTCCTATTGA
Read_15         CCCAGCGGCGGTGCTTTCAAAGATGTGATCACAGAGGGTGATGGGAGACGTTCCTATTGA
Read_16         CCCAGCGGCGGTGCTTTCAAAGATGTGATCACAGAGGGTGATGGGAGACGTTCCTATTGA
Read_17         CCCAGCGGCGGTGCTTTCAAAGATGTGATCACAGAGGGTGATGGGAGACGTTCCTATTGA
Read_18         CCCAGCGGCGGTGCTTTCAAAGATGTGATCACAGAGGGTGATGGGAGACGTTCCTATTGA
Read_19         CCCAGCGGCGGTGCTTTCAAAGATGTGATCACAGAGGGTGATGGGAGACGTTCCTATTGA
Read_20         CCCAGCGGCGGTGCTTTCAAAGATGTGATCACAGAGGGTGATGGGAGACGTTCCTATTGA
Read_21         ATTAAACGCGGTGCTTTCAAAGATGTGATCACAGAGGGTGATGGGAGACGTTCCTATTGA
Read_22         ATTAAACGCGGTGCTTTCAAAGATGTGATCACAGAGGGTGATGGGAGACGTTCCTATTGA
Read_23         CCCAGCGGCGGTGCTTTCAAAGATGTGATCACAGAGGGTGATGGGAGACGTTCCTATTGA
Read_24         CCCAGCGGCGGTGCTTTCAAAGATGTGATCACAGAGGGTGATGGGAGACGTTCCTATTGA
Read_25         CCCAGCGGCGGTGCTTTCAAAGATGTGATCACAGAGGGTGATGGGAGACGTTCCTATTGA
Read_26         CCCAGCGGCGGTGCTTTCAAAGATGTGATCACAGAGGGTGATGGGAGACGTTCCTATTGA
Read_27         CCCAGCGGCGGTGCTTTCAAAGATGTGATCACAGAGGGTGATGGGAGACGTTCCTATTGA
Read_28         ---------GGTGCTTTCAAAGATGTGATCACAGAGGGTGATGGGAGACGTTCCTATTGA
Read_29         ---------GGTGCTTTCAAAGATGTGATCACAGAGGGTGATGGGAGACGTTCCTATTGA
Read_30         ---------GGTGCTTTCAAAGATGTGATCACAGAGGGTGATGGGAGACGTTCTATTGA
Read_31         ------------GCTTTCAAAGATGTGATCACAGAGGGTGATGGGAGACGTTCCTATTGA
Read_32         ------------GCTTTCAAAGATGTGATCACAGAGGGTGATGGGAGACGTTCCTATTGA
Read_33         -------------CTTTCAAAGATGTGATCACAGAGGGTGATGGGAGACGTTCCTATTGA
Read_34         CCCAGCGGCGGTGCTTTCAAAGATGTGATCACAGAGGGTGATGGGAGACGTTCCTATTGA
Read_35         -------GCGGTGCTTTCAAAGATGTGATCACAGAGGGTGATGGGAGACGTTCCTATTGA
Read_36         -------GCGGTGCTTTCAAAGATGTGATCACAGAGGGTGATGGGAGACGTTCCTATTGA
Read_37         -------GCGGTGCTTTCAAAGATGTGATCACAGAGGGTGATGGGAGACGTTCCTATTGA
Read_38         CCCAGCGGCGGTGCTTTCAAAGATGTGATCACAGAGGGTGATGGGAGACGTTCCTATTGA
                             **************************************** ******
```

TALENs+ODN 2-3

```
WT              GCACGTTCTACCTAT---------------------------------------------
Read_1          GCACGTTCTACCT--------------------------------------GTTTAATTG
Read_2          GCACGTTCTACCTA-------------------------------------GTTTAATTG
Read_3          GCACGTTCTACCT--------------------------------------GTTTAATTG
Read_4          GCACGTTCTACCT--------------------------------------GTTTAATTG
Read_5          GCACGTTCTACCTA---ATACCGTTATTAACATATGACAAATCAATTAAACGTTTAATTG
Read_6          GCACGTTCTACCTA---ATACCGTTATTAACATATGACAACTCAATTAAACGTTTAATTG
Read_7          GCACGTTCTACCTA---ATACCGTTATTAACATATGACAAATCAATTAAACGTTTAATTG
Read_8          GCACATTCTACCTA-------------------------------------GTTTAATTG
Read_9          GCACGTTCTACCTA---ATACCGTTATTAACATATGACAACTCAATTAAAC---------
Read_10         GCACGTTCTACCTA---ATACCGTTATTAACATATGACAACTCAATTAAAC---------
Read_11         GCACGTTCTACCT--------------------------------------GTTTAATTG
Read_12         GCACGTTCTACCTA-------------------------------------GTTTAATTG
```

```
Read_13          GCACGTTCTACCTA-------------------------------------GTTTAATTG
Read_14          GCACGTTCTACCTATGA----------------------------------GTTTAATTG
Read_15          GCACGTTCTACCTA-------------------------------------GTTTAATTG
Read_16          GCACGTTCTACCTA-------------------------------------GTTTAATTG
Read_17          GCACGTTCTACCTA---ATACCGTTATTAACATATGACAACTCAATTAAACGTTTAATTG
Read_18          GCACGTTCTACCTA---ATACCGTTATTAACATATGACAACTCAATTAAACGTTTAATTG
Read_19          GCACGTTCTACCTA---ATACCGTTATTAACATATGACAACTCACTTAAACGTTTAATTG
Read_20          GCAC-----------------------------------------------GTTTAATTG
Read_21          GCAC-----------------------------------------------GTTTAATTG
Read_22          GCACGTTCTACCTA-------------------------------------GTTTAATTG
Read_23          GCACGTTCTACCTATGA----------------------------------GTTTAATTG
Read_24          GCACGTT----CTA----------------------------------ATACCGTTATTA
Read_25          GCACGTTCTACCTATGA----------------------------------GTTTAATTG
Read_26          GCACGTTCTACCTA-------------------------------------GTTTAATTG
Read_27          GCACGTTCTACCTA----------------------------------ATACCGTTATTA
Read_28          GCACGTTCTACCTA----------------------------------ATACCGTTATTA
Read_29          GCACGTTCTACCTA----------------------------------ATACCGTTATTA
Read_30          GCACGTTCTACCTATGTTTAATTGAGTTGTCATATGTTAATAACGGTATTACCGTTATTA
Read_31          GCACGTTCTACCTATGTTTAATTGAGTTGTCATATGTTAATAACGGTATTACCGTTATTA
Read_32          GCACGTTCTACCTA---------------------------------------TTAATTG
Read_33          GCACGTTCTACCTA---------------------------------------TTAATTG
Read_34          GCAC-----------------------------------------------GTTTAATTG
Read_35          GCACGTTCTACCTA---------------------------------------TTAATTG
                 **** **

WT               --------------GATT------------------------------------------
Read_1           AGTTGTCATATGTTAATAA--------T--------------------------------
Read_2           AGTTGTCATATGTTAATAACGGTATGAT--------------------------------
Read_3           AGTTGTCATATGTTAATAA--------T--------------------------------
Read_4           AGTTGTCATATGTTAATAA--------T--------------------------------
Read_5           AGTTGTCATATGTTAATAACGGTAGTTTAATTGAGTTGTCATATGTTAATAACGGTAT--
Read_6           AGTTGTCATATGTTAATAACGGTAGTTTAATTGAGTTGTCATATGTTAATAACGGTATGA
Read_7           AGTTGTCATATGTTAATAACGGTAGTTTAATTGAGTTGTCATATGTTAATAACGGTAT--
Read_8           AGTTGTCATATGTTAATAACGGTAT-----------------------------------
Read_9           AT----------------------------------------------------------
Read_10          AT----------------------------------------------------------
Read_11          AGTTGTCATATGTTAATAA-----------------------------------------
Read_12          AGTTGTCATATGTTAATAACGGTA------------------------------------
Read_13          AGTTGTCATATGTTAATAACGGTAT-----------------------------------
Read_14          AGTTGTCATATGTTAATAACGGTATGAT--------------------------------
Read_15          AGTTGTCATATGTTAATAACGGTATGAT--------------------------------
Read_16          AGTTGTCATATGTTAATAACGGTATGAT--------------------------------
Read_17          AGTTGTCATATGTTAATAACGGTAGTTTAATTGAGTTGTCATATGTTAATAACGGTATGA
Read_18          AGTTGTCATATGTTAATAACGGTAGTTTAATTGAGTTGTCATATGTTAATAACGGTATGA
Read_19          AGTTGTCATATGTTAATAACGGTATTTTAATTGAGTTGTCATATGTTAATAACGGTATGA
Read_20          AGTTGTCATATGTTAATAACGGTAGTTTAATTGAGTTGTCATATGTTAATAACGGTAT--
Read_21          AGTTGTCATATGTTAATAACGGTAGTTTAATTGAGTTGTCATATGTTAATAACGGTAT--
Read_22          AGTTGTCATATGTTAATAACGGTATGAT--------------------------------
Read_23          AGTTGTCATATGTTAATAACGGTATGA---------------------------------
Read_24          ACATATGACAACTCAATTAAAC--------------------------------------
Read_25          AGTTGTCATATGTTAATAACGGTATGAT--------------------------------
Read_26          AGTTGTCATATGTTAATAACGGTAT-----------------------------------
Read_27          ACATATGACAACTCAATTAAACAT------------------------------------
Read_28          ACATATGACAACTCAATTAAACAT------------------------------------
Read_29          ACATATGACAACTCAATTAAACAT------------------------------------
Read_30          ACATATGACAACTCAATTAAAC--------------------------------------
Read_31          ACATATGACAACTCAATTAAAC--------------------------------------
Read_32          AGTTGTCATATGTTAATAACGGTATTT---------------------------------
Read_33          AGTTGTCATATGTTAATAACGGTATTT---------------------------------
Read_34          AGTTGTCATATGTTAATAACGGTAGTTTAATTGAGTTGTCATATGTTAATAACGGTAT--
Read_35          AGTTGTCATATGTTAATAACGGTATTT---------------------------------
                                **

WT               --CCCAGCGGCGGTGCTTTCAAAGATG------------------TGATCACAGAGGGTG
Read_1           -TCCCAGCGGCGGTGCTTTCAAAGATG------------------TGATCACAGAGGGTG
Read_2           -TCCCAGCGGCGGTGCTTTCAAAGATG------------------TGATCACAGAGGGTG
Read_3           -TCCCAGCGGCGGTGCTTTCAAAGATG------------------TGATCACAGAGGGTG
Read_4           -TCCCAGCGGCGGTGCTTTCAAAGATG------------------TGATCACAGAGGGTG
Read_5           ----------------GATTCCCAGCGGCGGTGCTTTCAAAGATGTGATCACAGAGGGTG
Read_6           TTCCCAGCGGCGGTGCTTTCAAAGATG------------------TGATCACAGAGGGTG
Read_7           ----------------GATTCCCAGCGGCGGTGCTTTCAAAGATGTGATCACAGAGGGTG
```

```
Read_8          -TCCCAGCGGCGGTGCTTTCAAAGATG------------------TGATCACAGAGGGTG
Read_9          -TCCCAGCGGCGGTGCTTTCAAAGATG------------------TGATCACAGAGGGTG
Read_10         -TCCCAGCGGCGGTGCTTTCAAAGATG------------------TGATCACAGAGGGTG
Read_11         TTCCCAGCGGCGGTGCTTTCAAAGATG------------------TGATCACAGAGGGTG
Read_12         TTCCCAGCGGCGGTGCTTTCAAAGATG------------------TGATCACAGAGGGTG
Read_13         -TCCCAGCGGCGGTGCTTTCAAAGATG------------------TGATCACAGAGGGTG
Read_14         -TCCCAGCGGCGGTGCTTTCAAAGATG------------------TGATCACAGAGGGTG
Read_15         -TCCCAGCGGCGGTGCTTTCAAAGATG------------------TGATCACAGAGGGTG
Read_16         -TCCCAGCGGCGGTGCTTTCAAAGATG------------------TGATCACAGAGGGTG
Read_17         TTCCCAGCGGCGGTGCTTTCAAAGATG------------------TGATCACAGAGGGTG
Read_18         TTCCCAGCGGCGGTGCTTTCAAAGATG------------------TGATCACAGAGGGTG
Read_19         TTCCCAGCGGCGGTGCTTTCAAAGATG------------------TGATCACAGAGGGTG
Read_20         -G-------------------------------------------TGATCACAGAGGGTG
Read_21         -G-------------------------------------------TGATCACAGAGGGTG
Read_22         -TCCCAGCGGCGGTGCTTTCAAAGATG------------------TGATCACAGAGGGTG
Read_23         TTCCCAGCGGCGGTGCTTTCAAAGATG------------------TGATCACAGAGGGTG
Read_24         ----CAGCGGCGGTGCTTTCAAAGATG------------------TGATCACAGAGGGTG
Read_25         -TCCCAGCGGCGGTGCTTTCAAAGATG------------------TGATCACAGAGGGTG
Read_26         -TCCCAGCGGCGGTGCTTTCAAAGATG------------------TGATCACAGAGGGTG
Read_27         -TCCCAGCGGCGGTGCTTTCAAAGATG------------------TGATCACAGAGGGTG
Read_28         -TCCCAGCGGCGGTGCTTTCAAAGATG------------------TGATCACAGAGGGTG
Read_29         -TCCCAGCGGCGGTGCTTTCAAAGATG------------------TGATCACAGAGGGTG
Read_30         --CCCAGCGGCGGTGCTTTCAAAGATG------------------TGATCACAGAGGGTG
Read_31         --CCCAGCGGCGGTGCTTTCAAAGATG------------------TGATCACAGAGGGTG
Read_32         --CCCAGCGGCGGTGCTTTCAAAGATG------------------TGATCACAGAGGGTG
Read_33         --CCCAGCGGCGGTGCTTTCAAAGATG------------------TGATCACAGAGGGTG
Read_34         -G----------------------------TGATCACAGAGGGTG
Read_35         --CCCAGCGGCGGTGCTTTCAAAGATG------------------TGATCACAGAGGGTG
                     *    ****                               ***************
```

TALENs+ODN 3-1

```
WT              AAAGATGTTAGACACTCCTGGGCCATACTTGTTGGATGTGATTGTACCTCATCAGGAGCA
Read_1          AAAGATGTTAGACACTCCTGGGCCATACTTGTTGGATGTGATTGTACCTCATCAGGAGCA
Read_2          AAAGATGTTAGACACTCCTGGGCCATACTTGTTGGATGTGATTGTACCTCATCAGGAGCA
Read_3          AAAGATGTTAGACACTCCTGGGCCATACTTGTTGGATGTGATTGTACTTCATCAGGAGCA
Read_4          AAAGATGTTAGACACTCCTGGGCCATACTTGTTGGATGTGATTGTACCTCATCAGGAGCA
Read_5          AAAGATGTTAGACACTCCTGGGCCATACTTGTTGGATGTGATTGTACCTCATCAGGAGCA
Read_6          AAAGATGTTAGACACTCCTGGGCCATACTTGTTGGATGTGATTGTACCTCATCAGGAGCA
Read_7          AAAGATGTTAGACACTCCTGGGCCATACTTGTTGGATGTGATTGTACCTCATCAGGAGCA
Read_8          AAAGATGTTAGACACTCCTGGGCCATACTTGTTGGATGTGATTGTACCTCATCAGGAGCA
Read_9          AAAGATGTTAGACACTCCTGGGCCATACTTGTTGGATGTGATTGTACCTCATCAGGAGCA
Read_10         AAAGATGTTAGACACTCCTGGGCCATACTTGTTGGATGTGATTGTACCTCATCAGGAGCA
Read_11         AAAGATGTTAGACACTCCTGGGCCATACTTGTTGGATGTGATTGTACCTCATCAGGAGCA
Read_12         AAAGATGTTAGACACTCCTGGGCCATACTTGTTGGATGTGATTGTACCTCATCAGGAGCA
Read_13         AAAGATGTTAGACACTCCTGGGCCATACTTGTTGGATGTGATTGTACCTCATCAGGAGCA
Read_14         AAAGATGTTAGACACTCCTGGACCATACTTGTTGGATGTGATTGTACCTCATCAGGAGCA
Read_15         AAAGATGTTAGACACTCCTGGGCCATACTTGTTGGATGTGATTGTACCTCATCAGGAGCA
Read_16         AAAGATGTTAGACACTCCTGGGCCATACTTGTTGGATGTGATTGTACCTCATCAGGAGCA
Read_17         AAAGATGTTAGACACTCCTGGGCCATACTTGTTGGATGTGATTGTACCTCATCAGGAGCA
Read_18         AAAGATGTTAGACACTCCTGGGCCATACTTGTTGGATGTGATTGTACCTCATCAGGAGCA
Read_19         AAAGATGTTAGACACTCCTGGGCCATACTTGTTGGATGTGATTGTACCTCATCAGGAGCA
Read_20         AAAGATGTTAGACACTCCTGGGCCATACTTGTTGGATGTGATTGTACCTCATCAGGAGCA
Read_21         AAAGATGTTAGACACTCCTGGGCCATACTTGTTGGATGTGATTGTACCTCATCAGGAGCA
Read_22         AAAGATGTTAGACACTCCTGGGCCATACTTGTTGGATGATACCGTTATTAACATATGACA
Read_23         AAAGATGTTAGACACTCCTGGGCCATACTTGTTGGATGTGATTGTACCTCATCAGGAGCA
Read_24         AAAGATGTTCGACACTCCTGGGCCATACTTGTTGGATGTGATTGTACCCCATCCGGAGCA
Read_25         AAAGATGTTAGACACTCCTGGGCCATACTTGTTGGATGTGATTGTACCTCATCAGGAGCA
Read_26         AAAGATGTTAGACACTCCTGGGCCATACTTGTTGGATGTGATTGTACCTCATCAGGAGCA
Read_27         AAAGATGTTAGACACTCCTGGGCCATACTTGTTGGATGTGATTGTACCTCATCAGGAGCA
Read_28         AAAGATGTTAGACACTCCTGGGCCATACTTGTTGGATGTGATTGTACCTCATCAGGAGCA
Read_29         AAAGATGTTAGACACTCCTGGGCCATACTTGTTGGATGTGATTGTACCTCATCAGGAGCA
Read_30         AAAGATGTTAGACACTCCTGGGCCATACTTGTTGGATGTGATTGTACCTCATCAGGAGCA
Read_31         AAAGATGTTAGACACTCCTGGGCCATACTTGTTGGATGTGATTGTACCTCATCAGGAGCA
Read_32         AAAGATGTTAGACACTCCTGGGCCATACTTGTTGGATGTGATTGTACCTCATCAGGAGCA
Read_33         AAAGATGTTAGACACTCCTGGGCCATACTTGTTGGATGTGATTGTACCTCATCAGGAGCA
                ********* *********** ****************  *  **     *       **

WT              CGTTCTACCTTG-----------------------------------------------
Read_1          CGTTCTACCT-------------------------------------AAATTGAGTTGTC
Read_2          CGTTCTACCT--------------------------------ATGTTTAATTGAGTTGTC
```

```
Read_3              CGTTCTACCT--------------------------------ATGTTTAATTGAGTTGTC
Read_4              CGTTCTACCT-------------------------------------AAATTGAGTTGTC
Read_5              CGTTCTACCT-------------------------------------AAATTGAGTTGTC
Read_6              CGTTCTACCT-------------------------------------AAATTGAGTTGTC
Read_7              CGTTCTACCTAA------------------------------TACCGTTATTAACATATG
Read_8              CGTTCTACCTAA------------------------------TACCGTTATTAACATATG
Read_9              CGTTCTACCTAA------------------------------TACCGTTATTAACATATG
Read_10             CGTTCTACCTA-------------------------------TACCGTTATTAACATATG
Read_11             CGTTCTACCTA-------------------------------TACCGTTATTAACATATG
Read_12             CGTTCTACCT-------------------------------------AAATTGAGTTGTC
Read_13             CGTTCTACCT-------------------------------------AAATTGAGTTGTC
Read_14             CGTTCTACCTAA------------------------------TACCGTTATTAACATATG
Read_15             CGTTCTACCTAT-------------------------------ACCGTTATTAACATATG
Read_16             CGTTCTACCTGTTTAATTGAGTTGTCATATGTTAATAACGGTATGTTTAATTGAGTTGTC
Read_17             CGTTCTACCTAA--------------------------------CCGTTATTAACATATG
Read_18             CGTTCTACCTAA--------------------------------CCGTTATTAACATATG
Read_19             CGTTCTACCTA-------------------------------TACCGTTATTAACATATG
Read_20             CGTTCTACCTA-------------------------------TACCGTTATTAACATATG
Read_21             CGTTCTACCTA-------------------------------TACCGTTATTAACATATG
Read_22             -ACTCAATTAAA-------------------------------CGTTTAATTGAGTTGTC
Read_23             CGTTCTACCTAA------------------------------TACCGTTATTAACATATG
Read_24             CGTTCTACCTAA------------------------------TACCGTTATTAACATATG
Read_25             CGTTCTACCTAA------------------------------TACCGTTATTAACATATG
Read_26             CGTTCTACCTAA------------------------------TACCGTTATTAACATATG
Read_27             CGTTCTACCTAT------------------------------GAGTTTAATTGAGTTGTC
Read_28             CGTTT-------------------------------------------AATTGAGTTGTC
Read_29             CGTTCTACCTA-------------------------------TACCGTTATTAACATATG
Read_30             CGTTCTACCTA-------------------------------TACCGTTATTAACATATG
Read_31             CGTTCTACCT-------------------------------------AAATTGAGTTGTC
Read_32             CGTTCTACCTA-------------------------------TACCGTTATTAACATATG
Read_33             CGTTCTACCTAA--------------------------------CCGTTATTAACATATG
                       *                                                      *

WT                  A-----------------------------------------------------------
Read_1              ATATGTTAATAACGGTAT------------------------------------------
Read_2              ATATGTTAATAACGGTAT------------------------------------------
Read_3              ATATGTTAATAACGGTAT------------------------------------------
Read_4              ATATGTTAATAACGGTAT------------------------------------------
Read_5              ATATGTTAATAACGGTAT------------------------------------------
Read_6              ATATGTTAATAACGGTAT------------------------------------------
Read_7              ACAACTCAATTAAACATACCGTTATTAACATATGACAACTCAATTAAACGTTTAATTGAG
Read_8              ACAACTCAATTAAACG--------------------------------------------
Read_9              ACAACTCAATTAAACG--------------------------------------------
Read_10             ACAACTCAATTAAACG--------------------------------------------
Read_11             ACAACTCAATTAAACG--------------------------------------------
Read_12             ATATGTTAATAACGGTAT------------------------------------------
Read_13             ATATGTTAATAACGGTAT------------------------------------------
Read_14             ACAACTCAATTAAACG--------------------------------------------
Read_15             ACAACTCAATTAAACA--------------------------------------------
Read_16             ATATGTTAATAACGGTAT------------------------------------------
Read_17             ACAACTCAATTAAAC-----------------TGA-------------------------
Read_18             ACAACTCAATTAAAC-----------------TGA-------------------------
Read_19             ACAACTCAATTAAACG--------------------------------------------
Read_20             ACAACTCAATTAAACG--------------------------------------------
Read_21             ACAACTCAATTAAACG--------------------------------------------
Read_22             ATATGTTAATAACGGTATATACCGTTATTAACATATGACAACTC----------------
Read_23             ACAACTCAATTAAACG--------------------------------------------
Read_24             ACAACTCAATTACACG--------------------------------------------
Read_25             ACAACTCAATTAAACATACCGTTATTAACATATGACAACTCAATTAAACGTTTAATTGAG
Read_26             ACAACTCAATTAAACATACCGTTATTAACATATGACAACTCAATTAAACGTTTAATTGAG
Read_27             ATATGTTAATAACGGTATTACCGTTATTAACATATGACAACTCAATTAAACGTTTAATTG
Read_28             ATATGTTAATAACGGTATAA-----ACCCATGGCCCACCAGTGCCTCGTGGTGCTGCGCC
Read_29             ACAACTCAATTAAACTTTAATTGAGTTGTCATATGTTAATAACGGTATG-----------
Read_30             ACAACTCAATTAAACTTTAATTGAGTTGTCATATGTTAATAACGGTATG-----------
Read_31             ATATGTTAATAACGGTAT------------------------------------------
Read_32             ACAACTCAATTAAACTTTAATTGAGTTGTCATATGTTAATAACGGTATG-----------
Read_33             ACAACTCAATTAAAC-----------------TGA-------------------------
                    *

WT                  -------------------------TTCCCAGCGGCGGTGCTTTCAAAGATGTGATCACA
Read_1              -----------------------GATTCCCAGCGGCGGTGCTTTCAAAGATGTGATTACA
```

```
Read_2          -----------------------GATTCCCAGCGGCGGTGCTTTCAAAGATGTGATCACA
Read_3          -----------------------GATTCCCAGCGGCGGTGCTTTCAAAGATGTGATCACA
Read_4          -----------------------GATTCCCAGCGGCGGTGCTTTCAAAGATGTGATCACA
Read_5          -----------------------GATTCCCAGCGGCGGTGCTTTCAAAGATGTGATCACA
Read_6          -----------------------GATTCCCAGCGGCGGTGCTTTCAAAGATGTGATCACA
Read_7          TTGTCATATGTTAATAACGGTA-TATTCCCAGCGGCGGTGCTTTCAAAGATGTGATCACA
Read_8          ------------------------ATTCCCAGCGGCGGTGCTTTCAAAGATGTGATCACA
Read_9          ------------------------ATTCCCAGCGGCGGTGCTTTCAAAGATGTGATCACA
Read_10         ------------------------ATTCCCAGCGGCGGTGCTTTCAAAGATGTGATCACA
Read_11         ------------------------ATTCCCAGCGGCGGTGCTTTCAAAGATGTGATCACA
Read_12         -----------------------GATTCCCAGCGGCGGTGCTTTCAAAGATGTGATCACA
Read_13         -----------------------GATTCCCAGCGGCGGTGCTTTCAAAGATGTGATCACA
Read_14         ------------------------ATTCCCAGCGGCGGTGCTTTCAAAGATGTGATCACA
Read_15         -------------------------TTCCCAGCGGCGGTGCTTTCAAAGATGTGATCACA
Read_16         -------------------------TTCCCAGCGGCGGTGCTTTCAAAGATGTGATCACA
Read_17         -------------------------TTCCCAGCGGCGGTGCTTTCAAAGATGTGATCACA
Read_18         -------------------------TTCCCAGCGGCGGTGCTTTCAAAGATGGGATCACA
Read_19         ------------------------ATTCCCAGCGGCGGTGCTTTCAAAGATGTGATCACA
Read_20         ------------------------ATTCCCAGCGGCGGTGCTTTCAAAGATGTGATCACA
Read_21         ------------------------ATTCCCAGCGGCGGTGCTTTCAAAGATGTGATCACA
Read_22         -----------------------AATTAAACGCGGCGGTGCTTTCAAAGATGTGATCACA
Read_23         ------------------------ATTCCCAGCGGCGGTGCTTTCAAAGATGTGATCACA
Read_24         ------------------------ATTCCCAGCGGAGGTGCTTTCAAAGATGTGATCACA
Read_25         TTGTCATATGTTAATAACGGTA-TATTCCCAGCGGCGGTGCTTTCAAAGATGTGATCACA
Read_26         TTGTCATATGTTAATAACGGTA-TATTCCCAGCGGCGGTGCTTTCAAAGATGTGATCACA
Read_27         AGTTGTCATATGTTAATAACGGTATTTCCCAGCGGCGGTGCTTTCAAAGATGTGATCACA
Read_28         ACTGTCGAACGCACCTTCGGTTTGATTCCCAGCGGCGGTGCTTTCAAAGATGTGATCACA
Read_29         ------------------------ATTCCCAGCGGCGGTGCTTTCAAAGATGTGATCACA
Read_30         ------------------------ATTCCCAGCGGCGGTGCTTTCAAAGATGTGATCACA
Read_31         -----------------------GATTCCCAGCGGCGGTGCTTTCAAAGATGTGATCACA
Read_32         ------------------------ATTCCCAGCGGCGGTGCTTTCAAAGATGTGATCACA
Read_33         -------------------------TTCCCAGCGGCGGTGCTTTCAAAGATGTGATCACA
                                         **    **** **************** *** ***
```

TALENs+ODN 3-2

```
WT              CGTTCTACCTAT---GATTCC---------------------------------------
Read_1          CGTTCTACCT----GTTTAATTGAGTTGTCATATGTTAATAACGGTATATACCGTTATTA
Read_2          CGTTCTACCTAT--GAATACCGTTATTAACATATGACAACTCAATTAAAC----------
Read_3          CGTTCTACCTAT--GAATACCGTTATTAACATATGACAACTCAATTAAAC----------
Read_4          CGTTCTACCTAT--GAATACCGTTATTAACATATGACAACTCAATTAAAC----------
Read_5          CGTTCTACCTAT--GAATACCGTTATTAACATATGACAACTCAATTAAAC----------
Read_6          CGTTCTACCT----GTTTAATTGAGTTGTCATATGTTAATAACGGTAT------------
Read_7          CGTTCTACCT----GTTTAATTGAGTTGTCATATGTTAATAACGGTAT------------
Read_8          CGTTCTACCTA-----ATACCGTTATTAACATATGACAACTCAATTAAACAT--------
Read_9          CGTTCTACCTAT---GATACCGTTATTAACATATGACAACTCAATTAAACAT--------
Read_10         CGTTCTACCTAT---GATACCGTTATTAACATATGACAACTCAATTAAACAT--------
Read_11         CGTTCTACCTATGTGTTTAATTGAGTTGTCATATGTTAATAACGGTAT------------
Read_12         CGTTCTACCTAT---GATACCGTTATTAACATATGACAACTCAATTAAACAT--------
Read_13         CGTTCTACCTA-----ATACCGTTATTAACATATGACAACTCAATTAAACAT--------
Read_14         CGTTCTACCTA-----ATACCGTTATTAACATATGACAACTCAATTAAACAT--------
Read_15         CGTTCTACCT----GTTTAATTGAGTTGTCATATGTTAATAACGGTAT------------
Read_16         CGTTCTACCT----GTTTAATTGAGTTGTCATATGTTAATAACGGTAT------------
Read_17         CGTTCTACCT----GTTTAATTGAGTTGTCATATGTTAATAACGGTAT------------
Read_18         CGTTCTACCT----GTTTAATTGAGTTGTCATATGTTAATAACGGTAT------------
Read_19         CGTTCTACCT----GTTTAATTGAGTTGTCATATGTTAATAACGGTAT------------
Read_20         CGTTCTACCTAT---GATACCCTTATTAACATATGACAACTCAATTAAACAT--------
                **********       *

WT              ------------------------------------------C-----------------
Read_1          ACATATGACAACTCAATTAAACGTTTAATTGAGTTGTCATATGTTAATAACGGTAGATTC
Read_2          ----------------------GTTTAATTGAGTTGTCATATGTTAATAACGGTAT----
Read_3          ----------------------GTTTAATTGAGTTGTCATATGTTAATAACGGTAT----
Read_4          ----------------------GTTTAATTGAGTTGTCATATGTTAATAACGGTAT----
Read_5          ----------------------GTTTAATTGAGTTGTCATATGTTAATAACGGTAT----
Read_6          ----------------------GTTTAATTGAGTTGTCATATGTTAATAACGGTAT----
Read_7          ----------------------GTTTAATTGAGTTGTCATATGTTAATAACGGTAT----
Read_8          ---------------------ACCGTTATTAACATATGACAACTCAATTAAAC-------
Read_9          ---------------------ACCGTTATTAACATATGACAACTCAATTAAACATGATTC
Read_10         ---------------------ACCGTTATTAACATATGACAACTCAATTAAACATGATTC
Read_11         ---------------------------AT--GATTCCCA---------------------
```

```
Read_12         ---------------------ACCGTTATTAACATATGACAACTCAATTAAACATGATTC
Read_13         ---------------------ACCGTTATTAACATATGACAACTCAATTAAAC-------
Read_14         ---------------------ACCGTTATTAACATATGACAACTCAATTAAAC-------
Read_15         ---------------------GTTTAATTGAGTTGTCATATGTTAATAACGGTAT-----
Read_16         ---------------------GTTTAATTGAGTTGTCATATGTTAATAACGGTAT-----
Read_17         ---------------------GTTTAATTGAGTTGTCATATGTTAATAACGGTAT-----
Read_18         ---------------------GTTTAATTGAGTTGTCATATGTTAATAACGGTAT-----
Read_19         ---------------------GTTTAATTGAGTTGTCATATGTTAATAACGGTAT-----
Read_20         ---------------------ACCGTTATTAACATATGGCACCTCAATTAAACATGATTC

WT              --AGCGGCGGTGCTTTCAAAGATGTGATCACAGAGGGTGATGGGAGACGTTCCTATTGAC
Read_1          CCAGCGGCGGTGCTTTCAAAGATGTGATCACAGAGGGTGATGGGAGACGTTCCTATTGAC
Read_2          ---GCGGCGGTGCTTTCAAAGATGTGATCACAGAGGGTGATGGGAGACGTTCCTATTGAC
Read_3          ---GCGGCGGTGCTTTCAAAGATGTGATCACAGAGGGTGATGGGAGACGTTCCTATTGAC
Read_4          ---GCGGCGGTGCTTTCAAAGATGTGATCACAGAGGGTGATGGGAGACGTTCCTATTGAC
Read_5          ---GCGGCGGTGCTTTCAAAGATGTGATCACAGAGGGTGATGGGAGACGTTCCTATTGAC
Read_6          ---GCGGCGGTGCTTTCAAAGATGTGATCACAGAGGGTGATGGGAGACGTTCCTATTGAC
Read_7          ---GCGGCGGTGCTTTCAAAGATGTGATCACAGAGGGTGATGGGAGACGTTCCTATTGAC
Read_8          -CAGCGGCGGTGCTTTCAAAGATGTGATCACAGAGGGTGATGGGAGACGTTCCTATTGAC
Read_9          CCAGCGGCGGTGCTTTCAAAGATGTGATCACAGAGGGTGATGGGAGACGTTCCTATTGAC
Read_10         CCAGCGGCGGTGCTTTCAAAGATGTGATCACAGAGGGTGATGGGAGACGTTCCTATTGAC
Read_11         ---GCGGCGGTGCTTTCAAAGATGTGATCACAGAGGGTGATGGGAGACGTTCCTATTGAC
Read_12         CCAGCGGCGGTGCTTTCAAAGATGTGATCACAGAGGGTGATGGGAGACGTTCCTATTGAC
Read_13         -CAGCGGCGGTGCTTTCAAAGATGTGATCACAGAGGGTGATGGGAGACGTTCCTATTGAC
Read_14         -CAGCGGCGGTGCTTTCAAAGATGTGATCACAGAGGGTGATGGGAGACGTTCCTATTGAC
Read_15         ---GCGGCGGTGCTTTCAAAGATGTGATCACAGAGGGTGATGGGAGACGTTCCTATTGAC
Read_16         ---GCGGCGGTGCTTTCAAAGATGTGATCACAGAGGGTGATGGGAGACGTTCCTATTGAC
Read_17         ---GCGGCGGTGCTTTCAAAGATGTGATCACAGAGGGTGATGGGAGACGTTCCTATTGAC
Read_18         ---GCGGCGGTGCTTTCAAAGATGTGATCACAGAGGGTGATGGGAGACGTTCCTATTGAC
Read_19         ---GCGGCGGTGCTTTCAAAGATGTGATCACAGAGGGTGATGGGAGACGTTCCTATTGAC
Read_20         CCAGCGGCGGTGCTTTCAAAGATGTGATCACAGAGGGTGATGGGAGACGTTCCTATTGAC
                   *********************************************************
```

TALENs+ODN 3-3

```
WT              TCAAAAGATGTTAGACACTCCTGGGCCATACTTGTTGGATGTGATTGTACCTCATCAGGA
Read_1          TCAAAAGATGTTAGACACTCCTGGGCCATACTTGTTGGATGTGATTGTACCTCATCAGGA
Read_2          TCAAAAGATGTTAGACACTCCTGGGCCATACTTGTTGGATGTGATTGTACCTCATCAGGA
Read_3          TCAAAAGATGTTAGACACTCCTGGGCCATACTTGTTGGATGTGATTGTACCTCATCAGGA
Read_4          TCAAAAGATGTTAGACACTCCTGGGCCATACTTGTTGGATGTGATTGTACCTCATCAGGA
Read_5          TCAAAAGATGTTAGACACTCCTGGGCCATACTTGTTGGATGTGATTGTACCTCATCAGGA
Read_6          TCAAAAGATGTTAGACACTCCTGGGCCATACTTGTTGGATGTGATTGTACCTCATCAGGA
Read_7          TCAAAAGATGTTAGACACTCCTGGGCCATACTTGTTGGATGTGATTGTACCTCATCAGGA
Read_8          TCAAAAGATGTTAGACACTCCTGGGCCATACTTGTTGGATGTGATTGTACCTCATCAGGA
Read_9          TCAAAAGATGTTAGACACTCCTGGGCCATACTTGTTGGATGTGATTGTACCTCATCAGGA
Read_10         TCAAAAGATGTTAGACACTCCTGGGCCATACTTGTTGGATGTGATTGTACCTCATCAGGA
Read_11         TCAAAAGATGTTAGACACTCCTGGGCCATACTTGTTGGATGTGATTGTACCTCATCAGGA
Read_12         TCAAAAGATGTTAGACACTCCTGGGCCATACTTGTTGGATGTGATTGTACCTCATCAGGA
Read_13         TCAAAAGATGTTAGACACTCCTGGGCCATACTTGTTGGATGTGATTGTACCTCATCAGGA
Read_14         TCAAAAGATGTTAGACACTCCTGGGCCATACTTGTTGGATGTGATTGAG--TTGTCA---
Read_15         TCAAAAGATGTTAGACACTCCTGGGCCATACTTGTTGGATGTGATTGTACCTCATCAGGA
Read_16         TCAAAAGATGTTAGACACTCCTGGGCCATACTTGTTGGATGTGATTGTACCTCATCAGGA
Read_17         TCAAAAGATGTTAGACACTCCTGGGCCATACTTGTTGGATGTGATTGTACCTCATCAGGA
Read_18         TCAAAAGATGTTAGACACTCCTGGGCCATACTTGTTGGATGTGATTGTACCTCATCAGGA
Read_19         TCAAAAGATGTTAGACACTCCTGGGCCATACTTGTTGGATGTGATTGTACCTCATCAGGA
Read_20         TCAAAAGATGTTAGACACTCCTGGGCCATACTTGTTGGATGTGATTGTACCTCATCAGGA
Read_21         TCAAAAGATGTTAGACACTCCTGGGCCATACTTGTTGGATGTGATTGTACCTCATCAGGA
Read_22         TCAAAAGATGTTAGACACTCCTGGGCCATACTTGTTGGATGTGATTGTACCTCATCAGGA
Read_23         TCAAAAGATGTTAGACACTCCTGGGCCATACTTGTTGGATGTGATTGTACCTCATCAGGA
Read_24         TCAAAAGATGTTAGACACTCCTGGGCCATACTTGTTGGATGTGATTGAG--TTGTCA---
Read_25         TCAAAAGATGTTAGACACTCCTGGGCCATACTTGTTGGATGTGATTGAG--TTGTCA---
Read_26         TCAAAAGATGTTAGACACTCCTGGGCCATACTTGTTGGATGTGATTGTACCTCATCAGGA
Read_27         TCAAAAGATGTTAGACACTCCTGGGCCATACTTGTTGGATGTGATTGTACCTCATCAGGA
Read_28         TCAAAAGATGTTAGACACTCCTGGGCCATACTTGTTGGATGTGATTGTACCTCATCAGGA
Read_29         TCAAAAGATGTTAGACACTCCTGGGCCATACTTGTTGGATGTGATTGTACCTCATCAGGA
Read_30         TCAAAAGATGTTAGACACTCCTGGGCCATACTTGTTGGATGTGATTGAG--TTGTCA---
Read_31         TCAAAAGATGTTAGACACTCCTGGGCCATACTTGTTGGATGTGATTGTACCTCATCAGGA
Read_32         TCAAAAGATGTTAGACACTCCTGGGCCATACTTGTTGGATGTGATTGTACCTCATCAGGA
Read_33         TCAAAAGATGTTAGACACTCCTGGGCCATACTTGTTGGATGTGATTGTACCTCATCAGGA
Read_34         TCAAAAGATGTTAGACACTCCTGGGCCATACTTGTTGGATGTGATTGTACCTCATCAGGA
```

```
Read_35         TCAAAAGATGTTAGACACTCCTGGGCCATACTTGTTGGATGTGATTGTACCTCATCAGGA
Read_36         TCAAAAGATGTTAGACACTCCTGGGCCATACTTGTTGGATGTGATTGTACCTCATCAGGA
Read_37         TCAAAAGATGTTAGACACTCTTGGGCCATACTTGTTGGATGTGATTGTACCTCATCAGGA
Read_38         TCAAAAGATGTTAGACACTCTTGGGCCATACTTGTTGGATGTGATTGTACCTCATCAGGA
Read_39         TCAAAAGATGTTAGACACTCCTGGGCCATACTTGTTGGATGTGATTGTACCTCATCAGGA
Read_40         TCAAAAGATGTTAGACACTCCTGGGCCATACTTGTTGGATGTGATTGTACCTCATCAGGA
                ******************** **************************    *  ***

WT              GCACGTTCTACCTATGAT------------------------------------------
Read_1          GCACGTTCTACCT---ATACCGTTATTAACATATGACAACTCAATTAAAC----------
Read_2          GCACGTTCTACCTAT-----------------------------------GTTTAATTGA
Read_3          GCACGTTCTACCTA--ATACCGTTATTAACATATGACAACTCAATTAAA-GTTTAATTGA
Read_4          GCACGTTCTACCTA--ATACCGTTATTAACATATGACAACTCAATTAAA-GTTTAATTGA
Read_5          GCACGTTCTACCTA--ATACCGTTATTAACATATGACAACTCAATTAAA-GTTTAATTGA
Read_6          GCACGTTCTACCT---ATACCGTTATTAACATATGACAACTCAATTAAAC----------
Read_7          GCACGTTCTACCT---ATACCGTTATTAACATATGACAACTCAATTAAAC----------
Read_8          GCACGTTCTACCTATGATACCCTTATTAACATATGACAACTCAATTAAACGTTTAATTGA
Read_9          GCACGTTCTACCTATGATACCGTTATTAACATATGACAACTCAATTAAACGTTTAATTGA
Read_10         GCACGTTCTACCTA--ATACCGTTATTAACATATGACAACTCAATTAAA-GTTTAATTGA
Read_11         GCACGTTTTACCTA--ATACCGTTATTAACATATGACAACTCAATTAAA-GTTTAATTGA
Read_12         GCACGTTCTACCTA--ATACCGTTATTAACATATGACAACTCAATTAAA-GTTTAATTGA
Read_13         GCACGT----------GTTTAATTGAGTTGTCATATGTTAATAACGGTATGATTC-----
Read_14         -TATGTT-----------------------------------------------------
Read_15         GCACGTTCTACCTATGGTTTAATTGAGTTGTCATATGTTAATAAGTTTAATTCAGTT
Read_16         GCACGTTCTACCT---ATACCGTTATTAACATATGACAACTCAATTAAAC----------
Read_17         GCACGTTCTACCT---ATACCGTTATTAACATATGACAACTCAATTAAAC----------
Read_18         GCACGTTCTACCT---ATACCGTTATTAACATATGACAACTCAATTAAAC----------
Read_19         GCACGTTCTACCT---ATACCGTTATTAACATATGACAACTCAATTAAAC----------
Read_20         GCACGTTCTACCT---GTTTAATTGAGTTGTCATATGTTAATAACGGTACCGTTATTAA-
Read_21         GCACGTTCTACCT---GTTTAATTGAGTTGTCATATGTTAATAACGGTACCGTTATTAA-
Read_22         GCACGTTCTACCT---GTTTAATTGAGTTGTCATATGTTAATAACGGTAT-GTTTAATTG
Read_23         GCACGTTCTACCT---GTTTAATTGAGTTGTCATATGTTAATAACGGTAT-GTTTAATTG
Read_24         -TATGTT-----------------------------------------------------
Read_25         -TATGTT-----------------------------------------------------
Read_26         GCACGTTCTACCTATGATACCGTTATTAACATATGACAACTCAATTAAACGTTTAATTGA
Read_27         GCACGTTCTACCT---ATACCGTTATTAACATATGACAACTCAATTAAAC----------
Read_28         GCACGTTCTACCT---ATACCGTTATTAACATATGACAACTCAATTAAAC----------
Read_29         GCACGTTCTACCT---ATACCGTTATTAACATATGACAACTCAATTAAACGTTTAATTGA
Read_30         -TATGTT-----------------------------------------------------
Read_31         GCACGTTCTACCT---GTTTAATTGAGTTGTCATATGTTAATAACGGTATGTTTAATTGA
Read_32         GCACGTTCTACCT---ATACCGTTATTAACATATGACAACTCAATTAAAC----------
Read_33         GCACGTTCTACCT---ATACCGTTATTAACATATGACAACTCAATTAAAC----------
Read_34         GCACGTTCTACCT---GTTTAATTGAGTTGTCATATGTTAATAACGGTATGTTTAATTGA
Read_35         GCACGTTCTACCT---GTTTAATTGAGTTGTCATATGTTAATAACGGTATGTTTAATTGA
Read_36         GCACGTTCTACCT---ATACCGTTATTAACATATGACAACTCAATTAAACGTTTAATTGA
Read_37         GCACGTTCTACCT---ATACCGTTATTAACATATGACAACTCAATTAAACGTTTAATTGA
Read_38         GCCCGTTCTACCT---ATACCGTTATTAACATATGACAACTCAATTAAACGTTTAATTGA
Read_39         GCACGTTCTACCTA--ATACCGTTATTAACATATGACAACTCAATTAAA-GTTTAATTGA
Read_40         GCACGTTCTACCTA--ATACCGTTATTAACATATGACAACTCAATTAAA-GTTTAATTGA
                    **

WT              ------------------------------------------------------------
Read_1          -------------------------T----------------------------------
Read_2          GTTGTCATATGTTAATAACGGTAT-----------------------------------A
Read_3          GTTGTCATATGTTAATAACGGTAT--------------------------------ATGA
Read_4          GTTGTCATATGTTAATAACGGTAT--------------------------------ATGA
Read_5          GTTGTCATATGTTAATAACGGTAT--------------------------------ATGA
Read_6          -------------------------T----------------------------------
Read_7          ------------------------------------------------------------
Read_8          TTTGTCATATGTTAATAACGGTATATACCGTTATTAACAAATGACAACTCAATTAAACGA
Read_9          GTTGTCATATGTTAATAACGGTATATACCGTTATTAACATATGACAACTCAATTAAACGA
Read_10         GTTGTCATATGTTAATAACGGTAT--------------------------------GGGA
Read_11         GTTGTCATATGTTTATAACGGTAT--------------------------------GTGA
Read_12         GTTGTCATATGTTAATAACGGTAT--------------------------------GTGA
Read_13         ------------------------------------------------------------
Read_14         ------------------------------------------------------------
Read_15         GTCATATGTTAATAACGGTATATACCGTTATTAACATATGACAACTTAATTAAAC-ATGA
Read_16         ----------------------TACCGTTATTAACATATGACAACTCAATTAAAC-----
Read_17         ----------------------TACCGTTATTAACATATGACAACTCAATTAAAC-----
Read_18         ------------------------------------------------------------
Read_19         ------------------------------------------------------------
```

```
Read_20        CATATGACAACTCAATTAAAC-TACCGTTATTAACATATGACAACTCAATTAAAC-----
Read_21        CATATGACAACTCAATTAAAC-TACCGTTATTAACATATGACAACTCAATTAAAC-----
Read_22        AGTTGTCATATGTTAATAACGGTATG----------------------------------A
Read_23        AGTTGTCATATGTTAATAACGGTAT----------------------------------GA
Read_24        ------------------------------------------------------------
Read_25        ------------------------------------------------------------
Read_26        GTTGTCATATGTTAATAACGGTATATACCGTTATTAACATATGACAACTCAATTAAACGA
Read_27        -------------------------T----------------------------------
Read_28        -------------------------T----------------------------------
Read_29        GTTGTCATATGTTAATAACGGTATATACCGTTATTAACATATGACAACTCAATTAAACGA
Read_30        ------------------------------------------------------------
Read_31        GTTGTCATATGTTAATAACGGTAT----------------------------------GA
Read_32        -------------------------TACCGTTATTAACATATGACAACTCAATTAAAC--
Read_33        -------------------------TACCGTTATTAACATATGACAACTCAATTGAAC--
Read_34        GTTGTCATATGTTAATAACGGTAT----------------------------------GA
Read_35        GTTGTCATATGTTAATAACGGTAT----------------------------------GA
Read_36        GTTGTCATATGTTAATAACGGTATATACCGTTATTAACATATGACAACTCAATTAAACGA
Read_37        GTTGTCATATGTTAATAACGGTATATACCGTTATTAACATATGACAACTCAATTAAACGA
Read_38        GTTGTCATATGTTAATAACGGTATATACCGTTATTAACATATGACAACTCAATTAAACGA
Read_39        GTTGTCATATGTTAATAACGGTATAT--------------------------------GA
Read_40        GTTGTCATATGTTAATAACGGTATAT--------------------------------GA


WT             -TCCCAGCGGCGGTGCTTTCAAAGATGTGATCACAGAGGGTGATGGGAGACGTTCCTATT
Read_1         -TCCCAGCGGCGGTGCTTTCAAAGATGTGATCACAGAGGGTGATGGGAGACGTTCCTATT
Read_2         TTCCCAGCGGCGGTGCTTTCAAAGATGTGATCACAGAGGGTGATGGGAGACGTTCCTATT
Read_3         TTCCCAGCGGCGGTGCTTTCAAAGATGTGATCACAGAGGGTGATGGGAGACGTTCCTATT
Read_4         TTCCCAGCGGCGGTGCTTTCAAAGATGTGATCACAGAGGGTGATGGGAGACGTTCCTATT
Read_5         TTCCCAGCGGCGGTGCTTTCAAAGATGTGATCACAGAGGGTGATGGGAGACGTTCCTATT
Read_6         TTCCCAGCGGCGGTGCTTTCAAAGATGTGATCACAGAGGGTGATGGGAGACGTTCCTATT
Read_7         TTCCCAGCGGCGGTGCTTTCAAAGATGTGATCACAGAGGGTGATGGGAGACGTTCCTATT
Read_8         TTCCCAGCGGCGGTGCTTTCAAAGATGTGATCACAGAGGGTGATGGGAGACGTTCCTATT
Read_9         TTCCCAGCGGCGGTGCTTTCAAAGATGTGATCACAGAGGGTGATGGGAGACGTTCCTATT
Read_10        TTCCCAGCGGCGGTGCTTTCAAAGATGTGATCACAGAGGGTGATGGGAGACGTTCCTATT
Read_11        TTCCCAGCGGCGGTGCTTTCAAAGATGTGATCACAGAGGGTGATGGGAGACGTTCCTATT
Read_12        TTCCCAGCGGCGGTGCTTTCAAAGATGTGATCACAGAGGGTGATGGGAGACGTTCCTATT
Read_13        ---CCAGCGGCGGTGCTTTCAAAGATGTGATCACAGAGGGTGATGGGAGACGTTCCTATT
Read_14        -----AATAACGGTA--TTCAAAGATGTGATCACAGAGGGTGATGGGAGACGTTCCTATT
Read_15        TTCCCAGCGGCGGTGCTTTCAAAGATGTGATCACAGAGGGTGATGGGAGACGTTCCTATT
Read_16        TTCCCAGCGGCGGTGCTTTCAAAGATGTGATCACAGAGGGTGATGGGAGACGTTCCTATT
Read_17        TTCCCAGCGGCGGTGCTTTCAAAGATGTGATCACAGAGGGTGATGGGAGACGTTTCTATT
Read_18        TTCCCAGCGGCGGTGCTTTCAAAGATGTGATCACAGAGGGTGATGGGAGACGTTCCTATT
Read_19        -TCCCAGCGGCGGTGCTTTCAAAGATGTGATCACAGAGGGTGATGGGAGACGTTCCTATT
Read_20        ----CAGCGGCGGTGCTTTCAAAGATGTGATCACAGAGGGTGATGGGAGACGTTCCTATT
Read_21        ----CAGCGGCGGTGCTTTCAAAGATGTGATCACAGAGGGTGATGGGAGACGTTCCTATT
Read_22        TTCCCAGCGGCGGTGCTTTCAAAGATGTGATCACAGAGGGTGATGGGAGACGTTCCTATT
Read_23        TTCCCAGCGGCGGTGCTTTCAAAGATGTGATCACAGAGGGTGATGGGAGACGTTCCTATT
Read_24        -----AATAACGGTA--TTCAAAGATGTGATCACAGAGGGTGATGGGAGACGTTCCTATT
Read_25        -----AATAACGGTA--TTCAAAGATGTGATCACAGAGGGTGATGGGAGACGTTCCTATT
Read_26        TTCCCAGCGGCGGTGCTTTCAAAGATGTGATCACAGAGGGTGATGGGAGACGTTCCTATT
Read_27        -TCCCAGCGGCGGTGCTTTCAAAGATGTGATCACAGAGGGTGATGGGAGACGTTCCTATT
Read_28        -TCCCAGCGGCGGTGCTTTCAAAGATGTGATCACAGAGGGTGATGGGAGACGTTCCTATT
Read_29        TTCCCAGCGGCGGTGCTTTCAAAGATGTGATCACAGAGGGTGATGGGAGACGTTCCTATT
Read_30        -----AATAACGGTA--TTCAAAGATGTGATCACAGAGGGTGATGGGAGACGTTCCTATT
Read_31        TTCCCAGCGGCGGTGCTTTCAAAGATGTGATCACAGAGGGTGATGGGAGACGTTCCTATT
Read_32        TTCCCAGCGGCGGTGCTTTCAAAGATGTGATCACAGAGGGTGATGGGAGACGTTCCTATT
Read_33        TTCCCAGCGGCGGTGCTTTCAAAGATGTGATCACAGAGGGTGATGGGAGACGTTCCTATT
Read_34        TTCCCAGCGGCGGTGCTTTCAAAGATGTGATCACAGAGGGTGATGGGAGACGTTCCTATT
Read_35        TTCCCAGCGGCGGTGCTTTCAAAGATGTGATCACAGAGGGTGATGGGAGACGTTCCTATT
Read_36        TTCCCAGCGGCGGTGCTTTCAAAGATGTGATCACAGAGGGTGATGGGAGACGTTCCTATT
Read_37        TTCCCAGCGGCGGTGCTTTCAAAGATGTGATCACAGAGGGTGATGGGAGACGTTCCTATT
Read_38        TTCCCAGCGGCGGTGCTTTCAAAGATGTGATCACAGAGGGTGATGGGAGACGTTCCTATT
Read_39        TTCCCAGCGGCGGTGCTTTCAAAGATGTGATCACAGAGGGTGATGGGAGACGTTCCTATT
Read_40        TTCCCAGCGGCGGTGCTTTCAAAGATGTGATCACAGAGGGTGATGGGAGACGTTCCTATT
                    *    ****   ************************************* *****
```

CRISPR 1-2

```
WT              CGTTCTACCTATGATTC---------------------------------CCAGCGGCGG
Read_1          CGTTCTACCTATGATTCGTTTAATTGAGTTGTCATATGTTAATAACGGTATCAGCGGCGG
Read_2          CGTTCTACCTATGATTCGTTTAATTGAGTTGTCATATGTTAATAACGGTATCAGCGGCGG
Read_3          CGTTCTACCTATGATTCGTTTAATTGAGTTGTCATATGTTAATAACGGTATCAGCGGCGG
Read_4          CGTTCTACCTATGATTCGTTTAATTGAGTTGTCATATGTTAATAACGGTATCAGCGGCGG
                *****************                                  *********
```

CRISPR 2-2

```
WT              CGTTCTACCTATGATTCC-----------------------------------CAGCGGC
Read_1          CGTTCTACCTATGATTCCATACCGTTATTAACATATGACAACTCAATTAAACACAGCGGC
Read_2          CGTTCTACCTATGATTCCATACCGTTATTAACATATGACAACTCAATTAAACACAGCGGC
Read_3          CGTTCTACCTATGATTCCATACCGTTATTAACATATGACAACTCAATTAAACACAGCGGC
Read_4          CGTTCTACCTATGATTCCATACCGTTATTAACATATGACAACTCAATTAAACACAGCGGC
Read_5          CGTTCTACCTATGATTCCATACCGTTATTAACATATGACAACTCAATTAAACACAGCGGC
Read_6          AGTTCTACCT--------ATACCGTTATTAACATATGACAACTCAATTAAAC-CAGCGGC
Read_7          CGTTCTACCT--------ATACCGTTATTAACATATGACAACTCAATTAAAC-CAGCGGC
Read_8          CGTTCTACCT--------ATACCGTTATTAACATATGACAACTCAATTAAAC-CAGCGGC
                 ***********                                         *******
```

CRISPR 3-1

```
WT              ----CATACTTGTTGGATGTGATTGTACCTCATCAGGAGCACGTTCTACCTATGATTCC-
Read_1          ----CATACTTGTTGGATGTGATTGTACCTCATCAGGAGCACGTTCTACCTATGATTCCA
Read_2          ----CATACTTGTTGGATGTGATTGTACCTCATCAGGAGCACGTTCTACCTATGATTCCA
Read_3          ACTACATAATTGTTGGATGTGATTGTACCTCATCAGGAGCACGTTCTACCTATGATTCCA
                    **** **************************************************

WT              ---------------------------------CAGCGGCGGTGCTTTCAAAGATGTGAT
Read_1          TACCGTTATTAACATATGACAACTCAATTAAACCAGCGGCGGTGCTTTCAAAGATGTGAT
Read_2          TACCGTTATTAACATATGACAACTCAATTAAACCAGCGGCGGTGCTTTCAAAGATGTGAT
Read_3          TACCGTTATTAACATATGACAACTCAATTAAACCAGCGGCGGTGCTTTCAAAGATGTGAT
                                                 ***************************
```

CRISPR 3-2

```
WT              CGTTCTACCTATGATTCC---------------------------------CAGCGGCGG
Read_1          CGTTCTACCTATGATTCCTACCGTTATTAACATATGACAACTCAATTAAACCAGCGGCGG
                ******************                                 *********
```

CRISPR 3-3

```
WT              CGTTCTACCTATGATTCC------------------------------------------
Read_1          CGTTCTACCTATGATTCCGTTTAATTGAGTTGTCATATGTTAATAACGGTATCGGCGCGC
                ******************

WT              ------------------------------------------------------------
Read_1          CGCATGCGAGGAGTGCGCATATGGGCGCTCTCCCGCTTCCTCGCTCACTGACTCGCTGCG

WT              ------------------------------------------------------------
Read_1          CTCGGTCGTTCGGCTGCGTCGGGAAGCGGTACGCGCCCTGGCAGAACAGGCGGAAGTTGT

WT              ----------------------------CAGCGGCGGTGCTTTCAAAGATGTGATCACAG
Read_1          GTTGGTGGTCGGTTCGAAAAACTCCTCCCAGCGGCGGTGCTTTCAAAGATGTGATCACAG
                                            ********************************
```
