# Supplementary material for: Evaluation of Methods to Assess in vivo Activity of Engineered Genome-Editing Nucleases in Protoplasts
Source: Front Plant Sci. 2019 Feb 8;10:110. doi: 10.3389/fpls.2019.00110 (PMC6376315; doi:10.3389/fpls.2019.00110)
Supplement: Supplementary file 4 [file Data_Sheet_4.PDF]

Table S1. Media constituents used for protoplast isolation and transformation

Micro elements medium	Per L
Manganous sulfate ($MnSO_4.H_2O$)	1.69 g
Zinc Sulfate ($ZnSO_4.7H_2O$)	1.06 g
Cupric Sulfate ($CuSO_4.5H_2O$)	2.5 mg
Potassium Iodide (KI)	83 mg
Cobalt Chloride ($CoCl_2.6H_2O$)	2.5 mg
Boric acid (H_3BO_3)	620 mg
Sodium molybdate ($NaMoO_4.2H_2O$)	25 mg
**Vitamins medium**	**Per L**
Thiamine HCl	10 mg
Glycine	200 mg
Nicotinic acid	50 mg
Pyridoxine HCl	50 mg
**Culture medium**	**Per L**
95g/L Potassium nitrate (KNO_3) + 8.5g/L Potassium phosphate monobasic (KH_2PO_4) + 9.0g/L Magnesium sulfate ($MgSO_4$)	10 ml
22g/L Calcium chloride ($CaCl_2.2H_2O$)	30mL
2.8g/L Ferrous sulfate ($FeSO4.7H_2O$) + 3.7g/L EDTA disodium salt ($Na_2EDTA.2H_2O$)	5mL
Micro elements medium	5mL
Vitamins medium	5mL
Glucose	30 g
Sucrose	10 g
D-Sorbitol	50 g
Casein hydrolysate	0.5 g
Glutamine	0.1 g
Myo-inositol	0.1 g
serine	10 mg
Thiamine	10 mg
α-NAA	1.25 mg
2,4-D	0.25 mg
Zeatin	1 mg
**Pre-plasmolysis medium**	**Per L**
22g/L Calcium chloride ($CaCl_2.2H_2O$)	67 mL
Vitamins medium	10 mL
Potassium Nitrate (KNO_3)	101 mg
Potassium phosphate monobasic (KH_2PO_4)	33 mg
Magnesium sulfate ($MgSO_4$)	6 g
Mannitol	90 g
MES	0.586 g

The pH of the isolation solutions was adjusted to 5.8 with 0.1 N NaOH and HCl. Solutions are filter sterilized.

Table S2. List of primers used in the study

Primer name	Sequence (5' - 3')	Use
oligo1	P-G*T*TTAATTGAGTTGTCATATGTTAATAACGGT*A*T	Synthesize dsODNs
oligo2	C*A*AATTAACTCAACAGTATACAATTATTGCCA*T*A-P	Synthesize dsODNs
ALS1_Start-F	ATGGCGGCTGCTGCCTCACCATCT	Cloning
ALS1_Stop-R	TCAATAGGAACGTCTCCCATCACC	Cloning
EPSPS_1-F1	CCGAAGCTGAAGGTGGCTTG	Cloning
EPSPS_1-R1	CTTCCCAAGCTCACGGTGTTC	Cloning
EPSPS_1-F2	GGGAGCTAAGGGATGAAAGAAACAC	Cloning
EPSPS_1-R2	GTCTCACCACCACTCATTTCCATC	Cloning
EPSPS_2-F	CCGAAGCTGAAGGTGGCTTG	Cloning
EPSPS_2-R	CAAAGTCTTCTCCGCTTCTGCAAC	Cloning
T7ALS1F1	CCAATGGTTGACATCTGGTGG	T7EI assay for ALS1
T7ALS1R1	AAGGAGGAGCGAGAATCAAG	T7EI assay for ALS1
T7EPSPSF1	GAGCTAAGGGATGAAAGAAACACG	T7EI assay for EPSPS_98
T7EPSPSR1	CTCAACATGACAGAGAACAAGAC	T7EI assay for EPSPS_98
dsODN-F (2)	TAATTGAGTTGTCATATGTTAATAACGG	dsODN insertion PCR
dsODN-R (3)	ACCGTTATTAACATATGACAACTC	dsODN insertion PCR
ALS1_For_GS2 (1)	GCTAACAGAGCACACACTTA	dsODN insertion PCR
ALS1_Rev_GS2 (4)	GAGTGTAATACAACAGCCAC	dsODN insertion PCR
ALS1-E	TAGGAGCAATGGGATTTGG	dsODN insertion PCR
EPSPS_2-GS-For (1)	GGACTGCAAGTAGAAGATGACAG	dsODN insertion PCR
EPSPS_2-GS-Rev (4)	GCAATACCTGAAGAATAGCAGA	dsODN insertion PCR
EPSPS_1-GS-For (1)	GTGCTGCCATTACATGTCACTG	dsODN insertion PCR
EPSPS_1-GS-Rev (4)	TCAGAAGTATTTGCCACCATCC	dsODN insertion PCR
sg122_PotatoEPSPS-2 - F	GATT GCAGCAGTTGCTGTAGCTGG	CRISPR constructs for EPSPS_61
sg122_PotatoEPSPS-2 - R	AAAC CCAGCTACAGCAACTGCTGC	CRISPR constructs for EPSPS_61
sg123_PotatoEPSPS-1 - F	GATT gTGCTGTTCCTGCATTTCCA	CRISPR constructs for EPSPS_98
sg123_PotatoEPSPS-1 - R	AAAC TGGAAATGCAGGAACAGCAc	CRISPR constructs for EPSPS_98
sg751_PotatoALS1 - F	GATT GTTCTACCTATGATTCCCAG	CRISPR constructs for ALS1
sg751_PotatoALS1 - R	AAAC CTGGGAATCATAGGTAGAAC	CRISPR constructs for ALS1
sg746_PotatoALS1 - F	GATT GGGAATGGTGGTTCAAT	CRISPR constructs for ALS1
sg746_PotatoALS1 - R	AAAC ATTGAACCACCATTCCC	CRISPR constructs for ALS1
ALS1_AmF	ACACTCTTTCCCTACACGACGCTCTTCCGATCTAGAGGCTTGTGGCGTACCT	Library construction 1st PCR
ALS1_AmR	CTGGAGTTCAGACGTGTGCTCTTCCGATCTGCCAAAGACCAACGGCAAACTA	Library construction 1st PCR
Am_P5	AATGATACGGCGACCACCGAGATCTACACTCTTTCCCTACACGAC	Library construction 2nd PCR
Am_P711	CAAGCAGAAGACGGCATACGAGATGCTAACGAGTGACTGGAGTTCAGACGT	Library construction 2nd PCR
Am_P712	CAAGCAGAAGACGGCATACGAGATTGGAACAAGTGACTGGAGTTCAGACGT	Library construction 2nd PCR
Am_P714	CAAGCAGAAGACGGCATACGAGATCCGAAGTAGTGACTGGAGTTCAGACGT	Library construction 2nd PCR
Am_P735	CAAGCAGAAGACGGCATACGAGATCCTGTCATGTGACTGGAGTTCAGACGT	Library construction 2nd PCR
Am_P736	CAAGCAGAAGACGGCATACGAGATACTAGGAGGTGACTGGAGTTCAGACGT	Library construction 2nd PCR
Am_P738	CAAGCAGAAGACGGCATACGAGATCCATCCTCGTGACTGGAGTTCAGACGT	Library construction 2nd PCR
Am_P740	CAAGCAGAAGACGGCATACGAGATAATGGACGGTGACTGGAGTTCAGACGT	Library construction 2nd PCR
Am_P741	CAAGCAGAAGACGGCATACGAGATGGCAAGTTGTGACTGGAGTTCAGACGT	Library construction 2nd PCR
Am_P742	CAAGCAGAAGACGGCATACGAGATGTTCTCGTGTGACTGGAGTTCAGACGT	Library construction 2nd PCR
Am_P743	CAAGCAGAAGACGGCATACGAGATTGAAGACGGTGACTGGAGTTCAGACGT	Library construction 2nd PCR
Am_P744	CAAGCAGAAGACGGCATACGAGATCTCAGAGTGTGACTGGAGTTCAGACGT	Library construction 2nd PCR
Am_P745	CAAGCAGAAGACGGCATACGAGATCAATGTGGGTGACTGGAGTTCAGACGT	Library construction 2nd PCR
Am_P715	CAAGCAGAAGACGGCATACGAGATGGTGCGAAGTGACTGGAGTTCAGACGT	Library construction 2nd PCR
Am_P716	CAAGCAGAAGACGGCATACGAGATTGGCTTCAGTGACTGGAGTTCAGACGT	Library construction 2nd PCR
Am_P717	CAAGCAGAAGACGGCATACGAGATACACAGAAGTGACTGGAGTTCAGACGT	Library construction 2nd PCR
Am_P701	CAAGCAGAAGACGGCATACGAGATTCGCCTTAGTGACTGGAGTTCAGACGT	Library construction 2nd PCR
Am_P702	CAAGCAGAAGACGGCATACGAGATCTAGTACGGTGACTGGAGTTCAGACGT	Library construction 2nd PCR
Am_P734	CAAGCAGAAGACGGCATACGAGATACAGACCTGTGACTGGAGTTCAGACGT	Library construction 2nd PCR
Am_P718	CAAGCAGAAGACGGCATACGAGATAGGAGTCCGTGACTGGAGTTCAGACGT	Library construction 2nd PCR
Am_P719	CAAGCAGAAGACGGCATACGAGATGTCGTAGAGTGACTGGAGTTCAGACGT	Library construction 2nd PCR
Am_P720	CAAGCAGAAGACGGCATACGAGATTCTTCACAGTGACTGGAGTTCAGACGT	Library construction 2nd PCR
Am_P746	CAAGCAGAAGACGGCATACGAGATTGTTCGAGGTGACTGGAGTTCAGACGT	Library construction 2nd PCR
Am_P747	CAAGCAGAAGACGGCATACGAGATGAAGTTGGGTGACTGGAGTTCAGACGT	Library construction 2nd PCR
Am_P748	CAAGCAGAAGACGGCATACGAGATGTTCGGTTGTGACTGGAGTTCAGACGT	Library construction 2nd PCR
Am_P749	CAAGCAGAAGACGGCATACGAGATACCTAAGGGTGACTGGAGTTCAGACGT	Library construction 2nd PCR
Am_P750	CAAGCAGAAGACGGCATACGAGATCGTCAATGGTGACTGGAGTTCAGACGT	Library construction 2nd PCR
Am_P751	CAAGCAGAAGACGGCATACGAGATAAGGCGTTGTGACTGGAGTTCAGACGT	Library construction 2nd PCR
Am_P723	CAAGCAGAAGACGGCATACGAGATGTCTGTCAGTGACTGGAGTTCAGACGT	Library construction 2nd PCR
Am_P724	CAAGCAGAAGACGGCATACGAGATTTCACGCAGTGACTGGAGTTCAGACGT	Library construction 2nd PCR
Am_P725	CAAGCAGAAGACGGCATACGAGATACCTCCAAGTGACTGGAGTTCAGACGT	Library construction 2nd PCR
Am_P726	CAAGCAGAAGACGGCATACGAGATAGCCATGCGTGACTGGAGTTCAGACGT	Library construction 2nd PCR
Am_P727	CAAGCAGAAGACGGCATACGAGATGTGTTCTAGTGACTGGAGTTCAGACGT	Library construction 2nd PCR
Am_P730	CAAGCAGAAGACGGCATACGAGATCAAGGAGCGTGACTGGAGTTCAGACGT	Library construction 2nd PCR
Am_P704	CAAGCAGAAGACGGCATACGAGATGCTCAGGAGTGACTGGAGTTCAGACGT	Library construction 2nd PCR
Am_P705	CAAGCAGAAGACGGCATACGAGATAAACATCGGTGACTGGAGTTCAGACGT	Library construction 2nd PCR
Am_P706	CAAGCAGAAGACGGCATACGAGATCCGACAACGTGACTGGAGTTCAGACGT	Library construction 2nd PCR
Am_P752	CAAGCAGAAGACGGCATACGAGATAGAAGCGTGTGACTGGAGTTCAGACGT	Library construction 2nd PCR
Am_P754	CAAGCAGAAGACGGCATACGAGATCAGATCTGGTGACTGGAGTTCAGACGT	Library construction 2nd PCR
Am_P755	CAAGCAGAAGACGGCATACGAGATGAGGACTTGTGACTGGAGTTCAGACGT	Library construction 2nd PCR
Am_P731	CAAGCAGAAGACGGCATACGAGATGGACCTATGTGACTGGAGTTCAGACGT	Library construction 2nd PCR
Am_P732	CAAGCAGAAGACGGCATACGAGATCTGTAGCCGTGACTGGAGTTCAGACGT	Library construction 2nd PCR
Am_P733	CAAGCAGAAGACGGCATACGAGATTCCGAGTTGTGACTGGAGTTCAGACGT	Library construction 2nd PCR
Am_P707	CAAGCAGAAGACGGCATACGAGATGGAGAACAGTGACTGGAGTTCAGACGT	Library construction 2nd PCR
Am_P708	CAAGCAGAAGACGGCATACGAGATTGGTGGTAGTGACTGGAGTTCAGACGT	Library construction 2nd PCR
Am_P709	CAAGCAGAAGACGGCATACGAGATAGTGGTCAGTGACTGGAGTTCAGACGT	Library construction 2nd PCR

Table S3. List of constructs used in the study, target genes and sequences

	SSN - construct used	Target gene	Plant species	Accession number	Target seqience
1	AtCas9 - sg751	*ALS1 / ALS2*	*Solanum tuberosum*	PGSC0003DMG400034102 / PGSC0003DMG400007078	GTTCTACCTATGATTCCCAG*CGG*
2	HF1-Cas9 - sg751	*ALS1 / ALS2*	*Solanum tuberosum*		
3	eCas9 (1.1) - sg751	*ALS1 / ALS2*	*Solanum tuberosum*		
4	AtCas9 - sg746	*ALS1*	*Solanum tuberosum*	PGSC0003DMG400034102	GGGAATGGTGGTTCAAT*GGG*
5	HF1-Cas9 - sg746	*ALS1*	*Solanum tuberosum*		
6	eCas9 (1.1) - sg746	*ALS1*	*Solanum tuberosum*		
7	TALEN_749	*ALS1*	*Solanum tuberosum*		ACCTCATCAGGAGCACGTT
	TALEN_750	*ALS1*	*Solanum tuberosum*		CTTTGAAAGCACCGCCGCT
8	AtCas9 - sg123	*EPSPS_1*	*Solanum tuberosum*	PGSC0003DMG400026006	gTGCTGTTCCTGCATTTCCA*AGG*
9	HF1-Cas9 - sg123	*EPSPS_1*	*Solanum tuberosum*		
10	eCas9 (1.1) - sg123	*EPSPS_1*	*Solanum tuberosum*		
11	TALEN_1663	*EPSPS_1*	*Solanum tuberosum*		CCTTGGAAATGCAGGAACA
	TALEN_1664	*EPSPS_1*	*Solanum tuberosum*		ACTGTAACTGCTGCTGTC
12	AtCas9 - sg183	*EPSPS_1*	*Solanum tuberosum*		GAAGAAATCCAACTATTCCT*TGG*
13	AtCas9 - sg122	*EPSPS_2*	*Solanum tuberosum*	PGSC0003DMG400007018	GCAGCAGTTGCTGTAGCTGG*CGG*
14	HF1-Cas9 - sg122	*EPSPS_2*	*Solanum tuberosum*		
15	eCas9 (1.1) - sg122	*EPSPS_2*	*Solanum tuberosum*		
	TALENs_1661	*EPSPS_2*	*Solanum tuberosum*		GCGGCCACTAACAGCA
16	TALENs_1662	*EPSPS_2*	*Solanum tuberosum*		TAGACCTTGAATTTCCGC

Table S4. Illumina ALS1 amplicon sequencing data statistics

Reagent	Sample name	Raw read pairs (#)	Cleaned/combined reads in CRISPResso (#)	Total reads mapped (#)	Mapping rate (%)	Unmodified (#)	Unmodified (%)	NHEJ (#)	NHEJ (%)	Insertion (#)	deletion (#)	Substitution (#)	Cleaned/combined reads for ODN analysis (#)	Reads with ODN (#)	Reads with ODN (%)
CRISPR, without ODN	1-1	15,275	14488	14424	99.5582551	9952	68.9961176	4472	31.0038824	1091	3212	334	14485	0	0
	1-2	14,707	13993	13935	99.585507	9876	70.8719053	4059	29.1280947	1166	2750	293	13989	4	0.0285939
	1-3	14,320	13556	13486	99.4836235	9510	70.5175738	3976	29.4824262	1045	2813	290	13989	0	0
	2-1	14,337	13606	13539	99.5075702	9881	72.9817564	3658	27.0182436	883	2620	294	13601	0	0
	2-2	14,026	13265	13196	99.4798342	9679	73.3479842	3517	26.6520158	801	2571	286	13263	8	0.06031818
	2-3	13,971	13179	13100	99.4005615	9702	74.0610687	3398	25.9389313	820	2410	356	13164	0	0
	3-1	19,956	19171	19087	99.5618382	14215	74.4747734	4872	25.5252266	1102	3507	449	19169	3	0.01565027
	3-2	19,175	18449	18353	99.4796466	13406	73.0452787	4947	26.9547213	1092	3601	413	18440	1	0.00542299
	3-3	25,292	24285	24142	99.4111592	18089	74.9275122	6053	25.0724878	1481	4226	558	24280	1	0.00411862
CRISPR, with ODN	1-1	13,174	12588	12524	99.4915793	12229	97.6445225	295	2.35547748	28	23	252	12582	21	0.1669051
	1-2	11,668	11120	11074	99.5863309	10847	97.9501535	227	2.04984649	53	12	165	11109	16	0.14402737
	1-3	12,926	12351	12297	99.5627884	12022	97.7636822	275	2.2363178	17	46	213	12346	9	0.0728981
	2-1	16,248	15677	15584	99.4067743	15210	97.6001027	374	2.39989733	48	44	300	15670	40	0.25526484
	2-2	13,520	13002	12916	99.3385633	12549	97.158563	367	2.84143698	66	33	279	12999	38	0.29233018
	2-3	15,726	15179	15109	99.5388366	14764	97.7165928	345	2.28340724	67	28	265	15169	45	0.29665766
	3-1	17,587	16808	16662	99.131366	16240	97.4672908	422	2.53270916	36	16	383	16786	13	0.07744549
	3-2	14,059	13558	13483	99.4468211	13211	97.9826448	272	2.01735519	29	20	235	13553	13	0.09591972
	3-3	13,053	12591	12537	99.5711222	12265	97.830422	272	2.16957805	21	19	238	12586	10	0.07945336
TALENs, without ODN	1-1	14,558	13856	13761	99.3143764	12156	88.3366034	1605	11.6633966	257	1173	346	13846	0	0
	1-2	15,117	14311	14194	99.1824471	12694	89.4321544	1500	10.5678456	235	1020	397	14307	0	0
	1-3	14,846	14097	13975	99.1345676	12487	89.352415	1488	10.647585	266	997	379	14086	0	0
	2-1	11,092	10220	10130	99.1193738	8667	85.5577493	1463	14.4422507	233	1057	323	10209	0	0
	2-2	11,625	10813	10716	99.1029317	9403	87.7472938	1313	12.2527062	273	915	271	10803	0	0
	2-3	6,546	6092	6028	98.9494419	5153	85.4844061	875	14.5155939	179	616	167	6085	0	0
	3-1	49,572	46299	45930	99.2030065	39889	86.8473764	6041	13.1526236	1013	4371	1185	46248	0	0
	3-2	14,781	13821	13701	99.131756	11783	86.0010218	1918	13.9989782	316	1409	377	13810	0	0
	3-3	12,175	11330	11233	99.1438658	9814	87.3675777	1419	12.6324223	287	925	344	11314	0	0
TALENs, with ODN	1-1	9,522	8551	8441	98.7136007	8232	97.52399	209	2.47600995	31	36	151	8537	29	0.33969779
	1-2	8,658	7771	7688	98.9319264	7467	97.1253902	221	2.87460978	22	50	158	7758	12	0.15467904
	1-3	7,501	6735	6656	98.827023	6496	97.5961538	160	2.40384615	31	31	135	6720	25	0.37202381
	2-1	13,219	12370	12225	98.8278092	11917	97.4805726	308	2.5194274	43	73	211	12336	33	0.26750973
	2-2	13,217	12336	12173	98.6786641	11942	98.1023577	231	1.89764232	54	42	183	12320	38	0.30844156
	2-3	10,056	9380	9276	98.891258	9059	97.6606296	217	2.33937042	46	49	146	9370	35	0.37353255
	3-1	15,340	14624	14506	99.1931072	14115	97.3045636	391	2.69543637	44	62	297	14611	33	0.22585723
	3-2	13,996	13340	13221	99.107946	12906	97.6174268	315	2.38257318	30	46	256	13333	20	0.15000375
	3-3	14,182	13548	13399	98.9002067	13110	97.8431226	289	2.15687738	50	38	216	13523	40	0.29579235
Only with ODN	1-1	13,497	12754	12694	99.5295594	12467	98.2117536	227	1.78824642	12	6	211	12748	0	0
	1-2	12,686	12019	11956	99.4758299	11718	98.0093677	238	1.99063232	12	16	210	12014	0	0
	1-3	14,853	14141	14089	99.6322749	13824	98.1191	265	1.88089999	8	3	254	14139	0	0
	2-1	20,340	19708	19609	99.4976659	19295	98.3986945	314	1.60130552	16	10	290	19692	0	0
	2-2	17,713	17062	16966	99.4373461	16583	97.7425439	383	2.25745609	8	6	370	17052	0	0
	2-3	20,139	19412	19319	99.5209149	18954	98.1106683	365	1.88933175	16	8	344	19409	0	0
	3-1	12,920	12476	12421	99.5591536	12110	97.4961758	311	2.50382417	5	36	273	12470	0	0
	3-2	16,803	16188	16087	99.376081	15755	97.9362218	332	2.06377821	14	33	286	16177	0	0
	3-3	16,555	15976	15890	99.4616925	15582	98.061674	308	1.93832599	14	30	264	15967	0	0
